# Supplementary material for: Weighting of risk factors for low birth weight: a linked routine data cohort study in Wales, UK
Source: BMJ Open. 2023 Feb 10;13(2):e063836. doi: 10.1136/bmjopen-2022-063836 (PMC9923297; doi:10.1136/bmjopen-2022-063836)
Supplement: Supplementary data [file bmjopen-2022-063836supp003.pdf]

Weighting of risk factors for low birth weight

Aim

The WHO designates infants weighing 2500 g or less as 'low birth weight' (LBW). The main factors associated with LBW are due to intra-uterine growth restriction, or prematurity. This report examines:

(a) the risk factors associated with low birth weight and

(b) the relative weighting of importance of the risk factors in determining LBW. This includes

1. The strength of the association and
2. The number/prevalence of infants exposed to each risk factor in RCT and in Wales.
3. The number/prevalence of infants exposed to each risk factor at birth, in RCT flying start and non-flying start areas.

According to Welsh Government statistics 5.6% of singleton births were low birthweight in 2018.

Method

In order to examine factors associated with low birth weight (LBW), an initial scoping search was undertaken and a relevant piece of work by Johnson et al (2017) was identified, which was published in 2016 in collaboration with Public Health Wales. This piece of work aimed to understand the contribution of modifiable risk factors to the burden of LBW and identify prevalence data from the population of Wales. The study examined research from 2006-2013, but also reported on research prior to 2006 which was conducted by the Institute of Health Economics.

This current piece of work, commissioned by Public Health Wales, will build on the work by Johnson et al as a framework.

Search criteria: Firstly, any systematic reviews published since 2013 focusing on the risk factors identified in Johnson et al will be identified and the odds ratios of more recent studies conducted since their review will be noted in the table. Secondly, any systematic reviews published since 2010 will be explored for all additional risk factors not identified in Johnson et al. If systematic reviews cannot be found for these risk factors, a further search will be conducted to identify other types of study including cohort studies or case control studies.

Following this, a search will be conducted for prevalence of each risk factor. Where available, Welsh data will be reported. If welsh data is not found, then UK data will be presented, followed by evidence reviews or population cohort-based studies. Where pregnancy specific data is not able to be found, general population prevalence of each risk factor will be reported.

**Risk factor**	Risk range in research	Selected Risk size (OR or RR)	Evidence associated with presented OR/RR	Prevalence	Prevalence in RCT
Heroin/methadone	1.74-4.61	3.28	Hulse et al 1997 in Johnson et al: meta analyses	0.1% (CSEW 2018) General Population	
Cocaine	2.15-4.42	2.85<br><br>**2.80 (2.39-3.27)**	Moretti et al 2001 in Johnson et al: meta analyses<br>Dos Santos et al 2018: Systematic review crack cocaine use during pregnancy	2.6% powder cocaine and 0.1% powder cocaine-General population (CSEW 2018)	
Smoking in pregnancy	1.43-2.00	1.9<br><br>**2.0 (1.77-2.26)**<br><br>**1.91 (1.56-2.34)**	Walsh 1994.in Johnson et al<br><br>Pereira et al 2017: Systematic review and meta-analysis<br>Flower et al 2013: UK millennium cohort study	17.8% Public Health Wales (2017/18)<br><br>17.9% Welsh Government 2018	22.4% Welsh Government (Cwm Taf HB 2017/18)
Severe gum disease	1.5-1.8	1.8<br><br>**1.7 (1.3-2.1)**	Corbella et al 2012 in Johnson et al: systematic review and meta-analysis<br>Daalderop et al 2018: Overview of systematic reviews	40% (some degree of periodontal disease) Lieff 2004.<br><br>*Studies produce a wide variation in prevalence's (11% to 100%	
Cannabis	0.7-1.7	1.7<br><br>**1.77 (1.04-3.01)**	Hayatbakhsh et al 2012 in Johnson et al: cohort study<br>Gunn et al 2015: systematic review and meta analysis	7.2% (CSEW 2018) General population	
Low BMI	1.64-1.7	1.64	Han et al. 2011 in Johnson et al: cross sectional analyses	4.5% Underweight Public Health England (2019)	

Intimate partner violence	1.5-1.53<br><br>1.05-1.31<br><br>1.68-2.65	1.53<br><br>1.18<br><br>2.11	Shah et al 2011 in Johnson et al: Systematic review and meta analyses<br><br>Hill et al 2016: Systematic review and meta analysis<br><br>Donovan et al 2016: Systematic Review	5.7% CSEW 2019, adults experienced domestic abuse in the last year<br>General Population	
Chlamydia	0.19-1.52	1.52<br><br>**1.34 (1.21-1.48)**	De Attayde Silva 2011<br><br>Olson-chen et al 2018	1.5% in women<br>3.1% in women aged 16-24<br>(Sonnenberg et al 2013) UK General Population<br><br>12% in pregnancy (Junghans et al 2016) UK	
Bacterial vaginosis	1.43-2.02	1.43	**Flynn et al 1999** in Johnson et al: meta analysis	7.1% (Desseauve et al 2012) in French pregnant population	
Anaemia	1.29-1.94	1.29<br><br>1.23 (1.06-1.43)	**Ref in Johnson et al** Haider et al 2013. : systematic review and meta-analysis<br><br>**Figuerido et al 2018:** Systematic review and meta-analysis	24% (Barroso et al 2011) UK Population<br><br>UK: 46% at booking or 28-week checks (Nair et al, 2017).	
Environmental tobacco smoke exposure	1.22-1.38	1.32	Bee et al 2008 in Johnson et al: systematic review and meta-analysis	Not available	
Teenage pregnancy	1.1-2.9	1.17	Haldre et al. 2007 in Johnson et al	2.9% (aged <20) ONS 2018	
Inter-pregnancy interval (1-5m)	1.06-3.54	1.61	Conde-Agudelo 2006 in Johnson et al : meta analysis	UK population cohort study	

Inter preg interval (6-11m)	1.06-3.54	1.14	Conde-Agudelo 2006 in Johnson et al : meta analysis	17.5% of women had interval pregnancy between 0-11 months on 1st and 2nd pregnancy and 19.7% on 2nd and 3rd pregnancy Ziauddeen et al (2019)	
Inter preg interal (12-18m)	1.06-3.54	1.06	Conde-Agudelo 2006 in Johnson et al : meta analysis		
Alcohol	0.64-1.27	1.06<br><br>2.0 (SGA)	Patra et al 2011 in Johnson et al: systematic review and meta analyses<br><br>Nykjaer et al 2014: British cohort	UK-41.3% (32.9-49) Popova et al (2017) Any alcohol use during pregnancy<br><br>Over 50% of women in a UK sample reported alcohol intakes in the first trimester above DH guidelines (<=2 units per week). Nykjaer et al 2014	
**Others:**					
Maternal anxiety during pregnancy		1.80 (1.48- 2.18)	Griogoriadis et al 2018: Systematic review and meta analysis	24.1% Welsh Government (2018): mental health condition reported at initial assessment	
Maternal Stress during/before pregnancy		1.68 (1.19-2.38)	Molina Lima et al 2018: Systematic review and meta analysis of cohort studies.		
Maternal depression during pregnancy		1.39 (1.22-1.58)	Dadi et al 2019: Umbrella review		

Antidepressant use in pregnancy		Limited effect (evidence issues)<br><br>1.44 (1.21-1.70)	Prady et al 2018: Systematic review<br><br>Huang 2014- Meta-analysis	4.5% SSRI prescribing during pregnancy in Wales (Charlton 2014)	
Pre-pregnancy BMI		Underweight and LBW (1.47, 1.27-1.71)<br><br>Overweight and LBW: 0.79 to 1.01. After publication bias accounted for: 0.95, 0.85-1.07).<br><br>Overweight and preterm birth (1.24, 1.13 to 1.37)	Yu et al 2013: Systematic review and meta analysis<br><br>McDonald et al 2010: Systematic review and meta analysis.	28.0% Welsh Government (2018) : women obese (BMI 30+) at their initial assessment.	
Pregnancy weight gain		Low gestational weight gain and LBW 1.84 (1.71–1.99)<br><br>Low gestational weight gain and SGA 1.51 (1.39–1.63)	Han et al 2011<br><br>Goldstein et al 2018		

		High gestational weight gain and LBW<br>0.64 (0.53-0.78)	Mcdonald et al 2011		
Caffeine intake during pregnancy		Limited evidence<br><br>Low intake (50 to 149mg/day): 1.13 (1.06-1.21)<br><br>Moderate intake (150 to 349mg/day 1.38 (1.18-1.62)<br><br>High intake (>=350mg/day) 1.60 (1.24-2.08	Jahanfar et al 2015: Cochrane systematic review<br><br>Chen et al 2014: Systematic review and dose response meta-analysis.	No recent data available	
Area deprivation (neighbourhood and individual social class)		Area: 1.81 (1.71 - 1.92)<br>Social class (1.79 (1.43 to 2.24)<br><br>LBW (1.11, 1.02-1.20)<br><br>SGA: 1.31 (1.28-1.34)	Weightman et al 2012: UK specific systematic review<br><br>Metcalf et al 2011<br><br>Vos et al 2014		
Vitamin D supplementation in pregnancy	0.35-0.87<br><br>0.22-0.74	0.50<br><br>0.40 **	Palacios et al 2019: Cochrane review<br><br>Maugeri et al 2019 Systematic review of RCTs.		

			De Regil et al 2016: Cochrane review		
Folic acid supplementation		No conclusive evidence<br>RR 0.83, 0.66 - 1.04<br><br>No effect	Lassi et al 2013: cochrane systematic review<br><br>Lopes et al 2017: overview of systematic reviews	31% took folic acid prior to conception Bestwick et al 2014	
Air pollution		1.03–1.21	Guo et al 2019: systematic review and meta analysis		
Maternal education level		0.67 (0.51-0.88), High maternal education	Silvestrin et al 2013		
Maternal Age		Mixed findings	Goisis et al 2017: Finnish population data linkage study<br>Goisis et al 2018: UK cross cohort comparison study	Age 40+ 4% ONS 2018	
Paternal factors		Advance paternal age<br>Prolonged lead exposure and low paternal education may be associated	Shah et al 2010: Systematic review		

Summaries of above reported research:

Maternal depression during pregnancy

Dadi et al (2019): Global burden of antenatal depression and its association with adverse birth outcomes: an umbrella review

This umbrella review pooled estimates of three systematic reviews exploring the association between depression during pregnancy (measured using a validated screening or diagnostic tool) and LBW. Results showed that risk of LBW was 1.39 times higher among pregnant mothers with antenatal depression. Limitations of this review were that studies used different depression screening tools with different cut off values and there were different study designs among primary studies.

Antidepressant use during pregnancy:

Prady et al (2018): A systematic review of maternal antidepressant use in pregnancy and short- and long-term offspring's outcomes

This review evaluated the research which compared LBW and other outcomes for children whose mothers took antidepressants during pregnancy compared to those whose mothers had common mental disorders, or symptoms, but did not take anti-depressants during pregnancy. Four cohort studies were included with an outcome of LBW. Meta-analysis was unable to be conducted because of wide variation in study design and high risk of bias among studies. The authors concluded that there was little evidence to indicate that using antidepressants in pregnancy causes infants to have LBW (after adjusting for gestational age).

Authors stated limitations stemming from difficulty in being certain that any effects believed to be due to exposure to antidepressants did not reflect differences in social or clinical characteristics of women who continue antidepressants in pregnancy compared to those who discontinue or do not take them at all. They advocated for more consistency over how studies assess exposure variables, mental health disorders, outcomes and treatments.

An earlier review by Huang et al 2014 however found antidepressant use increased the risk of LBW and PTB but it involved a mixture of studies with different groups as controls and limited studies in the analysis controlled for severity and persistence of depression.

Maternal anxiety during pregnancy:

Grigoriadis et al (2018): Maternal Anxiety During Pregnancy and the Association With Adverse Perinatal Outcomes: Systematic Review and Meta-Analysis.

This systematic review and meta-analysis identified 11 studies using the outcome of LBW and showed the association with maternal anxiety was significant ($P < .00001$. Antenatal anxiety associated with increased odds of LBW, premature birth (1.54), and increased odds for small for gestational age (1.48). Studies which reported on clinical diagnosis of anxiety as their outcome produced a higher odds ratio (2.09) compared to studies using self-report measures (1.42) suggesting the severity of anxiety to be important in predicting low birth weight.

The limitations of this study relate to methodological issues of the primary research included in the review. The definition of anxiety by self-report varied across studies with the regards to the scales and cut off scores used. Even studies which used the State-Trait Anxiety Inventory (STAI), a commonly used measure of antenatal anxiety used different cut off scores. The review also included all types of anxiety disorders and was not specific as to particular disorders.

Maternal stress during pregnancy:

Molina Lima et al (2018): Is the risk of low birth weight or preterm labor greater when maternal stress is experienced during pregnancy? A systematic review and meta-analysis of cohort studies

This systematic review and meta-analysis included 8 cohort studies which proved eligible for inclusion in the review. Results of the review showed a significant association between antenatal stress exposure and rates of LBW. However, no statistically significant difference was found between non exposed and exposed groups relating to preterm labour. The review advocated for further studies with adequate sample size and longer follow up time.

Caffeine intake during pregnancy:

Jahanfar et al (2015) Effects of restricted caffeine intake by mother on fetal, neonatal and pregnancy outcomes: Cochrane systematic review

This review involved only one eligible study which involved 1207 pregnant women recruited before 20 weeks gestation. The first group regularly drank 3 cups of instant coffee (caffeinated). These were compared to the second group who drank the same volume of decaffeinated instant coffee. This had no effect on SGA, birth weight or preterm birth. They suggested there is currently insufficient evidence from high quality RCTs to evaluate the effect of restricted caffeine intake during pregnancy on fetal outcomes.

Chen at el (2014): Maternal caffeine intake during pregnancy is associated with risk of low birth weight: a systematic review and dose-response meta-analysis

This systematic review identified 9 prospective studies with LBW as a binary outcome variable (90,747 participants and 6,303 cases). Higher caffeine intake during pregnancy was associated with a higher risk of LBW. This increased with increasing levels of caffeine intake, suggesting a dose response. The study suggested that the risk of LBW may be elevated even for caffeine intakes below the recommended maximum limit of current guidelines for pregnant women (300mg.day by WHO and 200mg/day by Nordic and American College). Limitations lie in potential biases including that of confounding by smoking or pregnancy symptoms affecting the association seen. WHO class this review as low to moderate certainty evidence.

Pregnancy weight gain:

Low gestational weight gain and LBW:

Han et al (2011): Low gestational weight gain and the risk of preterm birth and low birthweight: a systematic review and meta-analyses

Low gestational weight gain and the risk of preterm birth and low birthweight: a systematic review and meta-analyses

Singleton infants born to women with low total pregnancy weight gain had higher risks of LBW and higher risks of PTB. The lower the gain, the higher the risks were. Limitations stem from few studies providing adjusted analyses or examining the combined impact of gestational weight gain and maternal weight. Authors state that the impact of low pregnancy weight gain in underweight women compared to normal weight and obese women needs more research as there may be less of a risk in heavier women

Low gestational weight gain and small for gestational age:

Goldstein et al (2018): Gestational weight gain across continents and ethnicity: systematic review and meta-analysis of maternal and infant outcomes in more than one million women.

Seven studies for USA/Europe were included in this analysis. Gestational weight gain below that of the guidelines was associated with a higher risk for small for gestational age. This study also focused on differences in ethnicity across studies but reported higher risks across all ethnicities.

High gestational weight gain and LBW:

Mcdonald et al (2011): High Gestational Weight Gain and the Risk of Preterm Birth and Low Birth Weight: A Systematic Review and Meta-Analysis

This review contained 38 studies but these mainly presented unadjusted data. Women with high total gestational weight gain had lower unadjusted risks of LBW and PTB. However, high weekly GWG was associated with increased risk. Authors said more unadjusted studies are urgently needed and more syudies with obese women and suggest the potential benefits of high gestational weight gain need to be considered against maternal risks and infant risks including high birth weight.

Pre-pregnancy BMI:

Yu et al (2013): Pre-Pregnancy Body Mass Index in Relation to Infant Birth Weight and Offspring Overweight/Obesity: A Systematic Review and Meta-Analysis

45 studies of medium to high quality were included in this review. In comparison to normal weight mothers, pre-pregnancy underweight increased the risk of low birth weight and small for gestational age. Pre-pregnancy overweight or obesity increased the risk of high birth weight (1.53,1.44-1.63) and being large for gestational age. Limitations lie in that there may be other factors not included that may mediate the association which include but are not limited to maternal age, gestational hypertension, and smoking. Authors advocate for these factors to be addressed in future studies.

Mcdonald et al (2010): Overweight and obesity in mothers and risk of preterm birth and low birth weight infants: systematic review and meta-analyses

This review found that the overall risk of LBW was decreased in women who were overweight and obese (0.8, 0.75 to 0.95). The overall risk of PTB was similar in overweight and obese women and women of normal weight but the risk of PTB before 32 weeks and induced preterm birth before 37 weeks was

increased in overweight and obese women. After they accounted for publication bias, the apparent protective effect of OW and obesity on LBW no longer remained, whereas risk of PTB was significantly higher in overweight and obese women (1.24,1.13 -1.37). Limitations stem from many of the included studies not adjusting for confounding variables such as gestational weight gain, socioeconomic status and smoking status. Authors argue that pre-pregnancy BMI more important than gestational weight gain.

Deprivation:

Weightman et al (2012): Social inequality and infant health in the UK: systematic review and meta-analyses

Both being in the most deprived neighbourhood and low social class increased the odds of LBW infants. Limitations include studies varying in comparison of deprivation levels and authors noted the effects of deprivation may vary between the areas where primary research studies were carried out.

Vitamin D supplementation during pregnancy

Maugeri et al (2019): Effects of Vitamin D Supplementation During Pregnancy on Birth Size: A Systematic Review and Meta-Analysis of Randomized Controlled Trials.

The meta-analysis of RCTs showed a significant positive effect of maternal vitamin D supplementation on the risk of being born small for gestational age. However, researchers suggest more RCTs are needed to better understand risks and benefits of such interventions.

**An earlier Cochrane review by De Regil et al (2016) suggested that whilst vitamin D supplementation during pregnancy may reduce the risk of having a low birth weight infant, results show that when vitamin D and Calcium are combined there is an increased risk of premature birth and data on adverse effects are not well reported.

Folic acid supplementation:

Lassi et al (2013): Folic acid supplementation during pregnancy for maternal health and pregnancy outcomes

This Cochrane review included 4 studies which looked at the association between folic acid supplementation during pregnancy and low birthweight as part of a wider group of outcomes. No impact was seen on reducing low birth weight.

A later overview of systematic reviews by Lopes et al (2017) also found folic acid supplementation did not alter the risk of premature birth or LBW.

Air pollution:

Guo et al (2019): Ambient air pollution and adverse birth outcomes: a systematic review and meta-analysis:

This study found that when mothers were exposed to CO, NO_2, NO_x, O_3, $PM_{2.5}$, PM_{10}, and SO_2 throughout pregnancy, there was significant association with LBW. They did suggest that future meta-analyses should take into account the extent of interactions between differing pollutants and explore issues such as economic status and disease history not accounted for in this analysis.

A study was carried out in 2014 by Hammen et al specific to the UK which found small increased risks of SGA with exposure to high concentrations of PM_{10} during pregnancy and similar effects for NO_2, $PM_{2.5}$ and CO in later pregnancy, with this association found particularly among female infants.

Maternal education level

Silvestrin et al (2013): Maternal education level and low birth weight: a meta-analysis.

High maternal education showed a 33% protective effect against low birth weight, whereas medium degree of education showed no significant protection when compared to low maternal education.

Maternal age:

Goisis et al (2017): Advanced Maternal Age and the Risk of Low Birth Weight and Preterm Delivery: a Within-Family Analysis Using Finnish Population Registers
Goisis et al (2018): Secular changes in the association between advanced maternal age and the risk of low birth weight: A cross-cohort comparison in the UK.
Findings regarding the impact of older maternal age on low birth weight have been mixed. The Finnish study by Goissis et al (2017) found that between families the risk of LBW was 1.1 (0.8-1.4) for those aged 35-39 and 2.2 (1.4-2.9) for those aged 40+. However, when they looked within families, this association disappeared. A UK cross cohort study by Goisis et al (2018) also found that in the later birth cohorts the effect of maternal age on LBW was less.

Paternal factors

Shah (2019): Paternal factors and low birthweight, preterm and small for gestational age births: a systematic review

This study identified paternal age and height to be associated with LBW. They also suggested heavy and prolonger exposure to lead aswell as low paternal education may be associated with LBW but advocated for more studies in this field.

Cocaine

Dos Santos et al (2018) Maternal, fetal and neonatal consequences associated with the use of crack cocaine during the gestational period: a systematic review and meta-analysis:

This study was specific to crack cocaine and included 10 studies showing crack cocaine use during pregnancy to be significantly associated with preterm birth (OR: 2.22, 1.59–3.10),, small for gestational age (4.00; 1.74–9.18) and low birth weight (2.80; 95% CI 2.39–3.27).

Smoking

Pereira at el (2017) Maternal Active Smoking During Pregnancy and Low Birth Weight in the Americas: A Systematic Review and Meta-analysis:

This review and meta analysis found similar odds ratios to that reported in the previous study by Walsh et al (1994) in the Johnson review. This review was however specific to the Americas.

Flower et al (2013) Pregnancy planning, smoking behaviour during pregnancy, and neonatal outcome: UK millennium cohort study

This is an earlier study which may be of greater relevance in terms of population. This study again found a similar odds (1.91; 1.56-2.34) for LBW for babies of mothers who were smoking just before pregnancy. Women who quit or reduced the amount they smoked during the pregnancy lowered the risk of LBW by one third compared with those whose smoking status did not change.

Gum Disease

Daalderop et el (2017): Periodontal Disease and Pregnancy Outcomes: Overview of Systematic Reviews

This review of reviews found a similar relative risk ratios to that generated by Corbella et al (2012) in the Johnson et al review. With relative risk of LBW at 1.7 (1.3-2.1), preterm birth (1.6; 1.3-2.0) and preterm low birth weight (3.4, 1.3-8.8). The review concluded that there is consistent evidence from systematic reviews indicating pregnant women with periodontal disease are at increased risk of having a LBW baby.

Cannabis

Gunn et al (2014) Prenatal exposure to cannabis and maternal and child health outcomes: a systematic review and meta-analysis

This systematic review , again found similar odds ratios of mums using cannabis during pregnancy (1.77; 1.04-1.31) to that generated by a cohort study by Hayatbakhsh et al 2012 in Johnson et al.

Intimate Partner Violence

Hill et al (2016) A Systematic Review and Meta-Analysis of Intimate Partner Violence During Pregnancy and Selected Birth Outcomes

Donovan et al (2016) Intimate Partner Violence During Pregnancy and the Risk for Adverse Infant Outcomes: A Systematic Review and Meta-Analysis

These two reviews carried out in 2016 found different risk ratios for the effect of intimate partner violence on low birth weight. Donovan et al (2016) found OR of 2.11 for LBW but 1.37 for SGA which was only marginally significant although meta analysis was on fewer studies. They also called to more studies examining this association as suggested a large degree of heterogeneity in LBW studies. The review by Hill et al (2016) reported much lower OR of 1.18.

Chlamydia

Olson-Chen et al (2018) Chlamydia trachomatis and Adverse Pregnancy Outcomes: Meta analysis of Patients with and without infection

The authors of this review suggest that chlamydia in pregnancy is associated with small increases in the odds of adverse pregnancy outcomes. The odds of LBW (1.34; 1.21-1.48) and small for gestational age (1.14; 1.05-1.25) were significant but authors suggest the literature is complicated by heterogeneity and associations may not hold in higher quality prospective studies.

Anemia

Figuerido et al (2018) Maternal Anemia and Low Birth Weight: A Systematic Review and Meta-Analysis

This review found a similar odds ratio (1.23) to that reported by Haider et al (2013) in Johnson review with maternal anemia a risk factor for LBW.

Alcohol

Nykjaer et al (2014) Maternal alcohol intake prior to and during pregnancy and risk of adverse birth outcomes: evidence from a British cohort

This cohort study found that over half of pregnant women in the first trimester reported alcohol intake above the Department of Health guidelines of <=2 units per week. Consuming alcohol in the first trimester was the most sensitive to developing foetus. Results showed that even women complying with government alcohol guidelines in this period were still at significantly higher risk of having LBW babies and preterm birth compared to non-drinkers.

Interventions for prevention of Low Birth Weight

East et al 2019 Cochrane review examined programmes offering social support during pregnancy compared with routine care for women at increased risk of low birth weight. Offering additional social support (emotional , informational and instrumental) slightly reduced the number of babies with low birth weight (0.94, 0.86-1.04) but any effect was not large.

Chamberlain et al 2017 : A Cochrane review of psychosocial interventions (counselling, health education, feedback, incentives, social support, exercise and dissemination) to stop smoking in pregnancy found counselling, feedback and incentives seem to be effective at increasing the proportion of women who stop smoking in late pregnancy. However, they suggest the context of the interventions need careful consideration. The effect of health education and social support was less clear. Woman who received psychosocial interventions had a 17% reduction in low birth weight infants.

Temel et al 2014-Evidence based preconception lifestyle interventions.

This research suggests that the list regarding interventions for which there is substantial evidence of effectiveness when applied in the preconception period is relatively short. For alcohol, evidence is lacking. Nutrition interventions show effectiveness in terms of dietary change and birth weight. Smoking interventions were shown to be effective in smoking reduction in the preconception period and individual and collective interventions to increase use of folic acid use had positive effects on behaviour change.

Thangaratinam et al 2012 Effects of interventions in pregnancy on maternal weight and obstetric outcomes: meta-analysis of randomised evidence:

This meta-analysis concluded dietary and lifestyle interventions in pregnancy are effective in reducing gestational weight gain without any adverse effect on the risk of infants born SGA. Dietary interventions were associated with the greatest reduction in pregnancy weight gain compared with physical activity and a mixed approach. Diet significantly reduced the risk of preterm birth compared with any other intervention. The rating of evidence quality in this analysis was moderate.
