# Supplementary material for: Weighting of risk factors for low birth weight: a linked routine data cohort study in Wales, UK
Source: BMJ Open. 2023 Feb 10;13(2):e063836. doi: 10.1136/bmjopen-2022-063836 (PMC9923297; doi:10.1136/bmjopen-2022-063836)
Supplement: Supplementary data [file bmjopen-2022-063836supp001.pdf]

Supplementary Table1: Variables and their source datasets

Variables	NCCHD	Description
WOB	Welsh Demographic Service (WDS) dataset	Week of birth, the first Monday of the birth week
Gender	National Community Child Health Database (NCCHD)	Sex of the child
Maternal age	NCCHD	Maternal age at child's birth
Gestational age	NCCHD	Gestational age in week (between 22 and 45 weeks)
Birth weight	NCCHD	Birth weight in gm (max 5000 gm)
Birth order	Derived	It's based on the order of the child in the family using their week of birth and DENSE_RANK function. It ranks the children same if they are non-singleton children and sharing same WOB.
Pregnancy interval	Derived	Pregnancy interval, in week format, was derived using the birth order, week of birth (the Monday of the week of date of birth), of the previous child and the current child, maternal identifier, and the multiple birth flag.
Multiple birth flag	Derived	Using WOB, encrypted maternal identifier and the birth order, a binary variable – 'multiple birth flag' was derived to distinguish between singleton and non-singleton birth.
Mother weight (kg)	Derived	The maternal weight during pregnancy was obtained from MID and WLGP. The final maternal weight variable was derived following cleaning and harmonising it with the source variables which includes removing and recoding missing, erroneous, and inconsistent records.
Maternal smoking	NCCHD, Maternity Indicators Dataset (MIDS), Welsh Longitudinal General Practice Dataset (WLGP) - Welsh Primary Care	A cleaned and harmonised variable of maternal smoking during pregnancy was created based on the data obtained from three sources.
WIMD	WDS	Welsh Index of Multiple Deprivation (1: most deprived; 5: least deprived)

Diabetes (GP)	WLGP	Mothers' diabetes record from GP during pregnancy
Diabetes (Hospital)	Patient Episode Dataset for Wales (PEDW)	Mothers' diabetes record from hospital during pregnancy
Depression (GP)	WLGP	Mothers' depression record from GP during pregnancy
Depression (Hospital)	PEDW	Mothers' depression record from hospital during pregnancy
Serious Mental Illness	WLGP	Mothers' serious mental illness related record from GP during pregnancy
Anxiety (GP)	WLGP	Mothers' anxiety record from GP during pregnancy
Anxiety (Hospital)	PEDW	Mothers' anxiety record from hospital during pregnancy
Anti-depressant medication	WLGP	Mothers' receiving anti-depressant medication from GP during pregnancy
Vitamin D	WLGP	Mothers' receiving Vitamin D from GP during pregnancy
FOLIC Acid	WLGP	Mothers' receiving Folic acid from GP during pregnancy
Anaemia (GP)	WLGP	Mothers' Anaemia record from GP during pregnancy
Anaemia (Hospital)	PEDW	Mothers' Anaemia record from hospital during pregnancy
Alcohol (GP)	WLGP	Mothers' alcohol record from GP during pregnancy
Anaemia (Hospital)	PEDW	Mothers' alcohol record from hospital during pregnancy
Assault	PEDW	Mother admitted to hospital during pregnancy for assault
Substance misuse	Substance Misuse Dataset (SMDS)	Mother receiving substance misuse treatment during pregnancy from SMD database
Living area	WDS	Living area during child's birth
Local authority	WDS	Local authority of the living area

Supplementary Table 2: Characteristics of the study population

Variables	Overall training set (n = 639,163)		Overall test set (n = 54,214)		Overall (n = 693,377)	
Gender						
Girl	311,193	48.69%	26,689	49.23%	337,882	48.73%
Boy	327,920	51.30%	27,522	50.77%	355,442	51.26%
Unknown/NULL	50	0.01%	<5	-	-	-
Maternal age						
Less than 19	46,668	7.30%	5,156	9.51%	51,824	7.47%
20-24	133,792	20.93%	13,149	24.25%	146,941	21.19%
25-29	181,233	28.35%	16,454	30.35%	197,687	28.51%
30-34	170,957	26.75%	12,938	23.86%	183,895	26.52%
35 and above	105,869	16.56%	6,513	12.01%	112,382	16.21%
Unknown/NULL	644	0.10%	<5	-	-	-
Birth order						
1st child	319,093	49.92%	26,552	48.98%	345,645	49.85%
2nd child	212,155	33.19%	18,672	34.44%	230,827	33.29%
3rd child	74,724	11.69%	6,407	11.82%	81,131	11.70%
4th or above	33,191	5.19%	2,583	4.76%	35,774	5.16%
Pregnancy interval						
Only/First child	319,093	49.92%	26,552	48.98%	345,645	49.85%
less than 1 year	5,708	0.89%	526	0.97%	6,234	0.90%
1-2 years	67,986	10.64%	5,333	9.84%	73,319	10.57%
2-5 years	162,590	25.44%	13,519	24.94%	176,109	25.40%
5-7 years	41,060	6.42%	4,081	7.53%	45,141	6.51%
7-10 years	27,161	4.25%	2,707	4.99%	29,868	4.31%
Above 10 years	15,565	2.44%	1,496	2.76%	17,061	2.46%
Gestational age (week)						
1: Extremely pre-term: <28 week	2,361	0.37%	208	0.38%	2,569	0.37%
2: Very pre-term: 28-31	5,296	0.83%	565	1.04%	5,861	0.85%
3: Pre-term: 32-36	38,565	6.03%	3,664	6.76%	42,229	6.09%
4: term: 37-42	577,104	90.29%	49,540	91.38%	626,644	90.38%
5: Late term: 43-45	3,909	0.61%	91	0.17%	4,000	0.58%
Unknown/NULL	11,928	1.87%	146	0.27%	12,074	1.74%
Birth weight (gm)						
1: BW≤ 1,000	3,010	0.47%	246	0.45%	3,256	0.47%
2: BW 1,001 - 1,500	4,372	0.68%	445	0.82%	4,817	0.69%
3: BW 1,501 - 2,499	39,143	6.12%	3,923	7.24%	43,066	6.21%
4: BW 2,500 - 4,000	521,172	81.54%	44,524	82.13%	565,696	81.59%
5: BW 4,001 - 4,500	61,658	9.65%	4,413	8.14%	66,071	9.53%
6: BW 4,501 - 5000	9,808	1.53%	663	1.22%	10,471	1.51%
Low Birth Weight (LBW)						
nLBW	594,408	93.00%	49,734	91.74%	644,142	92.90%
LBW	44,755	7.00%	4,480	8.26%	49,235	7.10%

Multiple birth flag						
Singleton	619,458	96.92%	52,583	96.99%	672,041	96.92%
Non-singleton	19,705	3.08%	1,631	3.01%	21,336	3.08%
Maternal smoking						
No	502,914	78.68%	41,344	76.26%	544,258	78.49%
Yes	136,249	21.32%	12,870	23.74%	149,119	21.51%
Welsh Index of Multiple Deprivation						
1 (most deprived)	147,204	23.03%	17,946	33.10%	165,150	23.82%
2	118,271	18.50%	17,711	32.67%	135,982	19.61%
3	117,242	18.34%	7,089	13.08%	124,331	17.93%
4	104,056	16.28%	3,646	6.73%	107,702	15.53%
5 (least deprived)	94,190	14.74%	6,242	11.51%	100,432	14.48%
Unknown/NULL	58,200	9.11%	1,580	2.91%	59,780	8.62%
Diabetes GP (mother)						
No	638,628	99.92%	54,149	99.88%	692,777	99.91%
Yes	535	0.08%	65	0.12%	600	0.09%
Diabetes PEDW (mother)						
No	636,104	99.52%	53,978	99.56%	690,082	99.52%
Yes	3,059	0.48%	236	0.44%	3,295	0.48%
Depression GP (mother)						
No	631,230	98.76%	53,323	98.36%	684,553	98.73%
Yes	7,933	1.24%	891	1.64%	8,824	1.27%
Depression PEDW (mother)						
No	634,990	99.35%	53,950	99.51%	688,940	99.36%
Yes	4,173	0.65%	264	0.49%	4,437	0.64%
Serious Mental Illness (mother)						
No	638,887	99.96%	54,185	99.95%	693,072	99.96%
Yes	276	0.04%	29	0.05%	305	0.04%
Anxiety GP (mother)						
No	629,681	98.52%	53,131	98.00%	682,812	98.48%
Yes	9,482	1.48%	1,083	2.00%	10,565	1.52%
Anxiety PEDW (mother)						
No	635,910	99.49%	53,967	99.54%	689,877	99.50%
Yes	3,253	0.51%	247	0.46%	3,500	0.50%
Anti-depressant medication (mother)						
No	639,019	99.98%	54,194	99.96%	693,213	99.98%
Yes	144	0.02%	20	0.04%	164	0.02%
Vitamin D (mother)						
No	637,171	99.69%	54,170	99.92%	691,341	99.71%
Yes	1,992	0.31%	44	0.08%	2,036	0.29%
FOLIC Acid (mother)						
No	486,360	76.09%	37,663	69.47%	524,023	75.58%
Yes	152,803	23.91%	16,551	30.53%	169,354	24.42%

Anaemia GP (mother)						
No	621,276	97.20%	52,636	97.09%	673,912	97.19%
Yes	17,887	2.80%	1,578	2.91%	19,465	2.81%
Anaemia PEDW (mother)						
No	631,370	98.78%	53,883	99.39%	685,253	98.83%
Yes	7,793	1.22%	331	0.61%	8,124	1.17%
Alcohol - GP (mother)						
No	601,660	94.13%	50,836	93.77%	652,496	94.10%
Yes	37,503	5.87%	3,378	6.23%	40,881	5.90%
Alcohol - PEDW (mother)						
No	638532	99.90%	54177	99.93%	692,709	99.90%
Yes	631	0.10%	37	0.07%	668	0.10%
Assault - PEDW (mother)						
No	638,479	99.89%	54165	99.91%	692,644	99.89%
Yes	684	0.11%	49	0.09%	733	0.11%
Substance misuse – any (mother)						
No	607,433	95.04%	51,087	94.23%	658,520	94.97%
Yes	31,730	4.96%	3,127	5.77%	34,857	5.03%
Substance misuse - alcohol (mother)						
No	631,148	98.75%	53,235	98.19%	684,383	98.70%
Yes	7,767	1.22%	975	1.80%	8,742	1.26%
Unknown/NULL	248	0.04%	<5	-	-	-
Substance misuse - other (mother)						
No	632,443	98.95%	53,381	98.46%	685,824	98.91%
Yes	6,199	0.97%	794	1.46%	6,993	1.01%
Unknown/NULL	521	0.08%	39	0.07%	560	0.08%
Mother weight (kg)						
Average (before imputation)	71		72.39		71.06	
Median (before imputation)					67.58	
Average (after imputation)					70.82	
Median (after imputation)					67.00	
Living area						
Town and Fringe - Less Sparse	82,435	12.90%	12,702	23.43%	95,137	13.72%
Town and Fringe - Sparse	22,106	3.46%	71	0.13%	22,177	3.20%
Urban > 10K - Less Sparse	403,591	63.14%	37,924	69.95%	441,515	63.68%
Urban > 10K - Sparse	13,441	2.10%	37	0.07%	13,478	1.94%
Village, Hamlet & Isolated Dwellings - Less Sparse	46,166	7.22%	1,464	2.70%	47,630	6.87%
Village, Hamlet & Isolated Dwellings - Sparse	48,314	7.56%	169	0.31%	48,483	6.99%
Unknown/NULL	23,110	3.62%	1,847	3.41%	24,957	3.60%
Local authority						
Blaenau Gwent					15,008	2.16%
Bridgend					28,018	4.04%
Caerphilly					40,418	5.83%

Cardiff					80,247	11.57%
Carmarthenshire					34,705	5.01%
Ceredigion					11,090	1.60%
Conwy					20,389	2.94%
Denbighshire					19,697	2.84%
Flintshire					32,471	4.68%
Gwynedd					23,249	3.35%
Isle of Anglesey					13,941	2.01%
Merthyr Tydfil					13,259	1.91%
Monmouthshire					14,899	2.15%
Neath Port Talbot					28,854	4.16%
Newport					35,153	5.07%
Pembrokeshire					23,929	3.45%
Powys					20,546	2.96%
Rhondda Cynon Taff					54,214	7.82%
Swansea					49,588	7.15%
Torfaen					20,500	2.96%
Vale of Glamorgan					25,657	3.70%
Wrexham					29,346	4.23%
Unknown/NULL					58,199	8.39%

Supplementary Table 3: Multivariable logistic regression model to identify the risk factors of LBW among the overall study population.

Variable name in model (description)	OR	Lower CI	Upper CI
GENDER (Gender)			
Boy	1		
Girl	1.16	1.14	1.18
MOMSMOKE (Maternal smoking)			
No	1		
Yes	1.80	1.76	1.84
MOMAGE (Maternal age)			
Less than 19	0.94	0.90	0.97
20-24	1.00	0.97	1.03
25-29	1		
30-34	1.05	1.02	1.09
35 and above	1.24	1.20	1.29
BIRTHORDER (Birth order)			
1st child	1		
2nd child	0.59	0.57	0.60
3rd child	0.65	0.62	0.67
4th or above	0.84	0.80	0.88
PREGNANCY_INTERVAL (Pregnancy interval)			
Less than 1 year	2.92	2.70	3.15
1-2 years	1.13	1.09	1.18
2-5 years	1		
5-7 years	1.15	1.10	1.21
7-10 years	1.30	1.24	1.37
Above 10 years	1.60	1.51	1.70
MULTIPLE_BIRTH (Multiple birth flag)			
Singleton			
Non-singleton	21.74	21.09	22.40
WIMD (Welsh Index of Multiple Deprivation)			
1 (most deprived)	1		
2	0.91	0.88	0.94
3	0.84	0.82	0.87
4	0.78	0.75	0.81
5 (least deprived)	0.70	0.67	0.72
WIMDENV (Welsh Index of Multiple Deprivation – Environment score)			
1 (most deprived)	1		
2	0.99	0.96	1.02
3	1.01	0.98	1.04
4	1.02	0.99	1.06
5 (least deprived)	1.00	0.96	1.03
LA (Local authority)			
Blaenau Gwent	1.23	1.15	1.32

Bridgend	1.01	0.95	1.07
Caerphilly	1.06	1.00	1.11
Cardiff	1		
Carmarthenshire	1.02	0.96	1.08
Ceredigion	0.91	0.83	1.01
Conwy	1.05	0.98	1.12
Denbighshire	1.13	1.06	1.21
Flintshire	1.08	1.02	1.14
Gwynedd	1.04	0.97	1.12
Isle of Anglesey	1.11	1.02	1.20
Merthyr Tydfil	1.13	1.05	1.22
Monmouthshire	1.02	0.94	1.10
Neath Port Talbot	0.94	0.89	1.00
Newport	1.13	1.08	1.19
Pembrokeshire	1.04	0.97	1.11
Powys	1.01	0.93	1.09
Rhondda Cynon Taff	1.23	1.17	1.28
Swansea	0.97	0.93	1.02
Torfaen	1.09	1.02	1.16
Vale of Glamorgan	0.98	0.92	1.04
Wrexham	1.20	1.14	1.27
MOM_DIAB_GP (Diabetes GP (mother))			
No	1		
Yes	2.03	1.81	2.28
MOM_DIAB_PEDW (Diabetes PEDW (mother))			
No	1		
Yes	1.32	1.01	1.74
MOM_DEPRE_GP (Depression GP (mother))			
No	1		
Yes	1.24	1.14	1.34
MOM_DEPRE_PEDW (Depression PEDW (mother))			
No	1		
Yes	1.58	1.43	1.75
MOM_SeriousMentalillness_GP (Serious Mental Illness (mother))			
No	1		
Yes	1.46	1.04	2.05
MOM_VITD_GP (Vitamin D (mother))			
No	1		
Yes	1.15	0.96	1.38
MOM_FOLIC_GP			
No	1		
Yes	1.09	1.06	1.11
MOM_ALCO_GP (Alcohol - GP (mother))			
No	1		

Yes	1.02	0.98	1.06
MOM_ALCO_PEDW (Alcohol -PEDW (mother))			
No	1		
Yes	1.60	1.30	1.97
MOM_ANXIETY_GP (Anxiety GP (mother))			
No	1		
Yes	1.10	1.02	1.19
MOM_ANXIETY_PEDW (Anxiety PEDW (mother))			
No			
Yes	1.22	1.08	1.38
MOM_ANTIDEP_MED (Anti-depressant medication (mother))			
No	1		
Yes	1.92	1.20	3.07
MOM_ANAEMIA_GP (Anaemia GP (mother))			
No	1		
Yes	0.70	0.65	0.74
MOM_ANAEMIA_PEDW (Anaemia PEDW (mother))			
No	1		
Yes	1.26	1.16	1.36
MOM_ASSAULT (Assault - PEDW (mother))			
No	1		
Yes	1.16	0.91	1.47
MOM_SubstanceMisuse_Any (Substance misuse – any (mother))			
No	1		
Yes	1.35	1.29	1.41
MOM_SubstanceMisuse_Alcohol (Substance misuse - alcohol (mother))			
No	1		
Yes	1.27	1.17	1.38
MOM_SubstanceMisuse_Otherdrug (Substance misuse - other (mother))			
No	1		
Yes	1.14	1.04	1.24
MOMWEIGHT (Mother weight)			
	0.99	0.99	0.99
LIVINGAREA (Living area)			
Town and Fringe - Less Sparse	1.02	0.95	1.09
Town and Fringe - Sparse	1.03	0.96	1.12
Urban > 10K - Less Sparse	1		
Urban - Sparse	1.02	0.99	1.05
Village, Hamlet & Isolated Dwellings - Less Sparse	0.95	0.91	0.99
Village, Hamlet & Isolated Dwellings - Sparse	0.89	0.85	0.94

Supplementary Table 4: Distribution of LBW and nLBW children based on their multiple birth flags

	Overall training set (n = 639,163)		Overall test set (n = 54,214)		Total (n = 693,377)	
Singleton						
nLBW	585,163	94.46%	49,145	93.46%	634,308	94.39%
LBW	34,295	5.54%	3,438	6.54%	37,733	5.61%
Non-singleton						
nLBW	9,245	46.92%	589	36.11%	9,834	46.09%
LBW	10,460	53.08%	1,042	63.89%	11,502	53.91%
