# Supplementary material for: Weighting of risk factors for low birth weight: a linked routine data cohort study in Wales, UK
Source: BMJ Open. 2023 Feb 10;13(2):e063836. doi: 10.1136/bmjopen-2022-063836 (PMC9923297; doi:10.1136/bmjopen-2022-063836)
Supplement: Supplementary data [file bmjopen-2022-063836supp002.pdf]

**Supplementary Figure 1: Significant risk factors associated with the risk LBW after linking with PPN record**

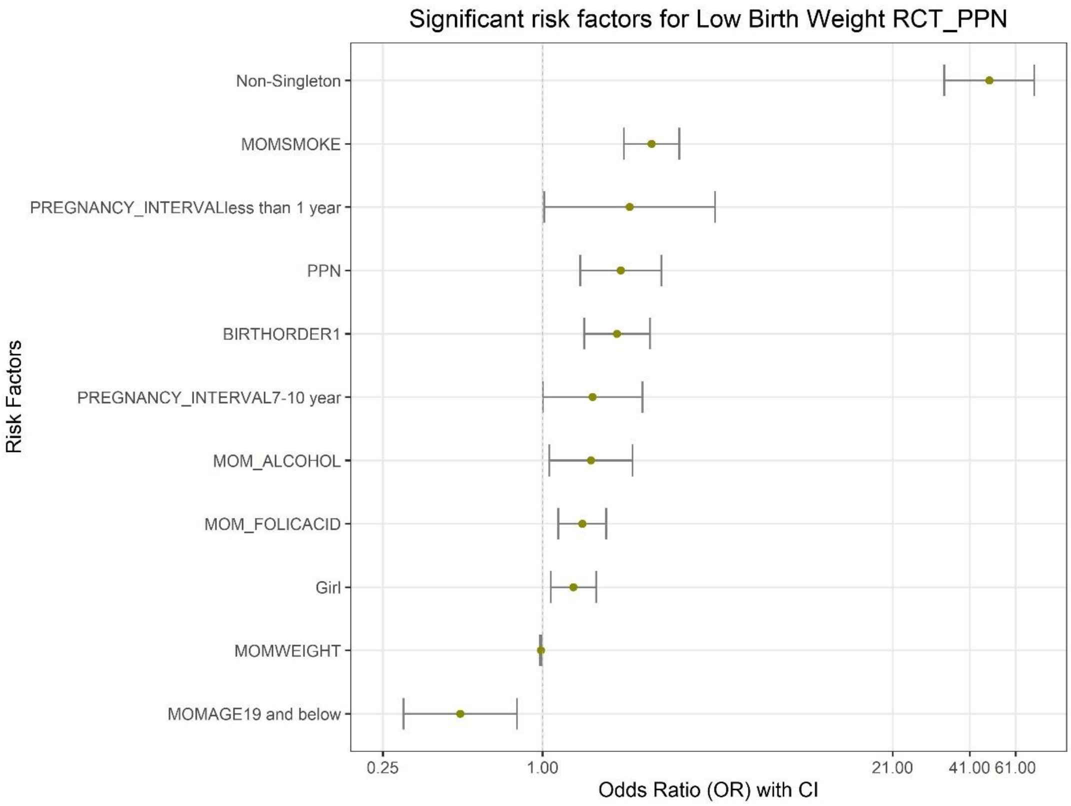

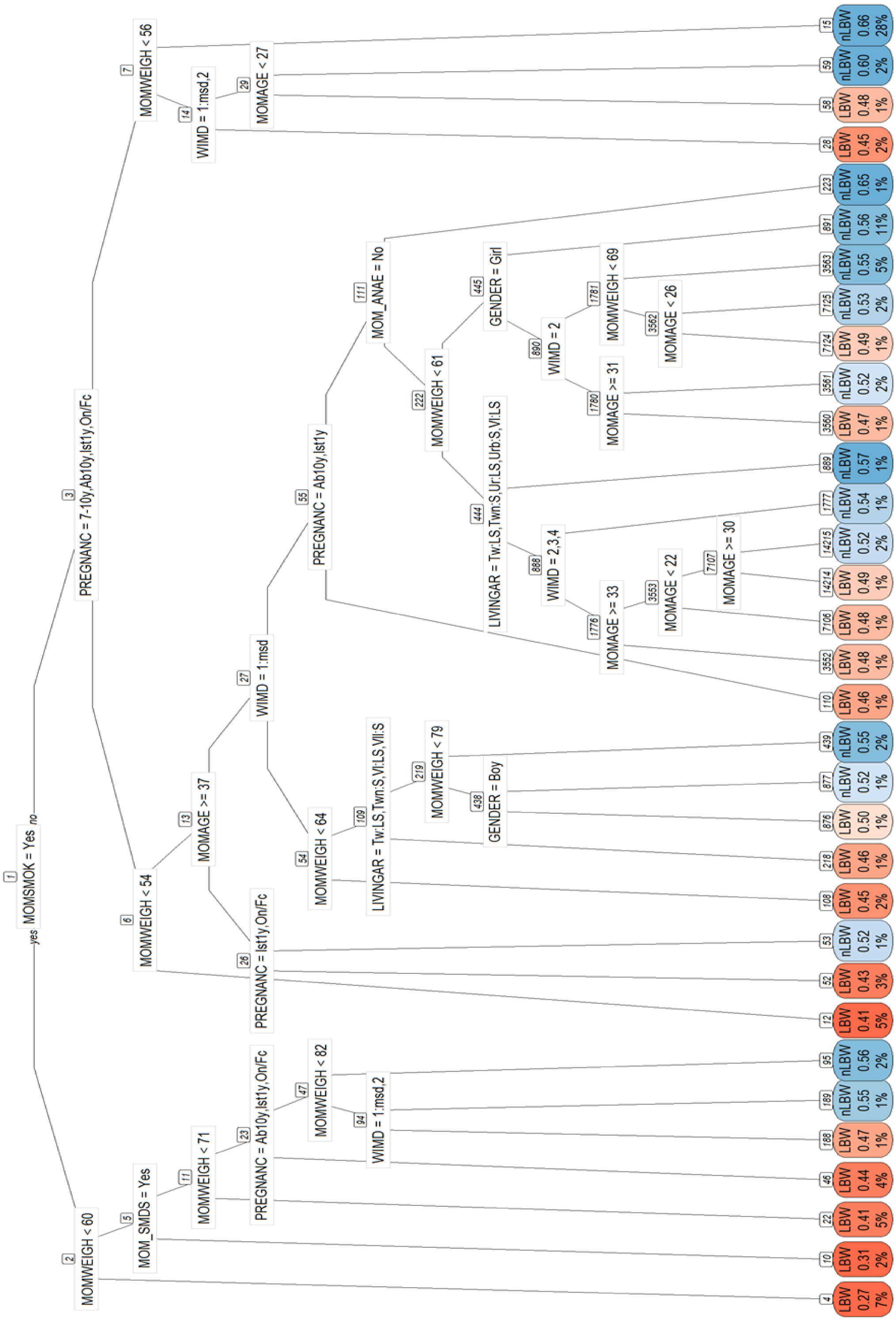


**Supplementary Figure 2: Decision tree for singleton children**

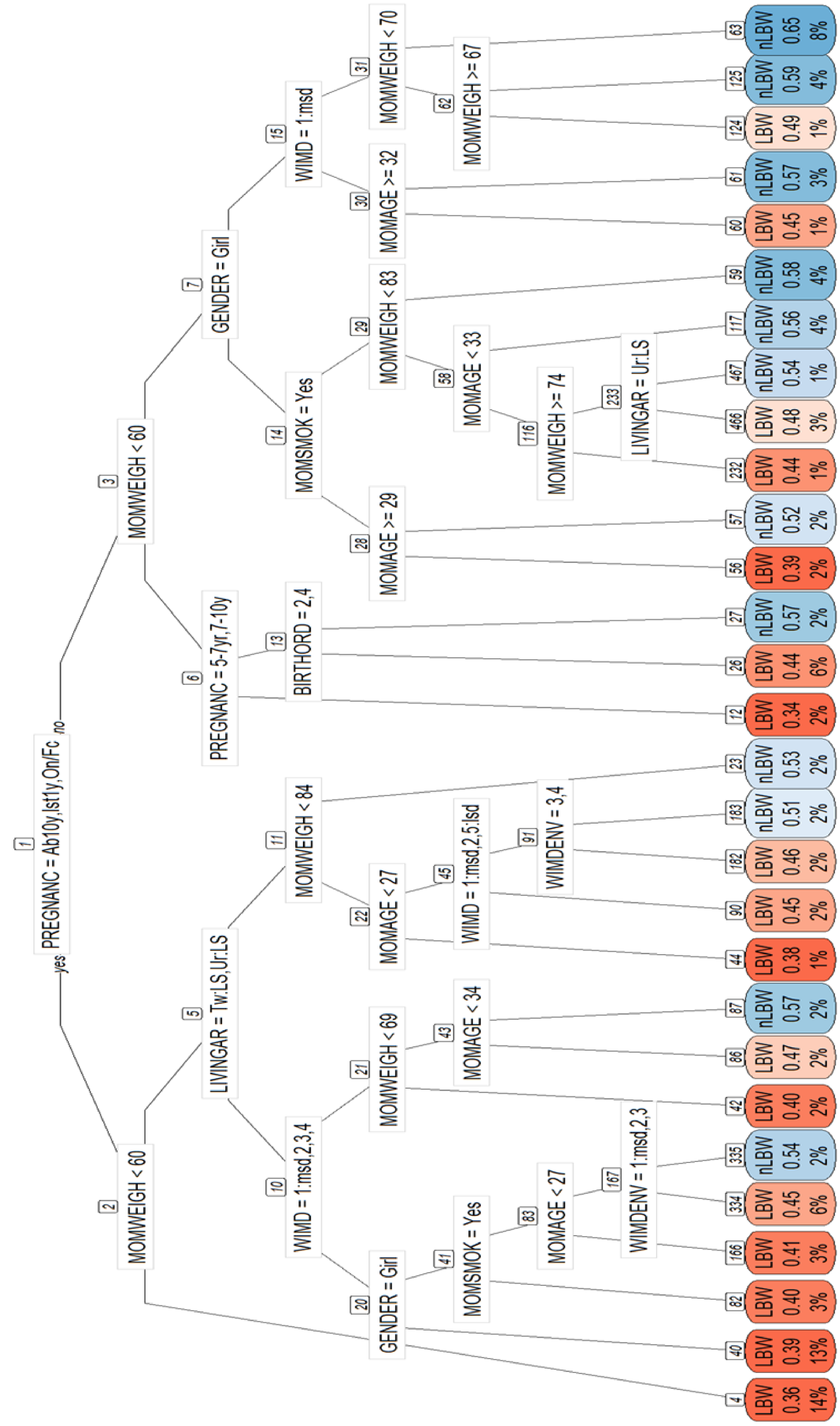


**Supplementary Figure 3: Decision tree for non-singleton children**
