# Supplementary material for: Weighting of risk factors for low birth weight: a linked routine data cohort study in Wales, UK
Source: BMJ Open. 2023 Feb 10;13(2):e063836. doi: 10.1136/bmjopen-2022-063836 (PMC9923297; doi:10.1136/bmjopen-2022-063836)
Supplement: Author's manuscript [file bmjopen-2022-063836.draft_revisions.pdf]

BMJ Open

BMJ Open is committed to open peer review. As part of this commitment we make the peer review history of every article we publish publicly available.

When an article is published we post the peer reviewers' comments and the authors' responses online. We also post the versions of the paper that were used during peer review. These are the versions that the peer review comments apply to.

The versions of the paper that follow are the versions that were submitted during the peer review process. They are not the versions of record or the final published versions. They should not be cited or distributed as the published version of this manuscript.

BMJ Open is an open access journal and the full, final, typeset and author-corrected version of record of the manuscript is available on our site with no access controls, subscription charges or pay-per-view fees (http://bmjopen.bmj.com).

If you have any questions on BMJ Open's open peer review process please email info.bmjopen@bmj.com

BMJ Open

Weighting of risk factors for low birth weight: A linked routine data cohort study in Wales, UK.

Journal:	*BMJ Open*
Manuscript ID	bmjopen-2022-063836
Article Type:	Original research
Date Submitted by the Author:	19-Apr-2022

Complete List of Authors:	Bandyopadhyay, Amrita; Swansea University, National Centre for Population Health and Wellbeing Research<br>Jones, Hope; Swansea University, National Centre for Population Health and Wellbeing Research<br>Parker, Michael; Swansea University, National Centre for Population Health and Wellbeing Research<br>Marchant, Emily ; Swansea University, National Centre for Population Health and Wellbeing Research<br>Evans, Julie; Public Health Wales, Keir Hardie University Health Park<br>Todd, Charlotte; Public Health Wales, Keir Hardie University Health Park<br>Rahman, Muhammad A.; Cardiff Metropolitan University, Cardiff School of Technologies<br>Healy, James; Swansea University, National Centre for Population Health and Wellbeing Research; Office for National Statistics<br>Win, Tint ; Swansea University, National Centre for Population Health and Wellbeing Research<br>Rowe, Ben; National Police Chiefs' Council<br>Moore, Simon; Cardiff University, School of Dentistry; Cardiff University, Security, Crime, Intelligence Institute<br>Jones, Angela; Public Health Wales, Keir Hardie University Health Park<br>Brophy, Sinead; Swansea University, National Centre for Population Health and Wellbeing

Keywords:	EPIDEMIOLOGY, PUBLIC HEALTH, STATISTICS & RESEARCH METHODS

SCHOLARONE™
Manuscripts

I, the Submitting Author has the right to grant and does grant on behalf of all authors of the Work (as defined in the below author licence), an exclusive licence and/or a non-exclusive licence for contributions from authors who are: i) UK Crown employees; ii) where BMJ has agreed a CC-BY licence shall apply, and/or iii) in accordance with the terms applicable for US Federal Government officers or employees acting as part of their official duties; on a worldwide, perpetual, irrevocable, royalty-free basis to BMJ Publishing Group Ltd ("BMJ") its licensees and where the relevant Journal is co-owned by BMJ to the co-owners of the Journal, to publish the Work in this journal and any other BMJ products and to exploit all rights, as set out in our licence.

The Submitting Author accepts and understands that any supply made under these terms is made by BMJ to the Submitting Author unless you are acting as an employee on behalf of your employer or a postgraduate student of an affiliated institution which is paying any applicable article publishing charge ("APC") for Open Access articles. Where the Submitting Author wishes to make the Work available on an Open Access basis (and intends to pay the relevant APC), the terms of reuse of such Open Access shall be governed by a Creative Commons licence – details of these licences and which Creative Commons licence will apply to this Work are set out in our licence referred to above.

Other than as permitted in any relevant BMJ Author's Self Archiving Policies, I confirm this Work has not been accepted for publication elsewhere, is not being considered for publication elsewhere and does not duplicate material already published. I confirm all authors consent to publication of this Work and authorise the granting of this licence.

For peer review only

Weighting of risk factors for low birth weight: A linked routine data cohort study in Wales, UK.

Amrita Bandyopadhyay[1*], Hope Jones[1], Michael Parker[1], Emily Marchant[1], Julie Evans[2], Charlotte Todd[2], Muhammad A. Rahman[3], James Healy [1,4], Tint Lwin Win[1], Ben Rowe[5], Simon Moore[6,7], Angela Jones[2], Sinead Brophy[1].

1 National Centre for Population Health and Wellbeing Research, Swansea University Medical School, Wales, SA2 8PP, UK
2 Public Health Wales, Keir Hardie University Health Park, CF48 1BZ, Wales. UK
3 Cardiff School of Technologies, Cardiff Metropolitan University, Llandaff Campus, CF5 2YB, Wales, UK
4 Office for National Statistics, Government Buildings, Cardiff Road, Duffryn, Newport, NP10 8XG, UK
5 National Police Chiefs' Council Lead for Mental Health and Age, 10 Victoria Street, London, SW1H 0NN, UK
6 School of Dentistry, Cardiff University, Cardiff, Wales, CF14 4XY, UK
7 Security, Crime, Intelligence Institute, Cardiff University, SPARK, Maindy Road, Cardiff, Wales, CF10 3AE, UK

* Corresponding author

Name of the Corresponding Author: Amrita Bandyopadhyay

Affiliations: National Centre for Population Health and Wellbeing Research, Swansea University Medical School, Wales, SA2 8PP, UK

Email Address: a.bandyopadhyay@swansea.ac.uk

List of keywords: Low birth weight, Maternal health, Pregnancy interval, Data linkage, Cohort study

For peer review only

Abstract

Objective

Globally 20 million children are born with a birth weight below 2,500 grams every year which is considered as a low birthweight (LBW) baby. This study investigates the contribution of modifiable risk factors in a nationally representative Welsh e-cohort of the children and the mothers to inform activities that reduce the rates of LBW.

Design

A longitudinal cohort study based on anonymously-linked, routinely-collected multiple administrative datasets.

Participants

The cohort, (N=693,377) comprising of children born between 1st January 1998 and 31st December 2018 in Wales, was selected from the National Community Child Health database (NCCHD).

Outcome measures

The risk factors associated with a binary LBW (outcome) variable were investigated with multivariable logistic regression and predictive machine learning-based decision tree models.

Results

The study found that non-singleton children had the highest risk of LBW (adjusted odds ratio 21.74 (95% confidence interval (CI) 21.09–22.40)), followed by pregnancy interval less than one year (2.92, 95% CI 2.70–3.15), maternal diabetes (2.03, 95% CI 1.81–2.28), maternal hospital admission for anaemia (1.26, 95% CI 1.16–1.36), depression (1.58, 95% CI 1.43–1.75), serious mental illness (1.46, 95% CI 1.04–2.05), anxiety (1.22, 95% CI 1.08–1.38) and use of anti-depressant medication during pregnancy (1.92, 95% CI 1.20–3.07). Additional maternal risk factors include smoking (1.80, 95% CI 1.76–1.84), alcohol-related hospital admission (1.60, 95% CI 1.30–1.97), substance misuse (1.35, 95% CI 1.29–1.41) and evidence of domestic abuse (1.98, 95% CI 1.39–2.81), living in less deprived area has lower risk of LBW (0.70, 95% CI 0.67–0.72).

For peer review only - http://bmjopen.bmj.com/site/about/guidelines.xhtml

Conclusion

The findings highlight that, measures to reduce the prevalence of LBW, need to focus on the principal risk factors of maternal health, pre-term births, awareness of sufficient pregnancy interval, adequate support and resources for mother's mental health and wellbeing.

For peer review only

For peer review only - http://bmjopen.bmj.com/site/about/guidelines.xhtml

Strengths and limitations of this study

- This study has built an e-cohort using data-linkage across multiple routinely collected administrative datasets to investigate the risk factors of LBW for the population of Wales.
- The study has investigated the modifiable risk factors of LBW in a holistic framework by linking primary and secondary care physical and mental health, socio- demographic and pregnancy related routine data including police record for a nationally representative sample.
- This study undertook two different statistical approaches (regression analysis and data-driven machine learning algorithm) which is a real strength of the study. This allowed to investigate the factors which are common and so are predictive (using data-driven machine learning algorithm) and also investigated the factors which have a strong association with LBW, but probably not so common in the population (using regression analysis).
- This finding highlighted the adequate support needed to the pregnant women during pregnancy and a multiagency (doctors, midwives, security department) level intervention can initiate the necessary mitigative steps required to reduce the prevalence of LBW.
- This work were unable to include any important risk factors which were not recorded in the health care system or any conditions which were undiagnosed hence that did not result in the system.

For peer review only

For peer review only - http://bmjopen.bmj.com/site/about/guidelines.xhtml

Introduction

The World Health Organisation (WHO) defines low birth weight (LBW) as infants weighting less than 2,500 grams (5.5 pounds) irrespective of gestational age [1,2]. Latest figures show that each year around 53,000 live births (6.9%) are identified as LBW in the UK [3]. LBW is the result of intra-uterine growth restriction (less than 10th centile of weight for sex and gestational age), prematurity (gestational age less than 37 weeks), or a combination of both [4]. LBW can impair the baby's cognitive development and lead to developmental disabilities and poor academic achievement [5]. Furthermore, LBW significantly increases the risk of perinatal and neonatal mortality and longstanding morbidity in early and later life [6]. Whilst there has been a reduction in mortality amongst preterm infants in the last two decades, the incidence of preterm birth has increased in many developed countries [6–8]. The increase is also associated with preterm delivery of multiple pregnancies, with medically indicated preterm birth 10 times more likely in multiple pregnancies than singleton births [9]. To address the global burden of LBW, the Sixty Fifth World Health Assembly Resolution 65.6 endorsed a comprehensive implementation plan to achieve a 30% reduction in LBW by 2025 [1]. A study conducted on the birth data from 148 countries of 195 United Nations' member states indicated that there had been a 2.9% reduction in the LBW prevalence in 2015, compared to 2000 worldwide. However, there has not been any change in the LBW prevalence in high income regions (including Europe) and the progress is slower than required to meet the WHO LBW target by 2025 [10].

Existing research has found factors linked with mothers, such as age, high deprivation, and low academic qualification, are associated with increased odds of LBW [11,12]. Modifiable risk factors for LBW include inter-pregnancy interval [13], maternal physical [14–17] and mental health [18,19], and environmental exposures during pregnancy [20]. Studies have also shown numerous health behaviours, particularly smoking during pregnancy [21,22], alcohol, in which there is a dose-response relationship with LBW [24], and/or illicit drug use [23] are modifiable risk factors. Indirect (negative maternal behaviours, inadequate nutrition or prenatal care, and increased stress) or direct (physical assault, sexual trauma) experience of intimate partner abuse during pregnancy can lead to adverse infant outcomes, including LBW [25,26].

It is important to gain an understanding of these risk factors, particularly modifiable risk factors, so that resources and interventions can be scheduled effectively. Moreover, the wide range of risk factors cannot be addressed in isolation. Most of the risk factors that are strongly independently associated with LBW are correlated. This study aimed to understand the contributions of risk factors to the burden of LBW for the population of Wales, using traditional statistical methods and supervised machine learning models.

For peer review only - http://bmjopen.bmj.com/site/about/guidelines.xhtml

For peer review only

Method

Participants and linkage

The linked data cohort (N = 693,377) was comprised of children born in Wales between 1st January 1998 and 31st December 2018. The study population was identified in the National Community Child Health database (NCCHD), which is a local Child Health System database held by the National Health Service (NHS). The participants were linked to the Wales-wide administrative register, the Wales Demographic Service Dataset (WDS). Linkage was undertaken using an anonymised encrypted linkage key, the Anonymised Linking Field (ALF), in the Secure Anonymised Information Linkage (SAIL) Databank [27]. WDS provided the anonymised residential linking fields (RALFs), which is an encrypted residential address and its corresponding lower super output area (LSOA, small geographic areas with a population of approximately 1,500) when the child was born. LSOA was linked with the Welsh Index of Multiple Deprivation (WIMD) 2014, which is a measure of relative deprivation. The participants flow diagram is displayed in Figure 1.

Explanatory variables

The maternal variables related to a childbirth were obtained from NCCHD and Maternal Indicator Database (MID). The variables for maternal physical and mental health during pregnancy were obtained from primary care Welsh Longitudinal General Practice (WLGP) and hospital admissions dataset known as Patient Episode database in Wales (PEDW). The record of physical assault linked with mothers during pregnancy was obtained from PEDW. The substance misuse database (SMD) provided the information on alcohol and other drug abuse by the mother during pregnancy. Area type (urban/rural) and local authority (LA) under which they lived during the pregnancy and their overall and physical environment quantified in the WIMD were included in this study. The derived maternal variables include multiple birth flag (to distinguish between singleton and non-singleton), pregnancy interval, harmonised maternal smoking, and maternal weight. The variables and their sources have been described in Supplementary Table 1.

The impact of domestic abuse was examined using a subset of the study population (participants from Rhondda Cynon Taff born between June 2016 and 2018) with linked Public Protection Notification (PPN) dataset [28].

Outcome variable

In this study a binary variable was created using the birth weight variable obtained from NCCHD.

- LBW = birth weight <2,500

For peer review only - http://bmjopen.bmj.com/site/about/guidelines.xhtml

- Not LBW (nLBW) = birth weight ≥2,500

Statistical Analysis

It is known that gestational age is highly correlated with LBW. However, as the gestational age is only obtained at the point of birth, making it a non-modifiable risk factor, this study has not considered it as a predictor variable. The models were stratified by the multiple birth as this is one of the main predictors of LBW. The missing records in birth weight variable were removed from the analysis. Since there was around 15% missing data in maternal weight variable, the variable was imputed by the simple random imputation method [29]. The missing data in the other explanatory variables (less than 10%) were recoded as 'Unknown'. The birth record for stillbirth and pregnancy interval of less than 22 weeks (as that is the minimum duration for a considerable gestation period) were also not considered for the statistical analysis. Data preparation including data linkage and data cleaning for this analysis was done on SAIL DB2 SQL platform. All statistical analyses were performed in R version 4.0.3.

A multivariable logistic regression (MLR) model was developed to identify the most important risk factors associated with LBW. The MLR model was built on the overall study population (whole Wales dataset) to identify the associations between all the explanatory and outcome variables.

A supervised machined learning classifier - decision tree (DT) model was developed to build a risk profile for LBW and test its predictive performance. Classification tree – DT models were constructed using RPART (Recursive Partitioning And Regression Trees) packages in R [30,31]. The algorithm recursively partitions the data into multiple sub-spaces to obtain the homogeneous final sub-space of predictor variables. For DT, the whole Wales data except for Rhondda Cynon Taff was used to train the model and prediction performance was evaluated on a test dataset which consisted of a sample of participants from the LA of Rhondda Cynon Taff. This LA was chosen because it had one of the highest rates of LBW in Wales and is an area which would benefit most from an accurate prediction model.

A separate data linkage was undertaken with a subset of the study population which was linked to the mother's domestic abuse record from PPN dataset (the latter was only available for Rhonda Cynon Taff). Another adjusted MLR model was developed on this linked data to investigate the risk association for LBW.

Patient and Public Involvement

No patient involved.

For peer review only - http://bmjopen.bmj.com/site/about/guidelines.xhtml

For peer review only

Results

The study population consisted of 693,377 children of those 54,214 were from Rhondda Cynon Taff and 639,163 were from other LAs. The children from Rhondda Cynon Taff, which was later used as a test set for DT were well representative of the Welsh population (see Supplementary Table 2). In the overall study population, 51.26% were boys, 96.92% were singleton and 90.38% children were born to term (gestational age between 37 and 42 weeks). 49.85% children were born as the first child in the family. Mothers of 0.48% children were admitted to hospital for diabetes and 0.09% had a GP visit for diabetes, 1.27% had depression, 1.52% with anxiety and 0.02% were on antidepressant medication during pregnancy. There were 1.26% and 21.51% children whose mothers had alcohol-related substance misuse and smoking record during pregnancy respectively. The average maternal age at birth of child and maternal weight was 28 years and 70.82 kg (after imputation) respectively and 63.68% of them were living in densely populated urban areas. Overall, 7.1% (8.26% in test set and 7% in other LAs) of children were born as LBW.

Factors associated with LBW: MLR results

Non-singleton children were at almost 22 times higher risk of LBW than singleton children (adjusted odds ratio (aOR) – 21.74 (95% confidence interval (CI) 21.09 – 22.40)). Mothers with diabetes-related GP visits (2.03 (1.81 – 2.28)) and hospital admission records of anaemia (1.26 (1.16 – 1.36)) during pregnancy were at very high risk of having LBW children. Poor mental health during pregnancy such as severe depression (1.58 (1.43– 1.75)), serious mental illness (1.46 (1.04 – 2.05)), severe anxiety (1.22 (1.08 – 1.38)) and antidepressant medications (1.92 (1.20– 3.07)) were risk factors for LBW. The other highly significant modifiable risk factors linked with pregnant mothers include maternal smoking (1.80 (1.76 – 1.84)), alcohol related hospital admissions (1.60 (1.30 – 1.97)) and any substance misuse (alcohol/other drugs) (1.35 (1.29 – 1.41)) during pregnancy. Higher maternal age was also associated with the risk of LBW. Though maternal age less than 19 was significantly associated with the risk of LBW in the univariable model, after adjusting all the other explanatory variables, this did not remain as a risk factor of LBW. The first child born was at higher risk of LBW than subsequent births, The odds of LBW for the 2nd child was 0.59 (0.57 – 0.60) compared to the first child. Mothers living in the least deprived and rural areas during pregnancy were at lower risk of having LBW children than others living in more deprived and urban areas. The statistically significant risk factors with their aOR and CI have been visualised and described in Figure 2 and Supplementary Table 3.

Finding from the linked PPN data model

For peer review only - http://bmjopen.bmj.com/site/about/guidelines.xhtml

Linkage of the cohort with PPN gave a dataset of 5,854 mothers of those who had a PPN call during pregnancy 18% had a LBW child whereas those who did not have PPN call 8.7% had a child with LBW (see Table 1). Mothers with a PPN call during pregnancy had almost 2 times higher risk of having a LBW baby (1.98 (1.39 – 2.81)) than mothers without PPN call after adjusting for confounding factors (see Supplementary Figure 1).

Table 1: Distribution of LBW and nLBW children for the subset who were linked with mother's PPN record during pregnancy

PPN record during pregnancy	n = 5,854	
No		
nLBW	5,074	91.3%
LBW	485	8.7%
Yes		
nLBW	241	82%
LBW	53	18%

Predictive DT model

Since LBW were disproportionately more prevalent in non-singleton children (5.61% singleton vs 53.91% of the non-singleton children were LBW) (Supplementary Table 4) , two separate predictive models using DTs were developed.

Singleton children

There were 619,458 observations in the training model. The most important risk factors selected by the DT algorithm to develop the final tree were maternal smoking, maternal weight, pregnancy interval, birth order, maternal substance misuse record (any), maternal age, deprivation - WIMD score, maternal substance misuse record (other drug) and maternal substance misuse record (alcohol). Supplementary Figure 2 depicts the final tree with the branches including final 33 terminal nodes. For example, the model would predict a LBW baby if a) maternal smoking is positive (e.g., mum smokes during pregnancy) and b) maternal weight less than 60 kg. The number of women in this category who had a LBW child is 73% (see terminal node 4 in Supplementary Figure 2) and risk profile was found in 7% of the training model population (e.g., 7% of pregnant women were smokers who weighed less than 60 kg during pregnancy).

The test data was built on the 52,583 singleton children, which is 7.82% of the total singleton children in this study. The model performance is explained in a confusion matrix with 60.54% accuracy, 60.41% sensitivity, 60.55% specificity, 9.68% positive predictive values and 95.63% negative predictive value (see Table 2 and 3).

For peer review only - http://bmjopen.bmj.com/site/about/guidelines.xhtml

Table 2: Confusion matrix/two by two table of the DT (singleton and non-singleton) models

**Prediction**	**Reference** (Singleton) n = 52,583		**Reference** (Non-singleton) n = 1,631	
	LBW	nLBW	LBW	nLBW
LBW	2,077 (TP)	19,389 (FP)	716 (TP)	347 (FP)
nLBW	1,361 (FN)	29,756 (TN)	326 (FN)	242 (TN)

For peer review only

Table 3: Prediction model performance (n=52,583 Singleton, n = 1,631 Non-singleton from test set)

	**Accuracy**	**Sensitivity**	**Specificity**	**Positive Predictive Value**	**Negative Predictive Value**
**DT Singleton model**	60.54%	60.41%	60.55%	09.68%	95.63%
**DT Non-singleton model**	58.74%	68.71%	41.09%	67.36%	42.61%

Non-singleton children

There were 19,705 children in the non-singleton training subset. The variables selected to generate the tree by the DT algorithm in the importance order were pregnancy interval, birth order, maternal weight, maternal age, gender, deprivation - WIMD score, maternal smoking, living area, deprivation - WIMD (environment) score and maternal substance misuse record (any). Supplementary Figure 3 depicts the final tree with the branches including final 29 terminal nodes. For example, the model would predict a LBW baby if a) this is the first child or pregnancy interval is either above 10 years or less than 1 year and b) maternal weight less than 60 kg (terminal node 4).

The test set was built on the 1,631 non-singleton children, which is 7.64% of the total non-singleton children in this study. The model performance was measured as 58.74% accuracy, 68.71% sensitivity, 41.09% specificity, 67.36% positive predictive values and 42.61% negative predictive value (see Table 2 and 3).

Discussion

7.1% of the overall study population in Wales was LBW between 1998 and 2018. Global trend of LBW is around 7.0% in both 2000 and 2015 for the developed regions (Europe, North America, Australia),

For peer review only - http://bmjopen.bmj.com/site/about/guidelines.xhtml

which is consistent with our finding [2]. Findings from the Office for National Statistics (ONS) state a combined English and Welsh rate of LBW of 7.0% in 2016, unchanged from 2011 [32]. Our findings show that LBW is strongly associated with non-singleton pregnancy, and maternal health which includes a short pregnancy interval, non-optimal maternal body weight (e.g., low, or high weight), maternal smoking, diabetes, anaemia, mental illness and living in a deprived urban area and exposed to domestic abuse during pregnancy.

The findings of short and long pregnancy intervals being associated with increased odds of LBW has been reported previously [13]. However, Regan *et al.* highlighted many of the studies examining long inter-pregnancy interval are more prone to measurement error, with miscarriages and abortions in this time period difficult to capture and suggest caution should be taken interpreting these findings [33]. In terms of putting this evidence in context, when considering advice over pregnancy intervals, it will be important to consider all the available evidence including the impact of pregnancy interval on preterm birth and maternal outcomes [34]. Modifiable risk factors found to be important in this study included smoking during pregnancy. A number of reviews have been carried out in the field of interventions to reduce smoking in pregnancy which suggest that psychosocial interventions (counselling, feedback and incentives) appear to be effective at supporting women to stop smoking in pregnancy and can reduce the proportion of babies born LBW [35]. However, they argue that the context of the intervention needs to be given consideration and that whilst evidence exists for potentially effective interventions which could be piloted through delivery of programmes locally, efforts should also be directed at population wide strategies to reduce smoking uptake in young women. This may be especially important given the clear difficulties those pregnant experience in giving up smoking [35]. With regards to our finding of maternal mental health increasing the risk of LBW, both severe depression and anxiety were associated with an increased odds of LBW in our study [36].

The singleton DT model correctly predicted 60.41% of all the true positive cases. However, the positive predictive value of 9.68% indicates that the model assigned a false positive 'LBW' classification for 89.32% cases. This model only includes singleton children and since non-singleton pregnancies are highly associated with LBW, removing this variable from the model has lessened its predictive capability. This is evidenced by the significantly improved positive predictive value (67.36%) for the non-singleton model (table 3). Previous machine learning models appear to show better prediction as they included non-singleton, gestational age (which is in terms of temporal association highly associated with LBW but occurs at the same time as the LBW can be measured) and preeclampsia in third trimester.

For peer review only - http://bmjopen.bmj.com/site/about/guidelines.xhtml

For peer review only

This work can only identify the more severe cases which are recorded in the health care system, and undiagnosed cases that did not result in the system will be missed which is a limitation of this work. Since the study was developed on the linked routine data, the limitation of the routine data was encountered in this study, for example, though the maternal weight variable came from two different sources, for many participants the data were missing which were further addressed by imputation methods. The strength of this study lies in using national datasets of all births in Wales across a large time. However, as this was a linked routine data study some of the lifestyle factors cannot be captured (diet, physical activity, stress, emotional state) which can be important in determining LBW [37,38]. The two different models (MLR and DT) used in this study found very similar findings suggesting that factors which are common and so are predictive (using DT methods) such as maternal smoking status and low weight mother could be targeted to address population risk. Factors which have a strong association with LBW (using regression analysis) such as a mother with diabetes or mother on antidepressants, can be addressed to reduce individual risk for that mother/child.

Conclusion

This study suggests that the most important factors to reduce the risk of LBW are to address multiple birth (e.g. in assisted reproduction practices), addressing factors associated with preterm births (previous history of preterm birth), addressing maternal health such as reducing smoking, investment in maternal mental health, addressing substance use (alcohol/drugs), treating underlying health conditions (diabetes/anaemia), and promoting planning of pregnancy to give an adequate pregnancy interval and healthy weight of mother especially for those in deprived urban areas.

Acknowledgement

Funding

This work was funded by Public Health Wales (PHW), grant number (105186). This work was supported by Nation Institute for Health Research (NIHR), grant number (NIHR133680). This research has been carried out as part of the ADR Wales programme of work. The ADR Wales programme of work is aligned to the priority themes as identified in the Welsh Government's national strategy: Prosperity for All. ADR Wales brings together data science experts at Swansea University Medical School, staff from the Wales Institute of Social and Economic Research, Data and Methods (WISERD) at Cardiff University and specialist teams within the Welsh Government to develop new evidence which supports Prosperity for All by using the SAIL Databank at Swansea University, to link and analyse

For peer review only - http://bmjopen.bmj.com/site/about/guidelines.xhtml

anonymised data. ADR Wales is part of the Economic and Social Research Council (part of UK Research and Innovation) funded ADR UK (grant ES/S007393/1).

This work was also supported by the National Centre for Population Health and Well-Being Research (NCPHWR) which is funded by Health and Care Research Wales. This work was supported by Health Data Research UK which receives its funding from HDR UK Ltd (NIWA1) funded by the UK Medical Research Council, Engineering and Physical Sciences Research Council, Economic and Social Research Council, Department of Health and Social Care (England), Chief Scientist Office of the Scottish Government Health and Social Care Directorates, Health and Social Care Research and Development Division (Welsh Government), Public Health Agency (Northern Ireland), British Heart Foundation (BHF) and the Welcome Trust.

This work uses data provided by patients and collected by the NHS as part of their care and support. This study used anonymised data held in the Secure Anonymised Information Linkage (SAIL) Databank. We would like to acknowledge all the data providers who enable SAIL to make anonymised data available for research. We acknowledge the support provided by South Wales Police.

Contributorship statement

All authors contributed to the study conception and design. The police PPN and MID data accusation was supported by Ben Rowe and Julie Evans respectively. The core dataset was prepared by Muhammad A. Rahman. Further data preparation and the full analysis was done by Amrita Bandyopadhyay. James Healy, and Michael Parker contributed to the analysis. The first draft of the manuscript was written by Amrita Bandyopadhyay and all authors commented on previous versions of the manuscript. All authors read and approved the final manuscript.

Conceptualization: Sinead Brophy, Angela Jones and Julie Evans and Amrita Bandyopadhyay; Methodology: Amrita Bandyopadhyay and Sinead Brophy; Formal analysis and investigation: Amrita Bandyopadhyay Writing - original draft preparation: Amrita Bandyopadhyay; Writing - review and editing: Charlotte Todd, Michael Parker, Julie Evans, Emily Marchant, Hope Jones, Muhammad A. Rahman, James Healy, Tint Lwin Win, Ben Rowe, Simon Moore, Angela Jones, and Sinead Brophy, Supervision: Sinead Brophy.

Competing interest's statement

The authors declare that they have no conflict of interest.

For peer review only - http://bmjopen.bmj.com/site/about/guidelines.xhtml

The views expressed in this paper are those of the authors and not necessarily those of the Office for National Statistics

Participant consent

The study did not require participant consent as it utilises the anonymised data.

Ethical approval

No human participants were included.

The original protocol

Not applicable

STROBE checklist

STROBE checklist has been added as a Supplementary file (Supplementary material STROBE checklist).

Data sharing statement

The data have been archived in the Secure Anonymised Information Linkage Databank (https://saildatabank.com/0029)

References

1 WHO | Global Nutrition Targets 2025: Low birth weight policy brief. WHO. http://www.who.int/nutrition/publications/globaltargets2025_policybrief_lbw/en/ (accessed 19 Nov 2020).

2 UNICEF-WHO Low birthweight estimates: Levels and trends 2000–2015. https://www.unicef.org/reports/UNICEF-WHO-low-birthweight-estimates-2019 (accessed 3 Mar 2022).

3 Johnson CD, Jones S, Paranjothy S. Reducing low birth weight: prioritizing action to address modifiable risk factors. *J Public Health Oxf Engl* 2017;**39**:122–31. doi:10.1093/pubmed/fdv212

4 Mohammed SG. Low birth weight in Omdurman maternity hospital. *Int J Sci Res Publ* 2014;**4**:1–13.

5 Breslau N, Paneth NS, Lucia VC. The Lingering Academic Deficits of Low Birth Weight Children. *Pediatrics* 2004;**114**:1035–40. doi:10.1542/peds.2004-0069

6 Ohlsson A, Shah P. *Determinants and prevention of low birth weight: a synopsis of the evidence*. Institute of Health Economics 2008.

For peer review only - http://bmjopen.bmj.com/site/about/guidelines.xhtml

For peer review only

7 Heaman MI, Sprague AE, Stewart PJ. Reducing the Preterm Birth Rate: A Population Health Strategy. *J Obstet Gynecol Neonatal Nurs* 2001;**30**:20–9. doi:https://doi.org/10.1111/j.1552-6909.2001.tb01518.x

8 Yuan W, Duffner AM, Chen L, *et al.* Analysis of preterm deliveries below 35 weeks' gestation in a tertiary referral hospital in the UK. A case-control survey. *BMC Res Notes* 2010;**3**:119. doi:10.1186/1756-0500-3-119

9 Blencowe H, Cousens S, Chou D, *et al.* Born Too Soon: The global epidemiology of 15 million preterm births. *Reprod Health* 2013;**10**:S2. doi:10.1186/1742-4755-10-S1-S2

10 Blencowe H, Krasevec J, Onis M de, *et al.* National, regional, and worldwide estimates of low birthweight in 2015, with trends from 2000: a systematic analysis. *Lancet Glob Health* 2019;**7**:e849–60. doi:10.1016/S2214-109X(18)30565-5

11 Shi L, Macinko J, Starfield B, *et al.* Primary care, infant mortality, and low birth weight in the states of the USA. *J Epidemiol Community Health* 2004;**58**:374–80. doi:10.1136/jech.2003.013078

12 Silvestrin S, Silva CH da, Hirakata VN, *et al.* Maternal education level and low birth weight: a meta-analysis. *J Pediatr (Rio J)* 2013;**89**:339–45. doi:10.1016/j.jped.2013.01.003

13 Conde-Agudelo A, Rosas-Bermúdez A, Kafury-Goeta AC. Birth Spacing and Risk of Adverse Perinatal Outcomes: A Meta-analysis. *JAMA* 2006;**295**:1809–23. doi:10.1001/jama.295.15.1809

14 Yu Z, Han S, Zhu J, *et al.* Pre-Pregnancy Body Mass Index in Relation to Infant Birth Weight and Offspring Overweight/Obesity: A Systematic Review and Meta-Analysis. *PLOS ONE* 2013;**8**:e61627. doi:10.1371/journal.pone.0061627

15 Daalderop LA, Wieland BV, Tomsin K, *et al.* Periodontal Disease and Pregnancy Outcomes: Overview of Systematic Reviews. *JDR Clin Transl Res* 2018;**3**:10–27. doi:10.1177/2380084417731097

16 Flynn CA, Helwig AL, Meurer LN. Bacterial vaginosis in pregnancy and the risk of prematurity. *J Fam Pract* 1999;**48**:885–92.

17 Figueiredo ACMG, Gomes-Filho IS, Silva RB, *et al.* Maternal Anemia and Low Birth Weight: A Systematic Review and Meta-Analysis. *Nutrients* 2018;**10**. doi:10.3390/nu10050601

18 Dadi AF, Miller ER, Bisetegn TA, *et al.* Global burden of antenatal depression and its association with adverse birth outcomes: an umbrella review. *BMC Public Health* 2020;**20**:173. doi:10.1186/s12889-020-8293-9

19 Lima SAM, Dib RPE, Rodrigues MRK, *et al.* Is the risk of low birth weight or preterm labor greater when maternal stress is experienced during pregnancy? A systematic review and meta-analysis of cohort studies. *PLOS ONE* 2018;**13**:e0200594. doi:10.1371/journal.pone.0200594

20 Fleischer Nancy L., Merialdi Mario, van Donkelaar Aaron, *et al.* Outdoor Air Pollution, Preterm Birth, and Low Birth Weight: Analysis of the World Health Organization Global Survey on Maternal and Perinatal Health. *Environ Health Perspect* 2014;**122**:425–30. doi:10.1289/ehp.1306837

For peer review only - http://bmjopen.bmj.com/site/about/guidelines.xhtml

21 Flower A, Shawe J, Stephenson J, *et al.* Pregnancy planning, smoking behaviour during pregnancy, and neonatal outcome: UK millennium cohort study. *BMC Pregnancy Childbirth* 2013;**13**:238. doi:10.1186/1471-2393-13-238

22 Jaddoe VWV, Troe E-JWM, Hofman A, *et al.* Active and passive maternal smoking during pregnancy and the risks of low birthweight and preterm birth: the Generation R Study. *Paediatr Perinat Epidemiol* 2008;**22**:162–71. doi:10.1111/j.1365-3016.2007.00916.x

23 dos Santos JF, de Melo Bastos Cavalcante C, Barbosa FT, *et al.* Maternal, fetal and neonatal consequences associated with the use of crack cocaine during the gestational period: a systematic review and meta-analysis. *Arch Gynecol Obstet* 2018;**298**:487–503. doi:10.1007/s00404-018-4833-2

24 Patra J, Bakker R, Irving H, *et al.* Dose–response relationship between alcohol consumption before and during pregnancy and the risks of low birthweight, preterm birth and small for gestational age (SGA)—a systematic review and meta-analyses. *BJOG Int J Obstet Gynaecol* 2011;**118**:1411–21. doi:10.1111/j.1471-0528.2011.03050.x

25 Hill A, Pallitto C, McCleary-Sills J, *et al.* A systematic review and meta-analysis of intimate partner violence during pregnancy and selected birth outcomes. *Int J Gynaecol Obstet Off Organ Int Fed Gynaecol Obstet* 2016;**133**:269–76. doi:10.1016/j.ijgo.2015.10.023

26 Donovan BM, Spracklen CN, Schweizer ML, *et al.* Intimate partner violence during pregnancy and the risk for adverse infant outcomes: a systematic review and meta-analysis. *BJOG Int J Obstet Gynaecol* 2016;**123**:1289–99. doi:https://doi.org/10.1111/1471-0528.13928

27 Lyons RA, Jones KH, John G, *et al.* The SAIL databank: linking multiple health and social care datasets. *BMC Med Inform Decis Mak* 2009;**9**:3. doi:10.1186/1472-6947-9-3

28 Constabulary © Her Majesty's Inspectorate of, Fire. Police effectiveness 2015 (Vulnerability) – Dyfed-Powys Police. HMICFRS. https://www.justiceinspectorates.gov.uk/hmicfrs/publications/police-effectiveness-vulnerability-2015-dyfed-powys/ (accessed 3 Nov 2021).

29 Gelman A, Hill J. *Data analysis using regression and multilevel/hierarchical models*. Cambridge university press 2006.

30 Lewis RJ. An introduction to classification and regression tree (CART) analysis. In: *Annual meeting of the society for academic emergency medicine in San Francisco, California*. 2000.

31 Atkinson Beth. rpart function | R Documentation. https://www.rdocumentation.org/packages/rpart/versions/4.1-15/topics/rpart (accessed 14 Jan 2021).

32 ONS. Birth characteristics in England and Wales - Office for National Statistics. 2019.https://www.ons.gov.uk/peoplepopulationandcommunity/birthsdeathsandmarriages/livebirths/bulletins/birthcharacteristicsinenglandandwales/2017 (accessed 14 Jul 2021).

33 Regan AK, Ball SJ, Warren JL, *et al.* A Population-Based Matched-Sibling Analysis Estimating the Associations Between First Interpregnancy Interval and Birth Outcomes. *Am J Epidemiol* 2019;**188**:9–16. doi:10.1093/aje/kwy188

For peer review only - http://bmjopen.bmj.com/site/about/guidelines.xhtml

34 Hutcheon JA, Nelson HD, Stidd R, *et al.* Short interpregnancy intervals and adverse maternal outcomes in high-resource settings: An updated systematic review. *Paediatr Perinat Epidemiol* 2019;**33**:O48–59. doi:10.1111/ppe.12518

35 Chamberlain C, O'Mara-Eves A, Porter J, *et al.* Psychosocial interventions for supporting women to stop smoking in pregnancy. *Cochrane Database Syst Rev* Published Online First: 2017. doi:10.1002/14651858.CD001055.pub5

36 Howard LM, Khalifeh H. Perinatal mental health: a review of progress and challenges. *World Psychiatry* 2020;**19**:313–27. doi:10.1002/wps.20769

37 Ghavi A, Fadakar Sogheh K, Niknamy M, *et al.* Investigating the Relationship between Maternal Lifestyle during Pregnancy and Low-Birth-Weight of Term Neonates. *Iran J Obstet Gynecol Infertil* 2012;**15**:14–24.

38 Xi C, Luo M, Wang T, *et al.* Association between maternal lifestyle factors and low birth weight in preterm and term births: a case-control study. *Reprod Health* 2020;**17**:93. doi:10.1186/s12978-020-00932-9

For peer review only

Figure Legend

Figure 1 Participants flow diagram.

Figure 2: Significant factors associated with the risk LBW among the overall study population.

For peer review only - http://bmjopen.bmj.com/site/about/guidelines.xhtml

Figure 1 Participants flow diagram.

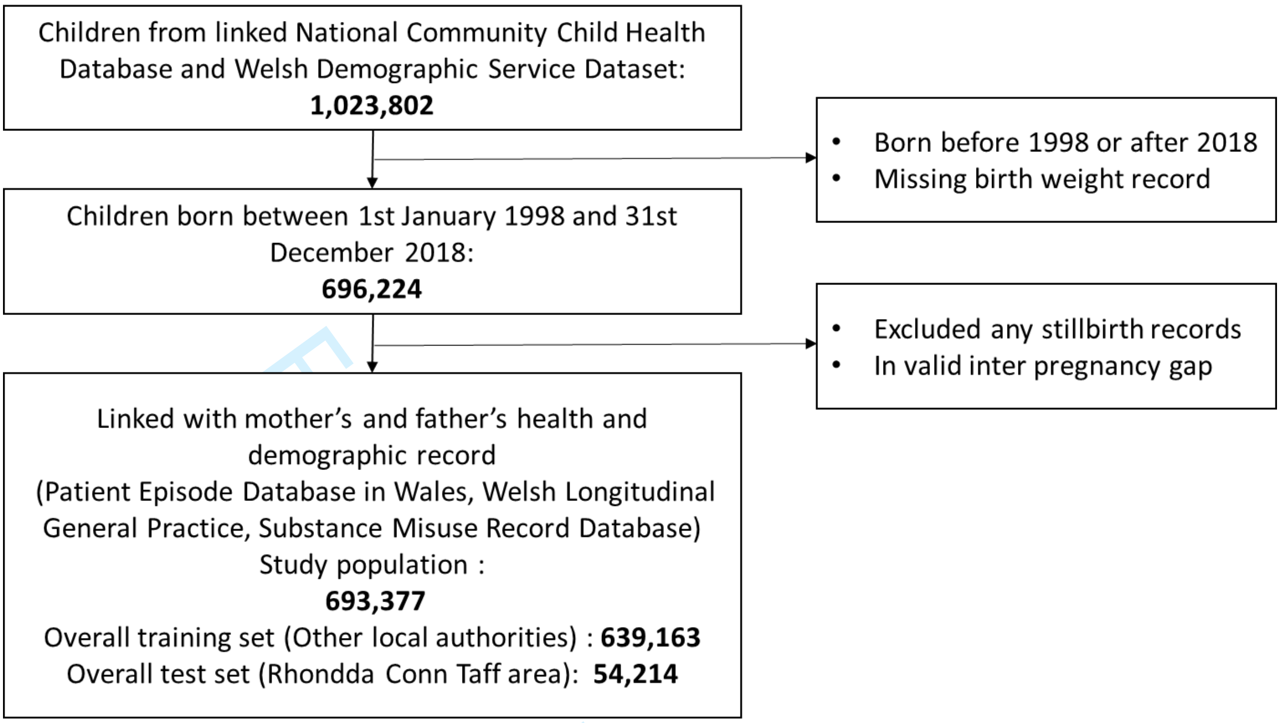

For peer review only

Figure 2: Significant factors associated with the risk LBW among the overall study population.

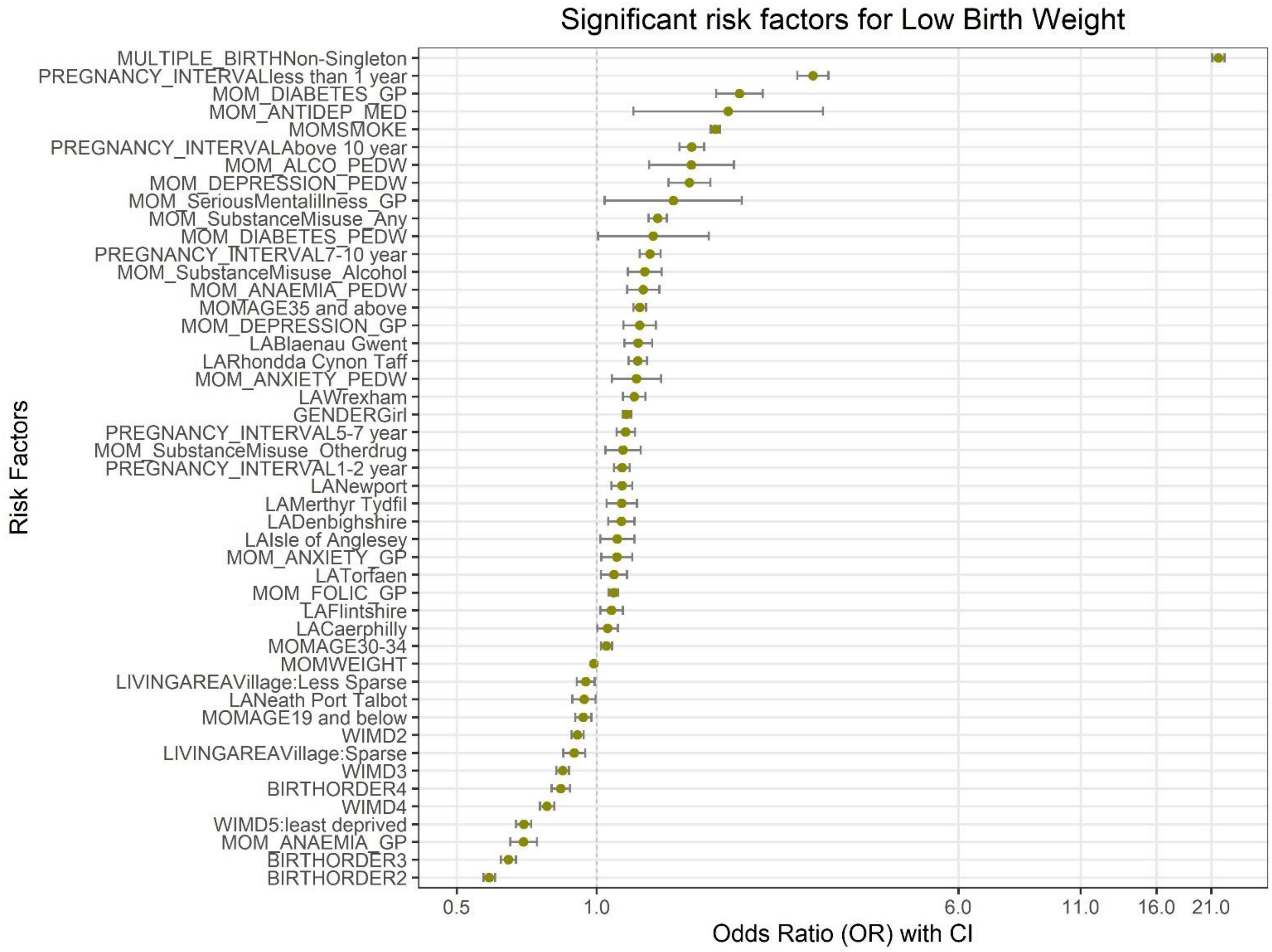

w only

Supplementary Figure 1: Significant risk factors associated with the risk LBW after linking with PPN record

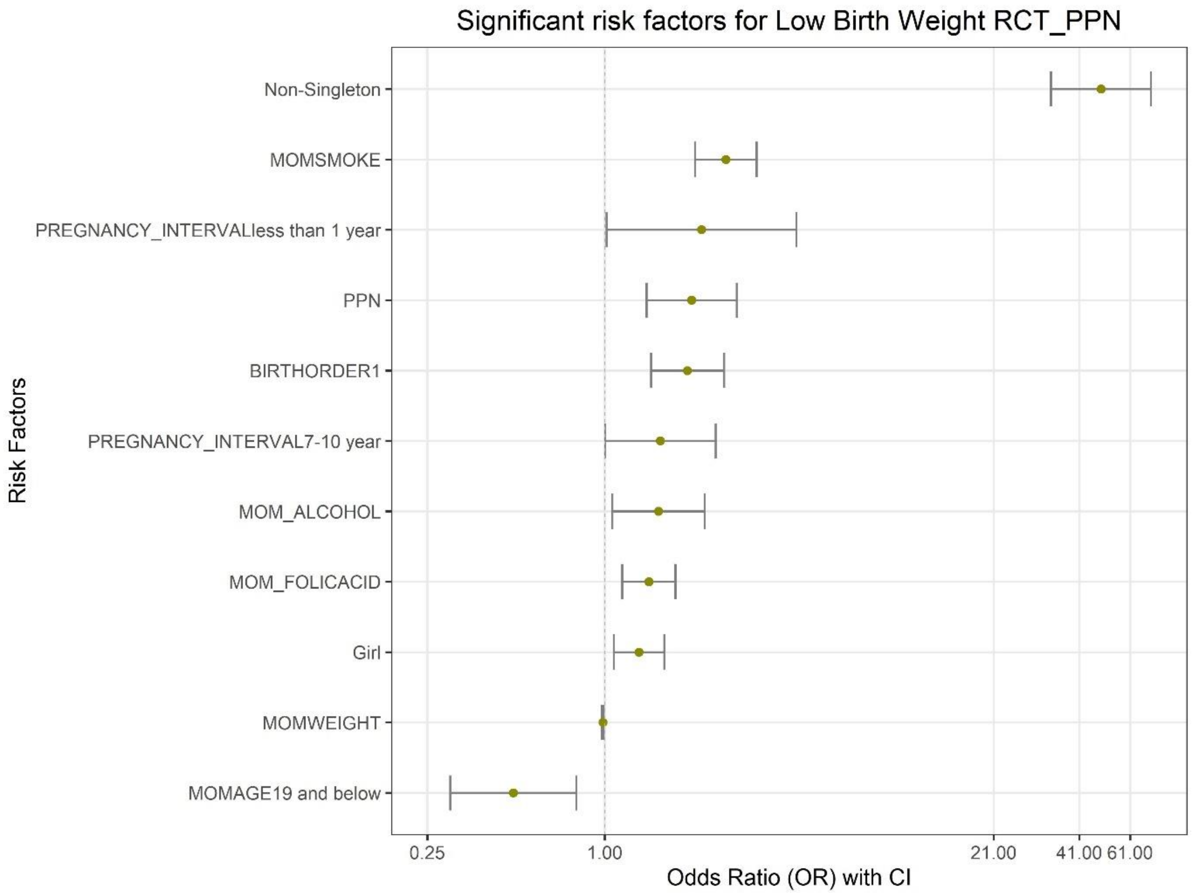

For peer review only

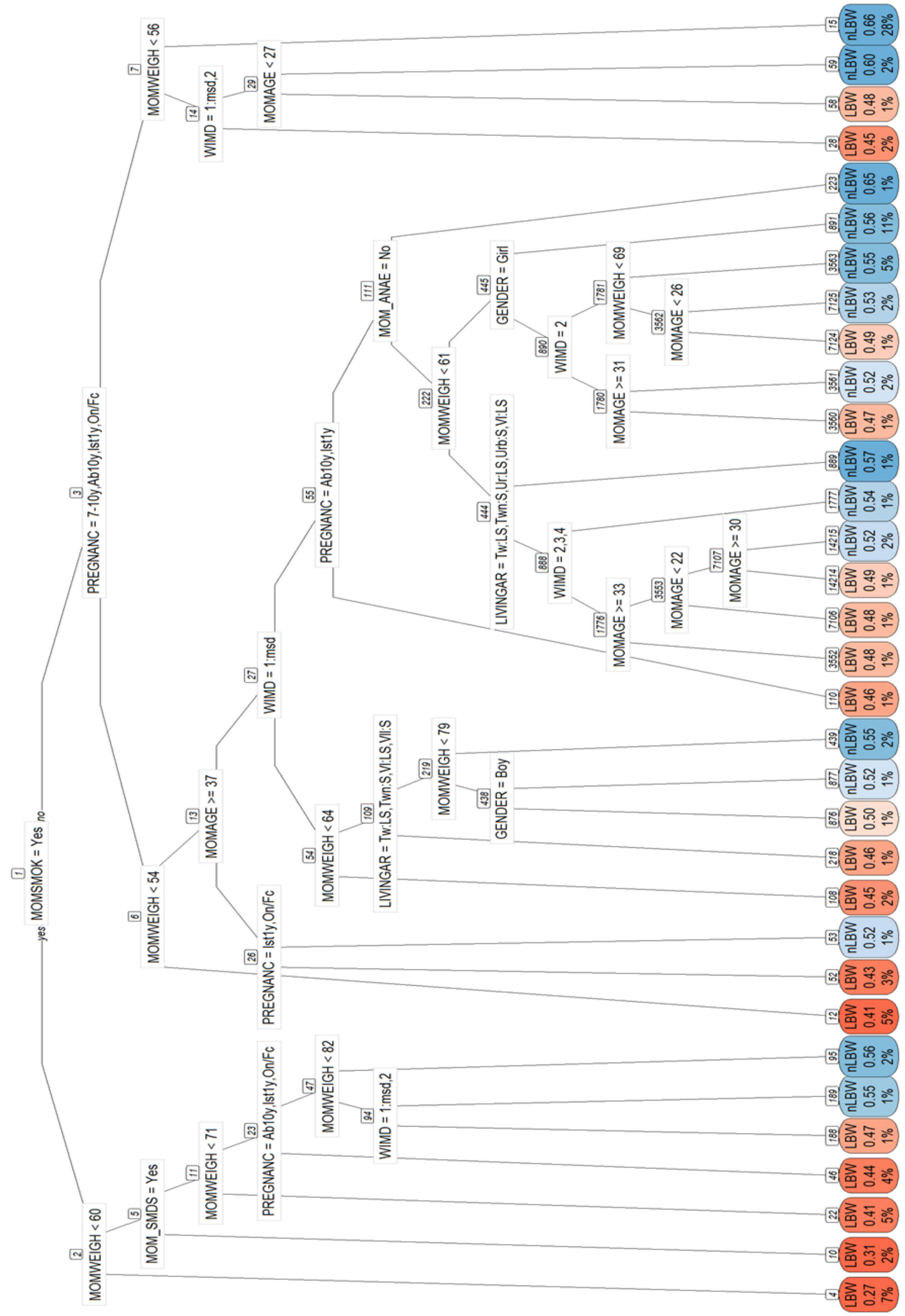

Supplementary Figure 2: Decision tree for singleton children

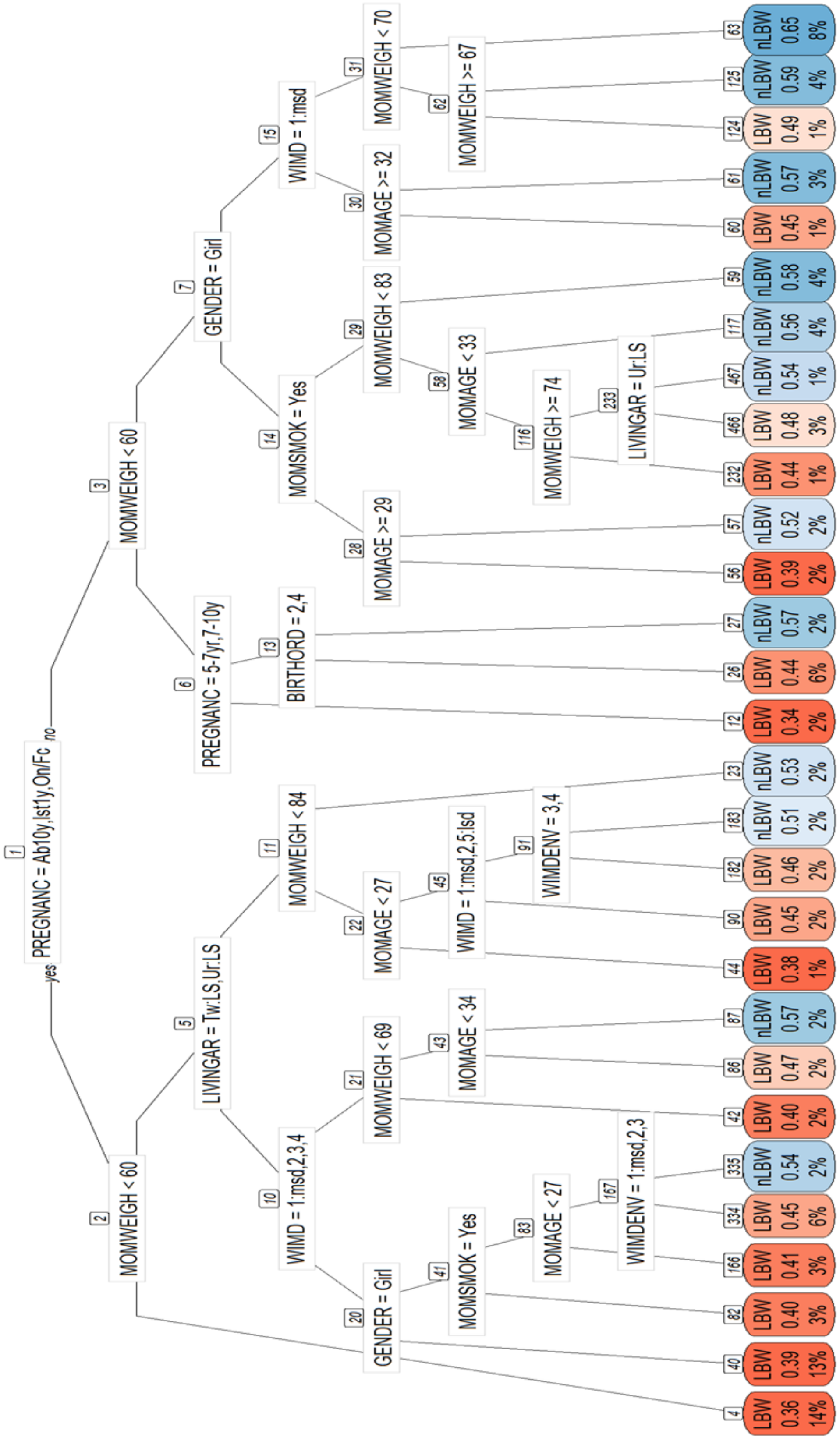

Supplementary Figure 3: Decision tree for non-singleton children

Supplementary Table1: Variables and their source datasets

Variables	NCCHD	MID	WLGP	PEDW	WDS	SMD	Derived	Description if derived
Gender	+							
Maternal age	+							
Gestational age	+							
Birth weight	+							
Birth order	+							
Pregnancy interval							+	Pregnancy interval, in week format, was derived using the birth order, week of birth (the Monday of the week of date of birth), of the previous child and the current child, maternal identifier, and the multiple birth flag.
Multiple birth flag							+	Using the week of birth, encrypted maternal identifier and the birth order, a binary variable – 'multiple birth flag' was derived to distinguish between singleton and non-singleton birth.
Mother weight (kg)							+	The maternal weight during pregnancy was obtained from MID and WLGP. The final

For peer review only

								maternal weight variable was derived following cleaning and harmonising it with the source variables which includes removing and recoding missing, erroneous, and inconsistent records.
Maternal smoking	+	+	+					A cleaned and harmonised variable of maternal smoking during pregnancy was created based on the data obtained from three sources.
WIMD					+			
Diabetes			+	+				
Depression			+	+				
Serious Mental Illness			+					
Anxiety			+	+				
Anti-depressant medication			+					
Vitamin D			+					
FOLIC Acid			+					
Anaemia			+	+				
Alcohol			+	+				
Assault				+				
Substance misuse						+		
Living area					+			
Local authority					+			

For peer review only

Supplementary Table 2: Characteristics of the study population

Variables	Overall training set (n = 639,163)		Overall test set (n = 54,214)		Overall (n = 693,377)	
Gender						
Girl	311,193	48.69%	26,689	49.23%	337,882	48.73%
Boy	327,920	51.30%	27,522	50.77%	355,442	51.26%
Unknown/NULL	50	0.01%	<5	-	-	-
Maternal age						
Less than 19	46,668	7.30%	5,156	9.51%	51,824	7.47%
20-24	133,792	20.93%	13,149	24.25%	146,941	21.19%
25-29	181,233	28.35%	16,454	30.35%	197,687	28.51%
30-34	170,957	26.75%	12,938	23.86%	183,895	26.52%
35 and above	105,869	16.56%	6,513	12.01%	112,382	16.21%
Unknown/NULL	644	0.10%	<5	-	-	-
Birth order						
1st child	319,093	49.92%	26,552	48.98%	345,645	49.85%
2nd child	212,155	33.19%	18,672	34.44%	230,827	33.29%
3rd child	74,724	11.69%	6,407	11.82%	81,131	11.70%
4th or above	33,191	5.19%	2,583	4.76%	35,774	5.16%
Pregnancy interval						
Only/First child	319,093	49.92%	26,552	48.98%	345,645	49.85%
less than 1 year	5,708	0.89%	526	0.97%	6,234	0.90%
1-2 years	67,986	10.64%	5,333	9.84%	73,319	10.57%
2-5 years	162,590	25.44%	13,519	24.94%	176,109	25.40%
5-7 years	41,060	6.42%	4,081	7.53%	45,141	6.51%
7-10 years	27,161	4.25%	2,707	4.99%	29,868	4.31%
Above 10 years	15,565	2.44%	1,496	2.76%	17,061	2.46%
Gestational age (week)						
1: Extremely pre-term: <28 week	2,361	0.37%	208	0.38%	2,569	0.37%
2: Very pre-term: 28-31	5,296	0.83%	565	1.04%	5,861	0.85%
3: Pre-term: 32-36	38,565	6.03%	3,664	6.76%	42,229	6.09%
4: term: 37-42	577,104	90.29%	49,540	91.38%	626,644	90.38%
5: Late term: 43-45	3,909	0.61%	91	0.17%	4,000	0.58%
Unknown/NULL	11,928	1.87%	146	0.27%	12,074	1.74%
Birth weight (gm)						
1: BW≤ 1,000	3,010	0.47%	246	0.45%	3,256	0.47%
2: BW 1,001 - 1,500	4,372	0.68%	445	0.82%	4,817	0.69%
3: BW 1,501 - 2,499	39,143	6.12%	3,923	7.24%	43,066	6.21%
4: BW 2,500- 4,000	521,172	81.54%	44,524	82.13%	565,696	81.59%
5: BW 4,001 - 4,500	61,658	9.65%	4,413	8.14%	66,071	9.53%
6: BW 4,501 - 5000	9,808	1.53%	663	1.22%	10,471	1.51%
Low Birth Weight (LBW)						
nLBW	594,408	93.00%	49,734	91.74%	644,142	92.90%
LBW	44,755	7.00%	4,480	8.26%	49,235	7.10%

For peer review only

Multiple birth flag						
Singleton	619,458	96.92%	52,583	96.99%	672,041	96.92%
Non-singleton	19,705	3.08%	1,631	3.01%	21,336	3.08%
Maternal smoking						
No	502,914	78.68%	41,344	76.26%	544,258	78.49%
Yes	136,249	21.32%	12,870	23.74%	149,119	21.51%
Welsh Index of Multiple Deprivation						
1 (most deprived)	147,204	23.03%	17,946	33.10%	165,150	23.82%
2	118,271	18.50%	17,711	32.67%	135,982	19.61%
3	117,242	18.34%	7,089	13.08%	124,331	17.93%
4	104,056	16.28%	3,646	6.73%	107,702	15.53%
5 (least deprived)	94,190	14.74%	6,242	11.51%	100,432	14.48%
Unknown/NULL	58,200	9.11%	1,580	2.91%	59,780	8.62%
Diabetes GP (mother)						
No	638,628	99.92%	54,149	99.88%	692,777	99.91%
Yes	535	0.08%	65	0.12%	600	0.09%
Diabetes PEDW (mother)						
No	636,104	99.52%	53,978	99.56%	690,082	99.52%
Yes	3,059	0.48%	236	0.44%	3,295	0.48%
Depression GP (mother)						
No	631,230	98.76%	53,323	98.36%	684,553	98.73%
Yes	7,933	1.24%	891	1.64%	8,824	1.27%
Depression PEDW (mother)						
No	634,990	99.35%	53,950	99.51%	688,940	99.36%
Yes	4,173	0.65%	264	0.49%	4,437	0.64%
Serious Mental Illness (mother)						
No	638,887	99.96%	54,185	99.95%	693,072	99.96%
Yes	276	0.04%	29	0.05%	305	0.04%
Anxiety GP (mother)						
No	629,681	98.52%	53,131	98.00%	682,812	98.48%
Yes	9,482	1.48%	1,083	2.00%	10,565	1.52%
Anxiety PEDW (mother)						
No	635,910	99.49%	53,967	99.54%	689,877	99.50%
Yes	3,253	0.51%	247	0.46%	3,500	0.50%
Anti-depressant medication (mother)						
No	639,019	99.98%	54,194	99.96%	693,213	99.98%
Yes	144	0.02%	20	0.04%	164	0.02%
Vitamin D (mother)						
No	637,171	99.69%	54,170	99.92%	691,341	99.71%
Yes	1,992	0.31%	44	0.08%	2,036	0.29%
FOLIC Acid (mother)						
No	486,360	76.09%	37,663	69.47%	524,023	75.58%
Yes	152,803	23.91%	16,551	30.53%	169,354	24.42%

For peer review only

Anaemia GP (mother)						
No	621,276	97.20%	52,636	97.09%	673,912	97.19%
Yes	17,887	2.80%	1,578	2.91%	19,465	2.81%
Anaemia PEDW (mother)						
No	631,370	98.78%	53,883	99.39%	685,253	98.83%
Yes	7,793	1.22%	331	0.61%	8,124	1.17%
Alcohol - GP (mother)						
No	601,660	94.13%	50,836	93.77%	652,496	94.10%
Yes	37,503	5.87%	3,378	6.23%	40,881	5.90%
Alcohol - PEDW (mother)						
No	638532	99.90%	54177	99.93%	692,709	99.90%
Yes	631	0.10%	37	0.07%	668	0.10%
Assault - PEDW (mother)						
No	638,479	99.89%	54165	99.91%	692,644	99.89%
Yes	684	0.11%	49	0.09%	733	0.11%
Substance misuse – any (mother)						
No	607,433	95.04%	51,087	94.23%	658,520	94.97%
Yes	31,730	4.96%	3,127	5.77%	34,857	5.03%
Substance misuse - alcohol (mother)						
No	631,148	98.75%	53,235	98.19%	684,383	98.70%
Yes	7,767	1.22%	975	1.80%	8,742	1.26%
Unknown/NULL	248	0.04%	<5	-	-	-
Substance misuse - other (mother)						
No	632,443	98.95%	53,381	98.46%	685,824	98.91%
Yes	6,199	0.97%	794	1.46%	6,993	1.01%
Unknown/NULL	521	0.08%	39	0.07%	560	0.08%
Mother weight (kg)						
Average (before imputation)	71		72.39		71.06	
Median (before imputation)					67.58	
Average (after imputation)					70.82	
Median (after imputation)					67.00	
Living area						
Town and Fringe - Less Sparse	82,435	12.90%	12,702	23.43%	95,137	13.72%
Town and Fringe - Sparse	22,106	3.46%	71	0.13%	22,177	3.20%
Urban > 10K - Less Sparse	403,591	63.14%	37,924	69.95%	441,515	63.68%
Urban > 10K - Sparse	13,441	2.10%	37	0.07%	13,478	1.94%
Village, Hamlet & Isolated Dwellings - Less Sparse	46,166	7.22%	1,464	2.70%	47,630	6.87%
Village, Hamlet & Isolated Dwellings - Sparse	48,314	7.56%	169	0.31%	48,483	6.99%
Unknown/NULL	23,110	3.62%	1,847	3.41%	24,957	3.60%
Local authority						
Blaenau Gwent					15,008	2.16%
Bridgend					28,018	4.04%
Caerphilly					40,418	5.83%

For peer review only

Cardiff					80,247	11.57%
Carmarthenshire					34,705	5.01%
Ceredigion					11,090	1.60%
Conwy					20,389	2.94%
Denbighshire					19,697	2.84%
Flintshire					32,471	4.68%
Gwynedd					23,249	3.35%
Isle of Anglesey					13,941	2.01%
Merthyr Tydfil					13,259	1.91%
Monmouthshire					14,899	2.15%
Neath Port Talbot					28,854	4.16%
Newport					35,153	5.07%
Pembrokeshire					23,929	3.45%
Powys					20,546	2.96%
Rhondda Cynon Taff					54,214	7.82%
Swansea					49,588	7.15%
Torfaen					20,500	2.96%
Vale of Glamorgan					25,657	3.70%
Wrexham					29,346	4.23%
Unknown/NULL					58,199	8.39%

For peer review only

Supplementary Table 3: Multivariable logistic regression model to identify the risk factors of LBW among the overall study population.

Variable name in model (description)	OR	Lower CI	Upper CI
GENDER (Gender)			
Boy	1		
Girl	1.16	1.14	1.18
MOMSMOKE (Maternal smoking)			
No	1		
Yes	1.80	1.76	1.84
MOMAGE (Maternal age)			
Less than 19	0.94	0.90	0.97
20-24	1.00	0.97	1.03
25-29	1		
30-34	1.05	1.02	1.09
35 and above	1.24	1.20	1.29
BIRTHORDER (Birth order)			
1st child	1		
2nd child	0.59	0.57	0.60
3rd child	0.65	0.62	0.67
4th or above	0.84	0.80	0.88
PREGNANCY_INTERVAL (Pregnancy interval)			
Less than 1 year	2.92	2.70	3.15
1-2 years	1.13	1.09	1.18
2-5 years	1		
5-7 years	1.15	1.10	1.21
7-10 years	1.30	1.24	1.37
Above 10 years	1.60	1.51	1.70
MULTIPLE_BIRTH (Multiple birth flag)			
Singleton			
Non-singleton	21.74	21.09	22.40
WIMD (Welsh Index of Multiple Deprivation)			
1 (most deprived)	1		
2	0.91	0.88	0.94
3	0.84	0.82	0.87
4	0.78	0.75	0.81
5 (least deprived)	0.70	0.67	0.72
WIMDENV (Welsh Index of Multiple Deprivation – Environment score)			
1 (most deprived)	1		
2	0.99	0.96	1.02
3	1.01	0.98	1.04
4	1.02	0.99	1.06
5 (least deprived)	1.00	0.96	1.03
LA (Local authority)			
Blaenau Gwent	1.23	1.15	1.32

For peer review only

Bridgend	1.01	0.95	1.07
Caerphilly	1.06	1.00	1.11
Cardiff	1		
Carmarthenshire	1.02	0.96	1.08
Ceredigion	0.91	0.83	1.01
Conwy	1.05	0.98	1.12
Denbighshire	1.13	1.06	1.21
Flintshire	1.08	1.02	1.14
Gwynedd	1.04	0.97	1.12
Isle of Anglesey	1.11	1.02	1.20
Merthyr Tydfil	1.13	1.05	1.22
Monmouthshire	1.02	0.94	1.10
Neath Port Talbot	0.94	0.89	1.00
Newport	1.13	1.08	1.19
Pembrokeshire	1.04	0.97	1.11
Powys	1.01	0.93	1.09
Rhondda Cynon Taff	1.23	1.17	1.28
Swansea	0.97	0.93	1.02
Torfaen	1.09	1.02	1.16
Vale of Glamorgan	0.98	0.92	1.04
Wrexham	1.20	1.14	1.27
MOM_DIAB_GP (Diabetes GP (mother))			
No	1		
Yes	2.03	1.81	2.28
MOM_DIAB_PEDW (Diabetes PEDW (mother))			
No	1		
Yes	1.32	1.01	1.74
MOM_DEPRE_GP (Depression GP (mother))			
No	1		
Yes	1.24	1.14	1.34
MOM_DEPRE_PEDW (Depression PEDW (mother))			
No	1		
Yes	1.58	1.43	1.75
MOM_SeriousMentalillness_GP (Serious Mental Illness (mother))			
No	1		
Yes	1.46	1.04	2.05
MOM_VITD_GP (Vitamin D (mother))			
No	1		
Yes	1.15	0.96	1.38
MOM_FOLIC_GP			
No	1		
Yes	1.09	1.06	1.11
MOM_ALCO_GP (Alcohol - GP (mother))			
No	1		

For peer review only

Yes	1.02	0.98	1.06
MOM_ALCO_PEDW (Alcohol -PEDW (mother))			
No	1		
Yes	1.60	1.30	1.97
MOM_ANXIETY_GP (Anxiety GP (mother))			
No	1		
Yes	1.10	1.02	1.19
MOM_ANXIETY_PEDW (Anxiety PEDW (mother))			
No			
Yes	1.22	1.08	1.38
MOM_ANTIDEP_MED (Anti-depressant medication (mother))			
No	1		
Yes	1.92	1.20	3.07
MOM_ANAEMIA_GP (Anaemia GP (mother))			
No	1		
Yes	0.70	0.65	0.74
MOM_ANAEMIA_PEDW (Anaemia PEDW (mother))			
No	1		
Yes	1.26	1.16	1.36
MOM_ASSAULT (Assault - PEDW (mother))			
No	1		
Yes	1.16	0.91	1.47
MOM_SubstanceMisuse_Any (Substance misuse – any (mother))			
No	1		
Yes	1.35	1.29	1.41
MOM_SubstanceMisuse_Alcohol (Substance misuse - alcohol (mother))			
No	1		
Yes	1.27	1.17	1.38
MOM_SubstanceMisuse_Otherdrug (Substance misuse - other (mother))			
No	1		
Yes	1.14	1.04	1.24
MOMWEIGHT (Mother weight)			
	0.99	0.99	0.99
LIVINGAREA (Living area)			
Town and Fringe - Less Sparse	1.02	0.95	1.09
Town and Fringe - Sparse	1.03	0.96	1.12
Urban > 10K - Less Sparse	1		
Urban - Sparse	1.02	0.99	1.05
Village, Hamlet & Isolated Dwellings - Less Sparse	0.95	0.91	0.99
Village, Hamlet & Isolated Dwellings - Sparse	0.89	0.85	0.94

For peer review only

Supplementary Table 4: Distribution of LBW and nLBW children based on their multiple birth flags

	Overall training set (n = 639,163)		Overall test set (n = 54,214)		Total (n = 693,377)	
Singleton						
nLBW	585,163	94.46%	49,145	93.46%	634,308	94.39%
LBW	34,295	5.54%	3,438	6.54%	37,733	5.61%
Non-singleton						
nLBW	9,245	46.92%	589	36.11%	9,834	46.09%
LBW	10,460	53.08%	1,042	63.89%	11,502	53.91%

For peer review only

STROBE Statement—Checklist of items that should be included in reports of ***cohort studies***

	Item No	Recommendation	Page No
**Title and abstract**	1	(*a*) Indicate the study's design with a commonly used term in the title or the abstract	1
		(*b*) Provide in the abstract an informative and balanced summary of what was done and what was found	2
**Introduction**			
Background/rationale	2	Explain the scientific background and rationale for the investigation being reported	4
Objectives	3	State specific objectives, including any prespecified hypotheses	4
**Methods**			
Study design	4	Present key elements of study design early in the paper	5
Setting	5	Describe the setting, locations, and relevant dates, including periods of recruitment, exposure, follow-up, and data collection	5
Participants	6	(*a*) Give the eligibility criteria, and the sources and methods of selection of participants. Describe methods of follow-up	5
		(*b*) For matched studies, give matching criteria and number of exposed and unexposed	
Variables	7	Clearly define all outcomes, exposures, predictors, potential confounders, and effect modifiers. Give diagnostic criteria, if applicable	5-6
Data sources/ measurement	8*	For each variable of interest, give sources of data and details of methods of assessment (measurement). Describe comparability of assessment methods if there is more than one group	5-6
Bias	9	Describe any efforts to address potential sources of bias	5-6
Study size	10	Explain how the study size was arrived at	5
Quantitative variables	11	Explain how quantitative variables were handled in the analyses. If applicable, describe which groupings were chosen and why	
Statistical methods	12	(*a*) Describe all statistical methods, including those used to control for confounding	6
		(*b*) Describe any methods used to examine subgroups and interactions	
		(*c*) Explain how missing data were addressed	
		(*d*) If applicable, explain how loss to follow-up was addressed	
		(*e*) Describe any sensitivity analyses	
**Results**			
Participants	13*	(a) Report numbers of individuals at each stage of study—eg numbers potentially eligible, examined for eligibility, confirmed eligible, included in the study, completing follow-up, and analysed	7
		(b) Give reasons for non-participation at each stage	
		(c) Consider use of a flow diagram	
Descriptive data	14*	(a) Give characteristics of study participants (eg demographic, clinical, social) and information on exposures and potential confounders	7
		(b) Indicate number of participants with missing data for each variable of interest	
		(c) Summarise follow-up time (eg, average and total amount)	
Outcome data	15*	Report numbers of outcome events or summary measures over time	7

For peer review only

Main results	16	(*a*) Give unadjusted estimates and, if applicable, confounder-adjusted estimates and their precision (eg, 95% confidence interval). Make clear which confounders were adjusted for and why they were included<br>(*b*) Report category boundaries when continuous variables were categorized<br>(*c*) If relevant, consider translating estimates of relative risk into absolute risk for a meaningful time period	11,14-16
Other analyses	17	Report other analyses done—eg analyses of subgroups and interactions, and sensitivity analyses	14
**Discussion**			
Key results	18	Summarise key results with reference to study objectives	16
Limitations	19	Discuss limitations of the study, taking into account sources of potential bias or imprecision. Discuss both direction and magnitude of any potential bias	17
17Interpretation	20	Give a cautious overall interpretation of results considering objectives, limitations, multiplicity of analyses, results from similar studies, and other relevant evidence	16-17
Generalisability	21	Discuss the generalisability (external validity) of the study results	17-18
**Other information**			
Funding	22	Give the source of funding and the role of the funders for the present study and, if applicable, for the original study on which the present article is based	18

*Give information separately for exposed and unexposed groups.

Note: An Explanation and Elaboration article discusses each checklist item and gives methodological background and published examples of transparent reporting. The STROBE checklist is best used in conjunction with this article (freely available on the Web sites of PLoS Medicine at http://www.plosmedicine.org/, Annals of Internal Medicine at http://www.annals.org/, and Epidemiology at http://www.epidem.com/). Information on the STROBE Initiative is available at http://www.strobe-statement.org.

For peer review only

BMJ Open

Weighting of risk factors for low birth weight: A linked routine data cohort study in Wales, UK.

Journal:	*BMJ Open*
Manuscript ID	bmjopen-2022-063836.R1
Article Type:	Original research
Date Submitted by the Author:	10-Nov-2022
Complete List of Authors:	Bandyopadhyay, Amrita; Swansea University, National Centre for Population Health and Wellbeing Research<br>Jones, Hope; Swansea University, National Centre for Population Health and Wellbeing Research<br>Parker, Michael; Swansea University, National Centre for Population Health and Wellbeing Research<br>Marchant, Emily ; Swansea University, National Centre for Population Health and Wellbeing Research<br>Evans, Julie; Public Health Wales, Keir Hardie University Health Park<br>Todd, Charlotte; Public Health Wales, Keir Hardie University Health Park<br>Rahman, Muhammad A.; Cardiff Metropolitan University, Cardiff School of Technologies<br>Healy, James; Swansea University, National Centre for Population Health and Wellbeing Research; Office for National Statistics<br>Win, Tint ; Swansea University, National Centre for Population Health and Wellbeing Research<br>Rowe, Ben; National Police Chiefs' Council<br>Moore, Simon; Cardiff University, School of Dentistry; Cardiff University, Security, Crime, Intelligence Institute<br>Jones, Angela; Public Health Wales, Keir Hardie University Health Park<br>Brophy, Sinead; Swansea University, National Centre for Population Health and Wellbeing
**Primary Subject Heading**:	Epidemiology
Secondary Subject Heading:	Public health
Keywords:	EPIDEMIOLOGY, PUBLIC HEALTH, STATISTICS & RESEARCH METHODS

SCHOLARONE™
Manuscripts

I, the Submitting Author has the right to grant and does grant on behalf of all authors of the Work (as defined in the below author licence), an exclusive licence and/or a non-exclusive licence for contributions from authors who are: i) UK Crown employees; ii) where BMJ has agreed a CC-BY licence shall apply, and/or iii) in accordance with the terms applicable for US Federal Government officers or employees acting as part of their official duties; on a worldwide, perpetual, irrevocable, royalty-free basis to BMJ Publishing Group Ltd ("BMJ") its licensees and where the relevant Journal is co-owned by BMJ to the co-owners of the Journal, to publish the Work in this journal and any other BMJ products and to exploit all rights, as set out in our licence.

The Submitting Author accepts and understands that any supply made under these terms is made by BMJ to the Submitting Author unless you are acting as an employee on behalf of your employer or a postgraduate student of an affiliated institution which is paying any applicable article publishing charge ("APC") for Open Access articles. Where the Submitting Author wishes to make the Work available on an Open Access basis (and intends to pay the relevant APC), the terms of reuse of such Open Access shall be governed by a Creative Commons licence – details of these licences and which Creative Commons licence will apply to this Work are set out in our licence referred to above.

Other than as permitted in any relevant BMJ Author's Self Archiving Policies, I confirm this Work has not been accepted for publication elsewhere, is not being considered for publication elsewhere and does not duplicate material already published. I confirm all authors consent to publication of this Work and authorise the granting of this licence.

For peer review only

Weighting of risk factors for low birth weight: A linked routine data cohort study in Wales, UK.

Amrita Bandyopadhyay[1*], Hope Jones[1], Michael Parker[1], Emily Marchant[1], Julie Evans[2], Charlotte Todd[2], Muhammad A. Rahman[3], James Healy [1,4], Tint Lwin Win[1], Ben Rowe[5], Simon Moore[6,7], Angela Jones[2], Sinead Brophy[1].

1. National Centre for Population Health and Wellbeing Research, Swansea University Medical School, Wales, SA2 8PP, UK
2. Public Health Wales, Keir Hardie University Health Park, CF48 1BZ, Wales. UK
3. Cardiff School of Technologies, Cardiff Metropolitan University, Llandaff Campus, CF5 2YB, Wales, UK
4. Office for National Statistics, Government Buildings, Cardiff Road, Duffryn, Newport, NP10 8XG, UK
5. National Police Chiefs' Council Lead for Mental Health and Age, 10 Victoria Street, London, SW1H 0NN, UK
6. Violence Research Group, School of Dentistry, Cardiff University, Cardiff, Wales, CF14 4XY, UK
7. Security, Crime & Intelligence Institute, Cardiff University, SPARK, Maindy Road, Cardiff, Wales, CF10 3AE, UK

* Corresponding author

Name of the Corresponding Author: Amrita Bandyopadhyay

Affiliations: National Centre for Population Health and Wellbeing Research, Swansea University Medical School, Wales, SA2 8PP, UK

Email Address: a.bandyopadhyay@swansea.ac.uk

List of keywords: Low birth weight, Maternal health, Pregnancy interval, Data linkage, Cohort study

For peer review only

Abstract

Objective

Globally, 20 million children are born with a birth weight below 2,500 grams every year, which is considered as a low birthweight (LBW) baby. This study investigates the contribution of modifiable risk factors in a nationally representative Welsh e-cohort of children and their mothers to inform opportunities to reduce LBW prevalence.

Design

A longitudinal cohort study based on anonymously linked, routinely collected multiple administrative datasets.

Participants

The cohort, (N=693,377) comprising of children born between 1st January 1998 and 31st December 2018 in Wales, was selected from the National Community Child Health database.

Outcome measures

The risk factors associated with a binary LBW (outcome) variable were investigated with multivariable logistic regression (MLR) and decision tree (DT) models.

Results

The MLR model showed that non-singleton children had the highest risk of LBW (adjusted odds ratio 21.74 (95% confidence interval 21.09,22.40)), followed by pregnancy interval less than one year (2.92(2.70,3.15)), maternal physical and mental health conditions including diabetes (2.03(1.81,2.28)), anaemia (1.26(1.16,1.36)), depression (1.58(1.43,1.75)), serious mental illness (1.46(1.04,2.05)), anxiety (1.22(1.08,1.38)) and use of anti-depressant medication during pregnancy (1.92(1.20,3.07)). Additional maternal risk factors include smoking (1.80(1.76,1.84)), alcohol-related hospital admission (1.60(1.30,1.97)), substance misuse (1.35(1.29,1.41)) and evidence of domestic abuse (1.98(1.39,2.81)). Living in less deprived area has lower risk of LBW (0.70(0.67,0.72)). The most important risk factors from the DT models include maternal factors such as smoking, maternal weight, substance misuse record, maternal age along with deprivation - WIMD score, pregnancy interval and birth order of the child.

For peer review only - http://bmjopen.bmj.com/site/about/guidelines.xhtml

Conclusion

Resources to reduce the prevalence of LBW should focus on improving maternal health, reducing pre-term births, increasing awareness of what is a sufficient pregnancy interval, and to provide adequate support for mothers' mental health and wellbeing.

For peer review only

For peer review only - http://bmjopen.bmj.com/site/about/guidelines.xhtml

Strengths and limitations of this study

- This study has built an e-cohort using data-linkage across multiple routinely collected administrative datasets to investigate the risk factors of low birthweight for the population of Wales.
- The study has investigated the modifiable risk factors of LBW in a holistic framework by linking primary and secondary care physical and mental health, socio- demographic and pregnancy related routine data including police record for a nationally representative sample.
- This study undertook two different statistical approaches (regression analysis and data-driven machine learning algorithm) which is a strength of the study.
- This work were unable to include any important risk factors which were not recorded in the health care system or any conditions which were undiagnosed hence that did not result in the system.

For peer review only

For peer review only - http://bmjopen.bmj.com/site/about/guidelines.xhtml

Introduction

The World Health Organisation (WHO) defines low birth weight (LBW) as infants weighting less than 2,500 grams (5.5 pounds) irrespective of gestational age [1,2]. Latest figures show that each year around 53,000 live births (6.9%) are identified as LBW in the UK [3]. LBW is the result of intra-uterine growth restriction (less than 10th centile of weight for sex and gestational age), prematurity (gestational age less than 37 weeks), or a combination of both [4]. LBW can impair the baby's cognitive development and lead to developmental disabilities and poor academic achievement [5]. Furthermore, LBW significantly increases the risk of perinatal and neonatal mortality and longstanding morbidity in early and later life [6]. Whilst there has been a reduction in mortality amongst preterm infants in the last two decades, the incidence of preterm birth has increased in many developed countries [6–8]. The increase is also associated with preterm delivery of multiple pregnancies, with medically indicated preterm birth 10 times more likely in multiple pregnancies than singleton births [9]. To address the global burden of LBW, the Sixty Fifth World Health Assembly Resolution 65.6 endorsed a comprehensive implementation plan to achieve a 30% reduction in LBW by 2025 [1]. A study conducted on the birth data from 148 countries of 195 United Nations' member states indicated that there had been a 2.9% reduction in the LBW prevalence in 2015, compared to 2000 worldwide. However, there has not been any change in the LBW prevalence in high income regions (including Europe) and the progress is slower than required to meet the WHO LBW target by 2025 [10].

Existing research has found factors linked with mothers, such as age, high deprivation, and low academic qualification, are associated with increased odds of LBW [11,12]. Modifiable risk factors for LBW include inter-pregnancy interval [13], maternal physical [14–17] and mental health [18,19], and environmental exposures during pregnancy [20]. Studies have also shown numerous health behaviours such as smoking [21,22], alcohol intake (in which there is a dose-response relationship with LBW) [23], and/or illicit drug use [24] during pregnancy are modifiable risk factors of LBW. Indirect (negative maternal behaviours, inadequate nutrition or prenatal care, and increased stress) or direct (physical assault, sexual trauma) experience of intimate partner abuse during pregnancy can lead to adverse infant outcomes including LBW [25,26].

It is important to gain an understanding of these risk factors, particularly modifiable risk factors, so that resources and interventions can be scheduled effectively. Moreover, the wide range of risk factors cannot be addressed in isolation. Most of the risk factors that are strongly independently associated with LBW are correlated. This study aimed to understand the contributions of risk factors to the burden of LBW for the population of Wales, using traditional statistical methods and supervised machine learning models.

For peer review only - http://bmjopen.bmj.com/site/about/guidelines.xhtml

Method

Participants and linkage

The linked data cohort (N = 693,377) was comprised of children born in Wales between 1st January 1998 and 31st December 2018. The study population was identified in the National Community Child Health database (NCCHD), which is a local Child Health System database held by the National Health Service (NHS). The participants were linked to the Wales-wide administrative register, the Wales Demographic Service Dataset (WDS). Linkage was undertaken using an anonymised encrypted linkage key, the Anonymised Linking Field (ALF), in the Secure Anonymised Information Linkage (SAIL) Databank [27]. WDS provided the anonymised residential linking fields (RALFs), which is an encrypted residential address and its corresponding lower super output area (LSOA, small geographic areas with a population of approximately 1,500) when the child was born. LSOA was linked with the Welsh Index of Multiple Deprivation (WIMD) 2014, which is a measure of relative deprivation. The participants flow diagram is displayed in Figure 1.

Explanatory variables

A literature review was conducted at the beginning of the study to identify the explanatory variables associated with LBW. A study by Johnson et al was identified [3] and this provided the framework upon which the current study was developed. The literature review selected

a) any published systematic reviews since 2013 which focused on risk factors identified in Johnson et al.
b) any published systematic reviews since 2010 for all additional risk factors not identified in Johnson et al.

This study therefore considered a wide range of explanatory and confounding variables that have a plausible causal link to LBW and are potentially modifiable at a population level. The literature review to select the explanatory variables has been described in a Supplementary document. In the current study, modifiable risk factors identified from the literature have been derived from routinely collected electronic datasets to build a Welsh e-cohort of the children. The maternal variables related to a childbirth (maternal age, gestational age, child's birth weight and gender, and birth order of the child) were obtained from NCCHD and Maternal Indicator Database (MID). The variables for maternal physical (such as diabetes, anaemia, intake of Vitamin D and folic acid supplement through prescription) and mental (depression, anti-depressant medication, anxiety, serious mental illness such as bipolar disorder, schizophrenia) health during pregnancy were obtained from primary care Welsh Longitudinal General Practice (WLGP) and hospital admissions dataset known as the Patient Episode

For peer review only - http://bmjopen.bmj.com/site/about/guidelines.xhtml

database in Wales (PEDW). The record of physical assault linked with mothers during pregnancy was obtained from PEDW. The substance misuse database (SMD) provided the information on individuals receiving treatment for alcohol and other substance misuse in Wales. Mothers' who were presenting in this database during pregnancy were considered in the study. Area type (urban/rural) and local authority (LA) under which they lived during the pregnancy and their overall and physical environment quantified in the WIMD were included in this study. A cleaned and harmonised variable of maternal smoking during pregnancy was created based on the data obtained from NCCHD, MIDS and WLGP datasets. The other derived maternal variables include multiple birth flag (to distinguish between singleton and non-singleton), pregnancy interval, and maternal weight. The description of the explanatory variables and their sources have been described in Supplementary Table 1.

A subset of the study population (participants from Rhondda, Cynon, Taf born between June 2016 and 2018) was linked with the Public Protection Notification (PPN) dataset to investigate the impact of the PPN during pregnancy along with other existing risk factors on the risk of LBW [28]. PPN is an information sharing system, completed by police officers that compiles incidents of domestic abuse, stalking or harassment. The current study received PPN data from South Wales Police for residents of South Wales LA Rhondda, Cynon, Taf.

Outcome variable

A binary variable was created using the birth weight variable obtained from NCCHD.

- LBW = birth weight <2,500
- Not LBW (nLBW) = birth weight ≥2,500

Statistical Analysis

It is known that gestational age is highly correlated with LBW. However, as the gestational age is only obtained at the point of birth, making it a non-modifiable risk factor, this study has not considered it as a predictor variable. The models were stratified by the multiple birth as this is one of the main predictors of LBW. The missing records in birth weight variable were removed from the analysis. Since there was around 15% missing data in maternal weight variable, the variable was imputed by the simple random imputation method [29]. The missing data in the other explanatory variables (less than 10%) were recoded as 'Unknown'. The birth record for stillbirth and pregnancy interval of less than 22 weeks (as that is the minimum duration for a considerable gestation period) were also not considered for the statistical analysis. Data preparation including data linkage and data cleaning for this analysis was done on SAIL DB2 SQL platform. All statistical analyses were performed in R version 4.0.3.

For peer review only - http://bmjopen.bmj.com/site/about/guidelines.xhtml

The statistical analysis of the current study was carried out using two statistical approaches a) building a holistic regression model to investigate the association between the risk factors and LBW and b) build a predictive model using supervised classification method. Both the methods were capable of handling binary outcome variable. The models that were developed by the above-mentioned methods were built independently, however they both were informed by the same dataset. This enabled us to evaluate and validate the findings of the models and helped to gain insight on the generalisability of the findings.

Logistic regression

A multivariable logistic regression (MLR) model was developed to identify the most important risk factors associated with LBW. The MLR model was built on the overall study population (whole Wales dataset) to examine the associations between all the explanatory and outcome variables. The holistic model considering all the risk factors identified from literature review and selected or derived from routine data includes maternal physical and mental health during pregnancy, maternal smoking, alcohol and other substance misuse record, maternal age, maternal weight, pregnancy interval, living area, LA and deprivation - WIMD score. MLR model also included birth order of the child and the multiple birth flag. The birth order highlights the sequential birth position of the child for a mother, and it does not vary among the children who were non-singleton in the same family (please see Supplementary Table 1), hence, they were considered as independent variables in the model and their association with the outcome variable was investigated in the MLR model. The importance and significance of the risk factors have been evaluated and presented with their adjusted Odds Ratio (aOR) and 95% confidence interval (CI).

Decision tree

A supervised machined learning classifier - decision tree (DT) model was developed to build a risk profile for LBW and test its predictive performance. Classification tree – DT models were constructed using RPART (Recursive Partitioning And Regression Trees) packages in R [30,31]. The algorithm recursively partitions the data into multiple sub-spaces to obtain the homogeneous final sub-space of predictor variables. For DT, the whole Wales data except for Rhondda, Cynon, Taf was used to train the model and prediction performance was evaluated on a test dataset which consisted of a sample of participants from the LA of Rhondda, Cynon, Taf. This LA was chosen because it had one of the highest rates of LBW in Wales and is an area which would benefit most from an accurate prediction model.

A separate data linkage was undertaken with a subset of the study population which was linked to the mother's domestic abuse record from PPN dataset (the latter was only available for Rhonda Cynon

For peer review only - http://bmjopen.bmj.com/site/about/guidelines.xhtml

Taff). Another adjusted MLR model was developed on this linked data to investigate the risk association for LBW.

Patient and Public Involvement

No patient involved.

Results

The study population consisted of 693,377 children of those 54,214 were from Rhondda, Cynon, Taf and 639,163 were from other LAs. The children from Rhondda, Cynon, Taf, which was later used as a test set for DT were well representative of the Welsh population (see Supplementary Table 2). In the overall study population, 51.26% were boys, 96.92% were singleton and 90.38% children were born full term (gestational age between 37 and 42 weeks). 49.85% children were born as the first child in the family. Mothers of 0.48% children were admitted to hospital for diabetes and 0.09% had a GP visit for diabetes, 1.27% had depression, 1.52% with anxiety and 0.02% were on antidepressant medication during pregnancy. There were 1.26% and 21.51% children whose mothers had alcohol-related substance misuse and smoking record during pregnancy respectively. The average maternal age at birth of child and maternal weight was 28 years and 70.82 kg (after imputation) respectively and 63.68% of them were living in densely populated urban areas. Overall, 7.1% (8.26% in test set and 7% in other LAs) of children were born as LBW.

Factors associated with LBW: MLR results

Non-singleton children were at almost 22 times higher risk of LBW than singleton children (adjusted odds ratio (aOR) – 21.74 (95% confidence interval (CI) 21.09, 22.40)). Mothers with diabetes-related GP visits (2.03 (1.81, 2.28)) and hospital admission records of anaemia (1.26 (1.16, 1.36)) during pregnancy were at very high risk of having LBW children. Poor mental health during pregnancy such as severe depression (1.58 (1.43, 1.75)), serious mental illness (1.46 (1.04, 2.05)), severe anxiety (1.22 (1.08, 1.38)) and antidepressant medications (1.92 (1.20, 3.07)) were risk factors for LBW. The other highly significant modifiable risk factors linked with pregnant mothers include maternal smoking (1.80 (1.76, 1.84)), alcohol related hospital admissions (1.60 (1.30, 1.97)) and any substance misuse (alcohol/other drugs) (1.35 (1.29, 1.41)) during pregnancy. Higher maternal age was also associated with the risk of LBW. Though maternal age less than 19 was significantly associated with the risk of LBW in the univariable model, after adjusting all the other explanatory variables, this did not remain as a risk factor of LBW. The first child born was at higher risk of LBW than subsequent births, The odds of LBW for the 2nd child was 0.59 (0.57, 0.60) compared to the first child. Mothers living in the least

For peer review only - http://bmjopen.bmj.com/site/about/guidelines.xhtml

deprived and rural areas during pregnancy were at lower risk of having LBW children than others living in more deprived and urban areas. The statistically significant risk factors with their aOR and CI have been visualised and described in Figure 2 and Supplementary Table 3.

Finding from the linked PPN data model

A dataset of 5,854 mothers were obtained from the PPN data linkage. Those who had a PPN call during pregnancy, 18% of them had LBW child and those who did not have PPN call, 8.7% of them had LBW child (see Table 1). Mothers with a PPN call during pregnancy had almost 2 times higher risk of having LBW baby (1.98 (1.39, 2.81)) than mothers without PPN call after adjusting for confounding factors (see Supplementary Figure 1).

Table 1: Distribution of LBW and nLBW children for the subset who were linked with mother's PPN record during pregnancy

PPN record during pregnancy	n = 5,854	
No		
nLBW	5,074	91.3%
LBW	485	8.7%
Yes		
nLBW	241	82%
LBW	53	18%

Predictive DT model

Since LBW were disproportionately more prevalent in non-singleton children (5.61% singleton vs 53.91% of the non-singleton children were LBW) (Supplementary Table 4), two separate predictive models using DTs were developed.

Singleton children

There were 619,458 observations in the training model. The most important risk factors selected by the DT algorithm to develop the final tree were maternal smoking, maternal weight, pregnancy interval, birth order, maternal substance misuse record (any), maternal age, deprivation - WIMD score, maternal substance misuse record (other drug) and maternal substance misuse record (alcohol). Supplementary Figure 2 depicts the final tree with the branches including final 33 terminal nodes. For example, the model would predict a LBW baby if a) maternal smoking is positive (e.g., mum smokes during pregnancy) and b) maternal weight less than 60 kg. The number of women in this category who had a LBW child is 73% (see terminal node 4 in Supplementary Figure 2) and risk profile was found in 7% of the training model population (e.g., 7% of pregnant women were smokers who weighed less than 60 kg during pregnancy).

For peer review only

The test data was built on the 52,583 singleton children, which is 7.82% of the total singleton children in this study. The model performance is explained in a confusion matrix with 60.54% accuracy, 60.41% sensitivity, 60.55% specificity, 9.68% positive predictive values and 95.63% negative predictive value (see Table 2 and 3).

Table 2: Confusion matrix/two by two table of the DT (singleton and non-singleton) models

**Prediction**	**Reference** (Singleton) n = 52,583		**Reference** (Non-singleton) n = 1,631	
	LBW	nLBW	LBW	nLBW
LBW	2,077 (TP)	19,389 (FP)	716 (TP)	347 (FP)
nLBW	1,361 (FN)	29,756 (TN)	326 (FN)	242 (TN)

Table 3: Prediction model performance (n=52,583 Singleton, n = 1,631 Non-singleton from test set)

	**Accuracy**	**Sensitivity**	**Specificity**	**Positive Predictive Value**	**Negative Predictive Value**
**DT Singleton model**	60.54%	60.41%	60.55%	09.68%	95.63%
**DT Non-singleton model**	58.74%	68.71%	41.09%	67.36%	42.61%

Non-singleton children

There were 19,705 children in the non-singleton training subset. The variables selected to generate the tree by the DT algorithm in the importance order were pregnancy interval, birth order, maternal weight, maternal age, gender, deprivation - WIMD score, maternal smoking, living area, deprivation - WIMD (environment) score and maternal substance misuse record (any). Supplementary Figure 3 depicts the final tree with the branches including final 29 terminal nodes. For example, the model would predict a LBW baby if a) this is the first child or pregnancy interval is either above 10 years or less than 1 year and b) maternal weight less than 60 kg (terminal node 4).

The test set was built on the 1,631 non-singleton children, which is 7.64% of the total non-singleton children in this study. The model performance was measured as 58.74% accuracy, 68.71% sensitivity,

For peer review only - http://bmjopen.bmj.com/site/about/guidelines.xhtml

41.09% specificity, 67.36% positive predictive values and 42.61% negative predictive value (see Table 2 and 3).

Discussion

7.1% of the overall study population in Wales was LBW between 1998 and 2018. Global trend of LBW is around 7.0% in both 2000 and 2015 for the developed regions (Europe, North America, Australia), which is consistent with our finding [2]. Findings from the Office for National Statistics (ONS) state a combined English and Welsh rate of LBW of 7.0% in 2016, unchanged from 2011 [32]. Our findings show that LBW is strongly associated with non-singleton pregnancy, and maternal health which includes a short pregnancy interval, non-optimal maternal body weight (e.g., low, or high weight), maternal smoking, diabetes, anaemia, mental illness and living in a deprived urban area and exposed to domestic abuse during pregnancy.

The findings of short and long pregnancy intervals being associated with increased odds of LBW has been reported previously [13]. However, Regan *et al.* highlighted that several studies examining long inter-pregnancy interval are prone to measurement error because miscarriages and abortions within this time period is difficult to capture. Hence the authors suggest that caution should be exercised when interpreting these findings [33]. Regarding the association of short-pregnancy intervals with increased odds of LBW, studies using matched controlled designs have argued that this association may be weaker than previously thought [33,34], especially when adjusting for factors such as gestational diabetes, pre-pregnancy obesity, parity and other familial factors [35]. The current study has included diabetes and maternal weight along with pregnancy interval in the analysis. In terms of putting this evidence in context, when considering advice over pregnancy intervals, it will be important to consider all the available evidence including the impact of pregnancy interval on preterm birth and maternal outcomes [36]. Among the modifiable risk factors for LBW identified in this study, smoking during pregnancy is significantly and consistently important. A number of reviews have been carried out in the field of interventions to reduce smoking in pregnancy and this suggest that psychosocial interventions (counselling, feedback and incentives) appear to be effective at supporting women to stop smoking in pregnancy which, in turn, can reduce the proportion of babies born with LBW [37]. However, they argue that the context of the intervention needs to be given consideration and that whilst evidence exists for potentially effective interventions which could be piloted through delivery of programmes locally, efforts should also be directed at population wide strategies to reduce smoking uptake in young women. This may be especially important given the clear difficulties experienced by pregnant women to give up smoking [37]. With regards to our finding of maternal mental health

For peer review only - http://bmjopen.bmj.com/site/about/guidelines.xhtml

affecting the risk of LBW, both severe depression and anxiety were associated with an increased odds of LBW in our study [38].

The study undertook two statistical methods a) regression and b) supervised classification model with the aim that the regression model would identify the risk factors with highest association/Odds Ratio but not frequently observed factors at the population level for e.g., only .09% mothers had diabetes related GP visit during pregnancy, and they had two times higher risk of having a LBW child (2.03 (1.81, 2.28)). However, the DT models consider the number of people affected by the risk factor rather than just strength of association, hence capable of identifying the factors at a population level (such as smoking, deprivation score) that can result in higher risk of LBW.

There are similarities between the findings of our DT models and existing literature utilising machine learning to predict LBW, for e.g., urban living, higher deprivation and poorer families are at higher risk of LBW [39]. The incidence of LBW in this current work is lower than another research utilising machine learning to predict LBW for e.g., Loreto et al has an incidence of 13.45% in work that builds over 60 different machine learning models [40], Ahmadi et al assess logistic regression and random forests in a cohort with LBW rate of 9.5% [41]. The smaller number of active cases in the dataset the more difficult it is to build a prediction model for, particularly without a set of highly associated input variables. In this study, the singleton DT model correctly predicted 60.41% of all the true positive cases. However, the low positive predictive value of 9.68% indicates that the model assigned a false positive 'LBW' classification for 89.32% cases. This model only includes singleton children and since non-singleton pregnancies are highly associated with LBW, removing this variable from the model has lessened its predictive capability. This is evidenced by the significantly improved positive predictive value (67.36%) for the non-singleton model (table 3). Previous machine learning models appear to show better prediction as they included non-singleton, gestational age (which is in terms of temporal association highly associated with LBW but occurs at the same time as the LBW can be measured) and preeclampsia in third trimester. Also, the differences in the proportion of LBW cases, the variables used, and the cohort sizes in various other studies alter the ability of the model, hence direct comparison of machine learning models across studies can become difficult.

The strength of this study lies in using a wide spectrum of routinely collected nationally representative administrative datasets of all births in Wales across a large time. This is a very first of its kind study in Wales and adds novelty in the research filed of LBW. However, this work can only identify the more severe cases which are recorded in the health care system, and undiagnosed cases that did not result in the system will be missed which is a limitation of this work. Since the study was developed on the linked routine data, the limitation of the routine data was encountered in this study, for e.g., though

For peer review only - http://bmjopen.bmj.com/site/about/guidelines.xhtml

the maternal weight variable came from two different sources, data was missing for many participants which was addressed by imputation methods. Also, this study was unable to capture lifestyle factors (diet, physical activity, stress, emotional state) which can be important in determining LBW [42,43].

The two different models (MLR and DT) used in this study has very similar findings suggesting that factors which are common and so are predictive (using DT methods) such as maternal smoking status and low maternal weight could be targeted to address population-level risk of LBW. Factors which have a strong association with LBW (using regression analysis), such as a mother with diabetes or mother on antidepressants as having plausible causal link to LBW, can be addressed to reduce individual risk for that mother/child.

Conclusion

This study suggests that the most important factors to reduce the risk of LBW are to address multiple birth (e.g. in assisted reproduction practices), addressing factors associated with preterm births (previous history of preterm birth), addressing maternal health such as reducing smoking, investment in maternal mental health, addressing substance use (alcohol/drugs), treating underlying health conditions (diabetes/anaemia), and promoting planning of pregnancy to give an adequate pregnancy interval and healthy weight of mother especially for those in deprived urban areas.

Acknowledgement

Funding

This work was funded by Public Health Wales (PHW), grant number (105186). This work was supported by Nation Institute for Health Research (NIHR), grant number (NIHR133680). This research has been carried out as part of the ADR Wales programme of work. The ADR Wales programme of work is aligned to the priority themes as identified in the Welsh Government's national strategy: Prosperity for All. ADR Wales brings together data science experts at Swansea University Medical School, staff from the Wales Institute of Social and Economic Research, Data and Methods (WISERD) at Cardiff University and specialist teams within the Welsh Government to develop new evidence which supports Prosperity for All by using the SAIL Databank at Swansea University, to link and analyse anonymised data. ADR Wales is part of the Economic and Social Research Council (part of UK Research and Innovation) funded ADR UK (grant ES/S007393/1).

This work was also supported by the National Centre for Population Health and Well-Being Research (NCPHWR) which is funded by Health and Care Research Wales. This work was supported by Health

For peer review only - http://bmjopen.bmj.com/site/about/guidelines.xhtml

Data Research UK which receives its funding from HDR UK Ltd (NIWA1) funded by the UK Medical Research Council, Engineering and Physical Sciences Research Council, Economic and Social Research Council, Department of Health and Social Care (England), Chief Scientist Office of the Scottish Government Health and Social Care Directorates, Health and Social Care Research and Development Division (Welsh Government), Public Health Agency (Northern Ireland), British Heart Foundation (BHF) and the Welcome Trust.

This work uses data provided by patients and collected by the NHS as part of their care and support. This study used anonymised data held in the Secure Anonymised Information Linkage (SAIL) Databank. We would like to acknowledge all the data providers who enable SAIL to make anonymised data available for research. We acknowledge the support provided by South Wales Police.

The work conducted does not represent or is it endorsed by the Office for National Statistics

Contributorship statement

Planning- Conceptualization: Sinead Brophy, Angela Jones and Julie Evans and Amrita Bandyopadhyay; Data acquisition: The police PPN and MID data accusation was supported by Ben Rowe and Julie Evans respectively. The other health data was available in SAIL, obtained through IGRP request lead by Sinead Brophy and Amrita Bandyopadhyay; Supervision: Sinead Brophy.

Conduct- Literature Review: Charlotte Todd and Emily Marchant; Methodology: Amrita Bandyopadhyay and Sinead Brophy; Data preparation: Muhammad A. Rahman and Amrita Bandyopadhyay, Formal analysis, and investigation: Amrita Bandyopadhyay; Additional support in analysis: James Healy and Michael Parker.

Writing- Original draft preparation: Amrita Bandyopadhyay; Review and editing: Charlotte Todd, Michael Parker, Julie Evans, Emily Marchant, Hope Jones, Muhammad A. Rahman, James Healy, Tint Lwin Win, Ben Rowe, Simon Moore, Angela Jones, and Sinead Brophy. All authors read and approved the final manuscript.

Competing interest’s statement

The authors declare that they have no conflict of interest.

The views expressed in this paper are those of the authors and not necessarily those of the Office for National Statistics

For peer review only

Participant consent

The study did not require participant consent as it utilises the anonymised data.

Ethical approval

No human participants were included.

The original protocol

Not applicable

STROBE checklist

STROBE checklist has been added as a Supplementary file (Supplementary material STROBE checklist).

Data sharing statement

The data have been archived in the Secure Anonymised Information Linkage Databank (https://saildatabank.com/0029)

References

1 WHO | Global Nutrition Targets 2025: Low birth weight policy brief. WHO. http://www.who.int/nutrition/publications/globaltargets2025_policybrief_lbw/en/ (accessed 19 Nov 2020).

2 UNICEF-WHO Low birthweight estimates: Levels and trends 2000–2015. https://www.unicef.org/reports/UNICEF-WHO-low-birthweight-estimates-2019 (accessed 3 Mar 2022).

3 Johnson CD, Jones S, Paranjothy S. Reducing low birth weight: prioritizing action to address modifiable risk factors. *J Public Health Oxf Engl* 2017;**39**:122–31. doi:10.1093/pubmed/fdv212

4 Mohammed SG. Low birth weight in Omdurman maternity hospital. *Int J Sci Res Publ* 2014;**4**:1–13.

5 Breslau N, Paneth NS, Lucia VC. The Lingering Academic Deficits of Low Birth Weight Children. *Pediatrics* 2004;**114**:1035–40. doi:10.1542/peds.2004-0069

6 Ohlsson A, Shah P. *Determinants and prevention of low birth weight: a synopsis of the evidence*. Institute of Health Economics 2008.

7 Heaman MI, Sprague AE, Stewart PJ. Reducing the Preterm Birth Rate: A Population Health Strategy. *J Obstet Gynecol Neonatal Nurs* 2001;**30**:20–9. doi:https://doi.org/10.1111/j.1552-6909.2001.tb01518.x

For peer review only - http://bmjopen.bmj.com/site/about/guidelines.xhtml

8 Yuan W, Duffner AM, Chen L, *et al.* Analysis of preterm deliveries below 35 weeks' gestation in a tertiary referral hospital in the UK. A case-control survey. *BMC Res Notes* 2010;**3**:119. doi:10.1186/1756-0500-3-119

9 Blencowe H, Cousens S, Chou D, *et al.* Born Too Soon: The global epidemiology of 15 million preterm births. *Reprod Health* 2013;**10**:S2. doi:10.1186/1742-4755-10-S1-S2

10 Blencowe H, Krasevec J, Onis M de, *et al.* National, regional, and worldwide estimates of low birthweight in 2015, with trends from 2000: a systematic analysis. *Lancet Glob Health* 2019;**7**:e849–60. doi:10.1016/S2214-109X(18)30565-5

11 Shi L, Macinko J, Starfield B, *et al.* Primary care, infant mortality, and low birth weight in the states of the USA. *J Epidemiol Community Health* 2004;**58**:374–80. doi:10.1136/jech.2003.013078

12 Silvestrin S, Silva CH da, Hirakata VN, *et al.* Maternal education level and low birth weight: a meta-analysis. *J Pediatr (Rio J)* 2013;**89**:339–45. doi:10.1016/j.jped.2013.01.003

13 Conde-Agudelo A, Rosas-Bermúdez A, Kafury-Goeta AC. Birth Spacing and Risk of Adverse Perinatal Outcomes: A Meta-analysis. *JAMA* 2006;**295**:1809–23. doi:10.1001/jama.295.15.1809

14 Yu Z, Han S, Zhu J, *et al.* Pre-Pregnancy Body Mass Index in Relation to Infant Birth Weight and Offspring Overweight/Obesity: A Systematic Review and Meta-Analysis. *PLOS ONE* 2013;**8**:e61627. doi:10.1371/journal.pone.0061627

15 Daalderop LA, Wieland BV, Tomsin K, *et al.* Periodontal Disease and Pregnancy Outcomes: Overview of Systematic Reviews. *JDR Clin Transl Res* 2018;**3**:10–27. doi:10.1177/2380084417731097

16 Flynn CA, Helwig AL, Meurer LN. Bacterial vaginosis in pregnancy and the risk of prematurity. *J Fam Pract* 1999;**48**:885–92.

17 Figueiredo ACMG, Gomes-Filho IS, Silva RB, *et al.* Maternal Anemia and Low Birth Weight: A Systematic Review and Meta-Analysis. *Nutrients* 2018;**10**. doi:10.3390/nu10050601

18 Dadi AF, Miller ER, Bisetegn TA, *et al.* Global burden of antenatal depression and its association with adverse birth outcomes: an umbrella review. *BMC Public Health* 2020;**20**:173. doi:10.1186/s12889-020-8293-9

19 Lima SAM, Dib RPE, Rodrigues MRK, *et al.* Is the risk of low birth weight or preterm labor greater when maternal stress is experienced during pregnancy? A systematic review and meta-analysis of cohort studies. *PLOS ONE* 2018;**13**:e0200594. doi:10.1371/journal.pone.0200594

20 Fleischer Nancy L., Merialdi Mario, van Donkelaar Aaron, *et al.* Outdoor Air Pollution, Preterm Birth, and Low Birth Weight: Analysis of the World Health Organization Global Survey on Maternal and Perinatal Health. *Environ Health Perspect* 2014;**122**:425–30. doi:10.1289/ehp.1306837

21 Flower A, Shawe J, Stephenson J, *et al.* Pregnancy planning, smoking behaviour during pregnancy, and neonatal outcome: UK millennium cohort study. *BMC Pregnancy Childbirth* 2013;**13**:238. doi:10.1186/1471-2393-13-238

For peer review only - http://bmjopen.bmj.com/site/about/guidelines.xhtml

22 Jaddoe VWV, Troe E-JWM, Hofman A, *et al.* Active and passive maternal smoking during pregnancy and the risks of low birthweight and preterm birth: the Generation R Study. *Paediatr Perinat Epidemiol* 2008;**22**:162–71. doi:10.1111/j.1365-3016.2007.00916.x

23 Patra J, Bakker R, Irving H, *et al.* Dose–response relationship between alcohol consumption before and during pregnancy and the risks of low birthweight, preterm birth and small for gestational age (SGA)—a systematic review and meta-analyses. *BJOG Int J Obstet Gynaecol* 2011;**118**:1411–21. doi:10.1111/j.1471-0528.2011.03050.x

24 dos Santos JF, de Melo Bastos Cavalcante C, Barbosa FT, *et al.* Maternal, fetal and neonatal consequences associated with the use of crack cocaine during the gestational period: a systematic review and meta-analysis. *Arch Gynecol Obstet* 2018;**298**:487–503. doi:10.1007/s00404-018-4833-2

25 Hill A, Pallitto C, McCleary-Sills J, *et al.* A systematic review and meta-analysis of intimate partner violence during pregnancy and selected birth outcomes. *Int J Gynaecol Obstet Off Organ Int Fed Gynaecol Obstet* 2016;**133**:269–76. doi:10.1016/j.ijgo.2015.10.023

26 Donovan BM, Spracklen CN, Schweizer ML, *et al.* Intimate partner violence during pregnancy and the risk for adverse infant outcomes: a systematic review and meta-analysis. *BJOG Int J Obstet Gynaecol* 2016;**123**:1289–99. doi:https://doi.org/10.1111/1471-0528.13928

27 Lyons RA, Jones KH, John G, *et al.* The SAIL databank: linking multiple health and social care datasets. *BMC Med Inform Decis Mak* 2009;**9**:3. doi:10.1186/1472-6947-9-3

28 Constabulary © Her Majesty's Inspectorate of, Fire. Police effectiveness 2015 (Vulnerability) – Dyfed-Powys Police. HMICFRS. https://www.justiceinspectorates.gov.uk/hmicfrs/publications/police-effectiveness-vulnerability-2015-dyfed-powys/ (accessed 3 Nov 2021).

29 Gelman A, Hill J. *Data analysis using regression and multilevel/hierarchical models*. Cambridge university press 2006.

30 Lewis RJ. An introduction to classification and regression tree (CART) analysis. In: *Annual meeting of the society for academic emergency medicine in San Francisco, California*. 2000.

31 Atkinson Beth. rpart function | R Documentation. https://www.rdocumentation.org/packages/rpart/versions/4.1-15/topics/rpart (accessed 14 Jan 2021).

32 ONS. Birth characteristics in England and Wales - Office for National Statistics. 2019.https://www.ons.gov.uk/peoplepopulationandcommunity/birthsdeathsandmarriages/livebirths/bulletins/birthcharacteristicsinenglandandwales/2017 (accessed 14 Jul 2021).

33 Regan AK, Ball SJ, Warren JL, *et al.* A Population-Based Matched-Sibling Analysis Estimating the Associations Between First Interpregnancy Interval and Birth Outcomes. *Am J Epidemiol* 2019;**188**:9–16. doi:10.1093/aje/kwy188

34 Class QA, Rickert ME, Oberg AS, *et al.* Within-Family Analysis of Interpregnancy Interval and Adverse Birth Outcomes. *Obstet Gynecol* 2017;**130**:1304–11. doi:10.1097/AOG.0000000000002358

For peer review only - http://bmjopen.bmj.com/site/about/guidelines.xhtml

35 Hanley GE, Hutcheon JA, Kinniburgh BA, *et al.* Interpregnancy Interval and Adverse Pregnancy Outcomes: An Analysis of Successive Pregnancies. *Obstet Gynecol* 2017;**129**:408–15. doi:10.1097/AOG.0000000000001891

36 Hutcheon JA, Nelson HD, Stidd R, *et al.* Short interpregnancy intervals and adverse maternal outcomes in high-resource settings: An updated systematic review. *Paediatr Perinat Epidemiol* 2019;**33**:O48–59. doi:10.1111/ppe.12518

37 Chamberlain C, O'Mara-Eves A, Porter J, *et al.* Psychosocial interventions for supporting women to stop smoking in pregnancy. *Cochrane Database Syst Rev* Published Online First: 2017. doi:10.1002/14651858.CD001055.pub5

38 Howard LM, Khalifeh H. Perinatal mental health: a review of progress and challenges. *World Psychiatry* 2020;**19**:313–27. doi:10.1002/wps.20769

39 Faruk A, Cahyono ES. Prediction and Classification of Low Birth Weight Data Using Machine Learning Techniques. *Indones J Sci Technol* 2018;**3**:18–28. doi:10.17509/ijost.v3i1.10799

40 Loreto P, Peixoto H, Abelha A, *et al.* Predicting Low Birth Weight Babies Through Data Mining. In: Rocha Á, Adeli H, Reis LP, *et al.*, eds. *New Knowledge in Information Systems and Technologies*. Cham: : Springer International Publishing 2019. 568–77. doi:10.1007/978-3-030-16187-3_55

41 Ahmadi P, Alavimajd H, Khodakarim S, *et al.* Prediction of low birth weight using Random Forest: A comparison with Logistic Regression. *Arch Adv Biosci* 2017;**8**:36–43.

42 Ghavi A, Fadakar Sogheh K, Niknamy M, *et al.* Investigating the Relationship between Maternal Lifestyle during Pregnancy and Low-Birth-Weight of Term Neonates. *Iran J Obstet Gynecol Infertil* 2012;**15**:14–24.http://ijogi.mums.ac.ir/article_58.html (accessed 7 Mar 2022).

43 Xi C, Luo M, Wang T, *et al.* Association between maternal lifestyle factors and low birth weight in preterm and term births: a case-control study. *Reprod Health* 2020;**17**:93. doi:10.1186/s12978-020-00932-9

Figure Legend

Figure 1 Participants flow diagram.

Figure 2: Significant factors associated with the risk LBW among the overall study population.

For peer review only

For peer review only - http://bmjopen.bmj.com/site/about/guidelines.xhtml

Figure 1 Participants flow diagram.

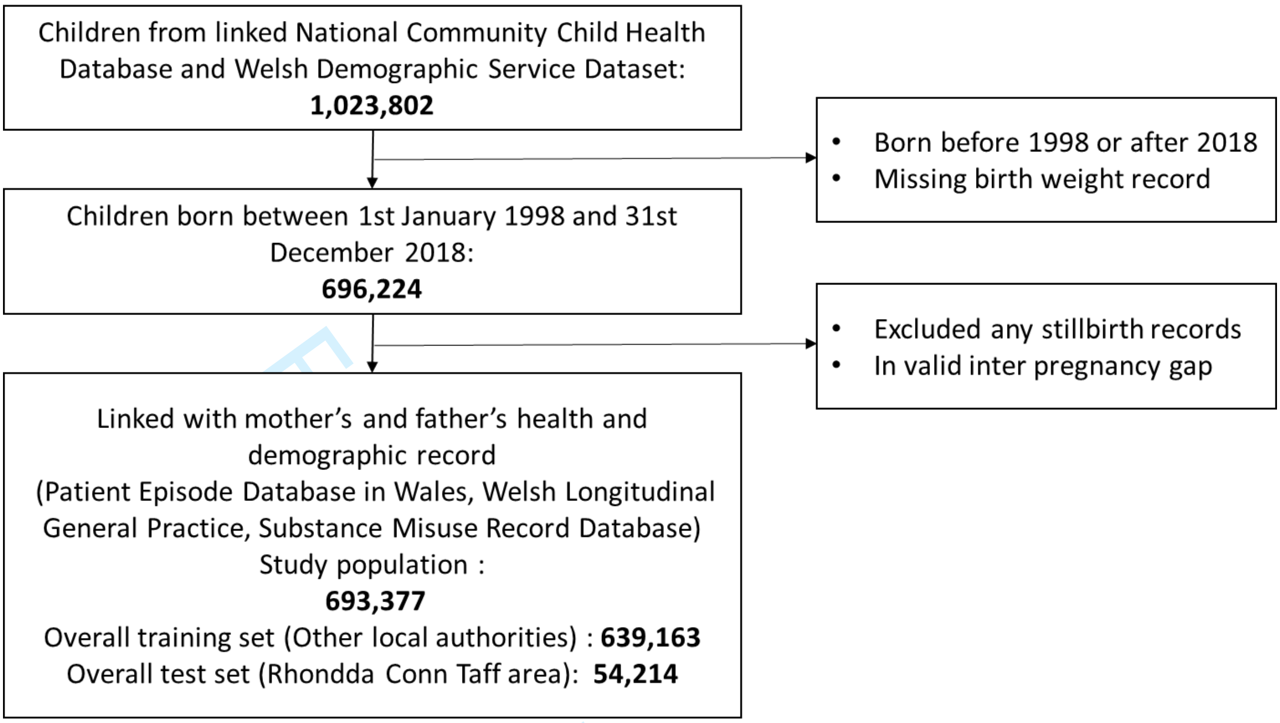

Figure 2: Significant factors associated with the risk LBW among the overall study population.

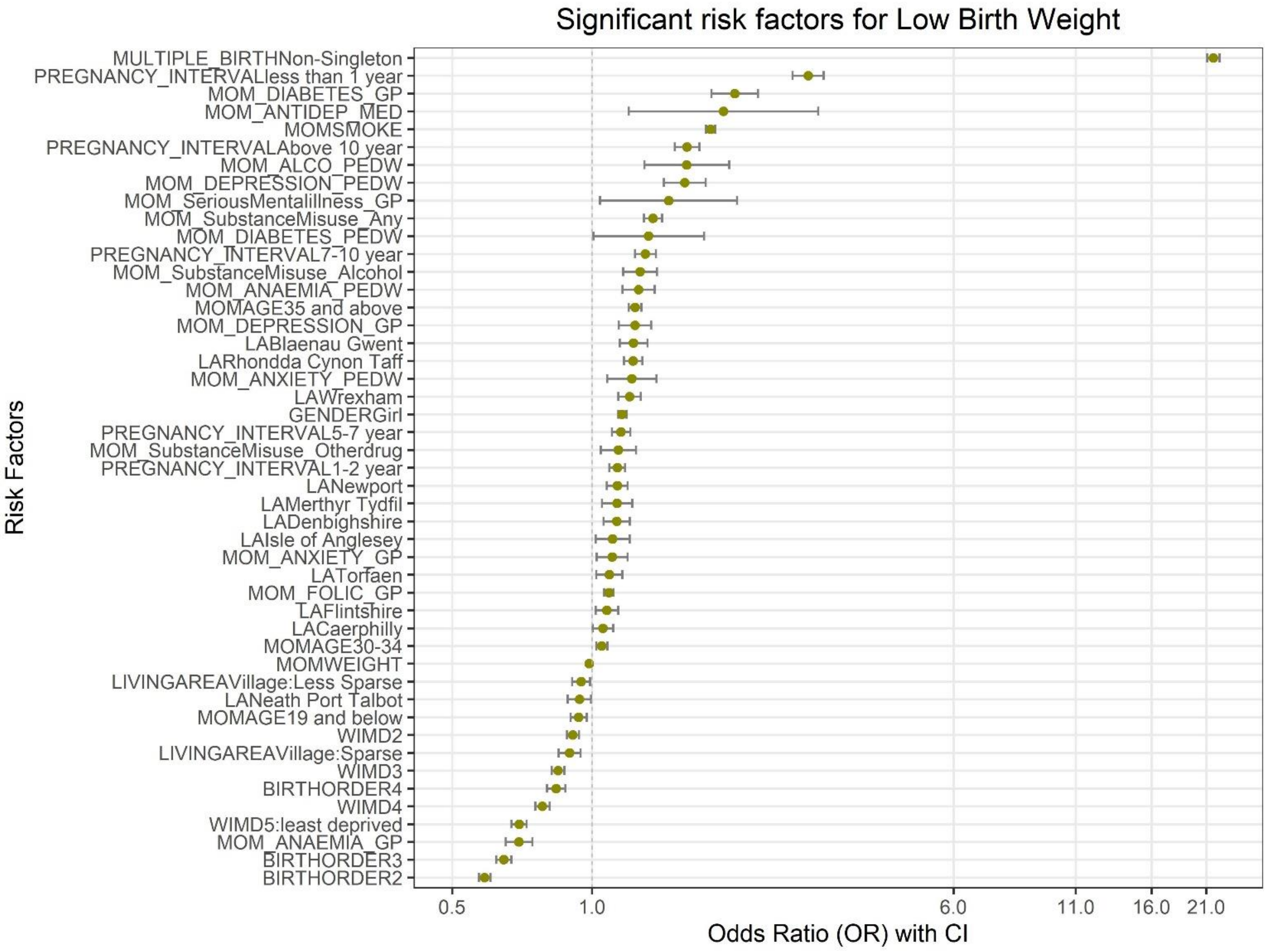

w only

Supplementary Figure 1: Significant risk factors associated with the risk LBW after linking with PPN record

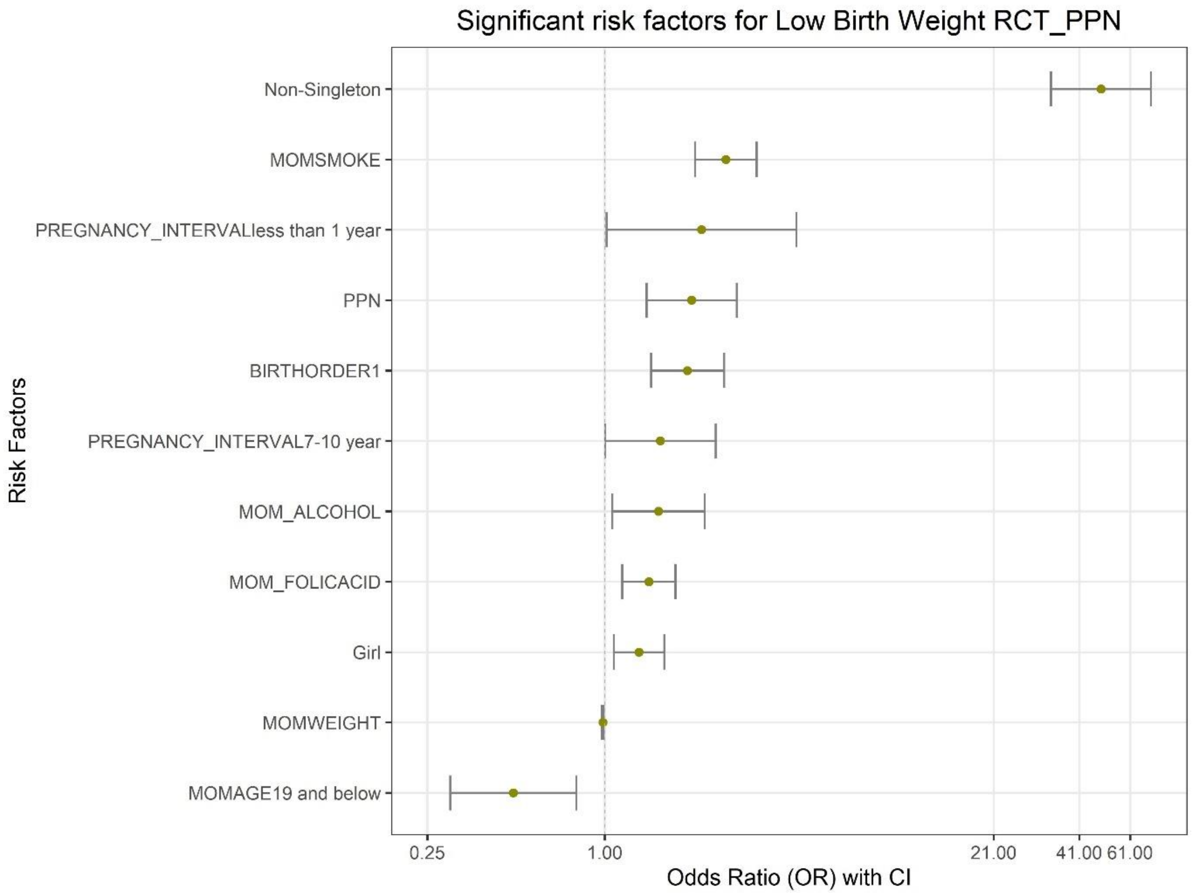

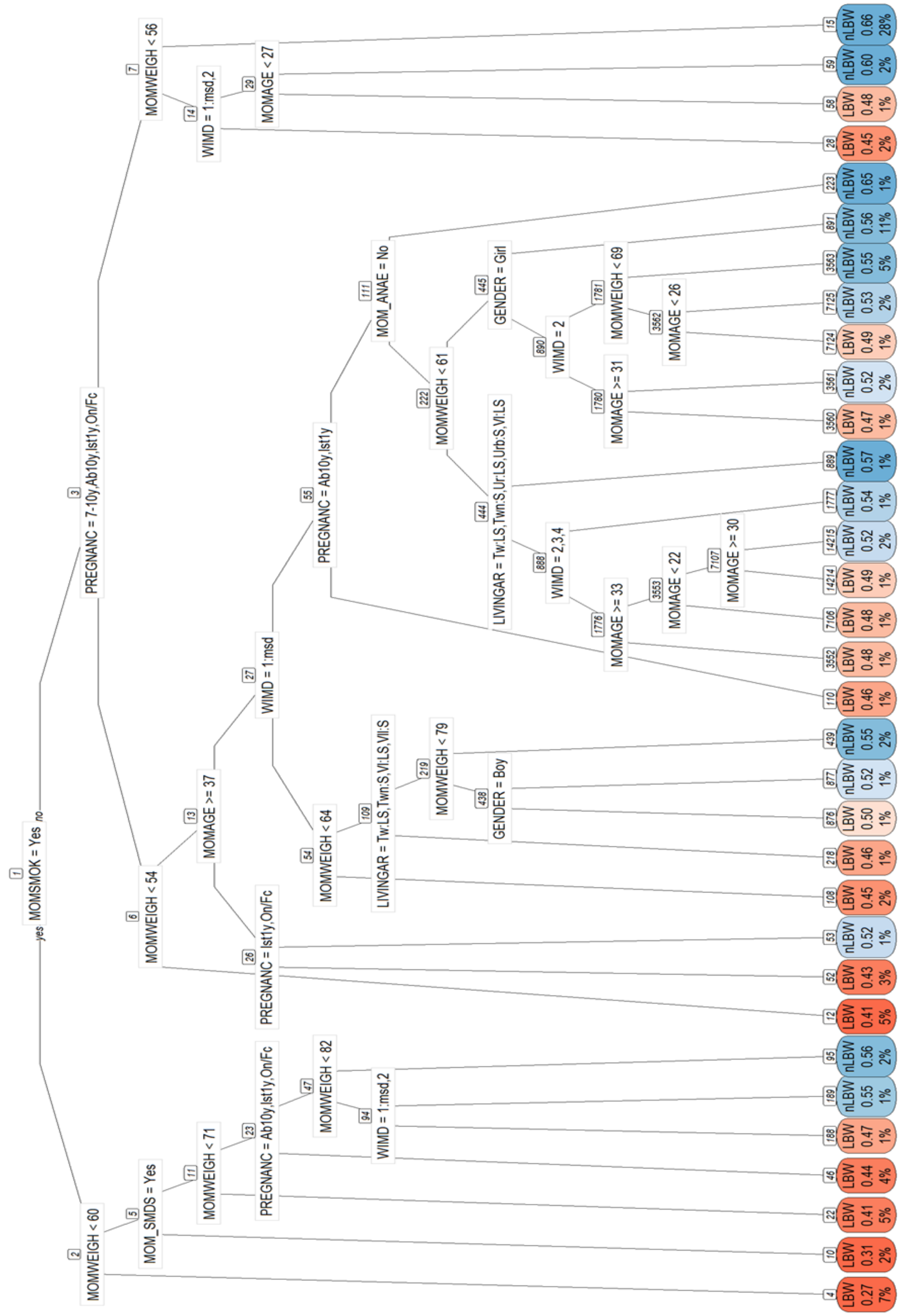

Supplementary Figure 2: Decision tree for singleton children

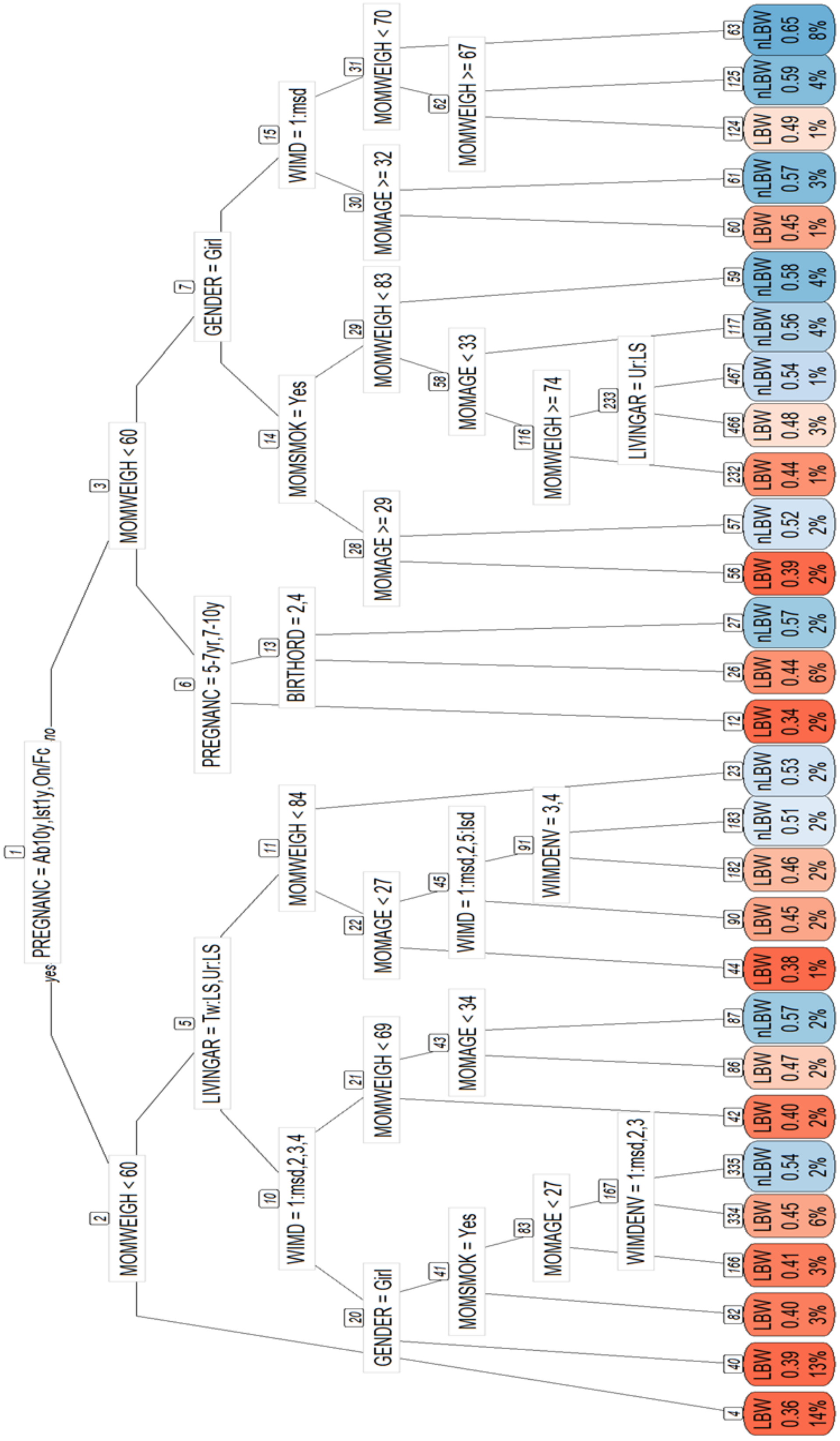

Supplementary Figure 3: Decision tree for non-singleton children

Supplementary Table1: Variables and their source datasets

Variables	NCCHD	Description
WOB	Welsh Demographic Service (WDS) dataset	Week of birth, the first Monday of the birth week
Gender	National Community Child Health Database (NCCHD)	Sex of the child
Maternal age	NCCHD	Maternal age at child’s birth
Gestational age	NCCHD	Gestational age in week (between 22 and 45 weeks)
Birth weight	NCCHD	Birth weight in gm (max 5000 gm)
Birth order	Derived	It’s based on the order of the child in the family using their week of birth and DENSE_RANK function. It ranks the children same if they are non-singleton children and sharing same WOB.
Pregnancy interval	Derived	Pregnancy interval, in week format, was derived using the birth order, week of birth (the Monday of the week of date of birth), of the previous child and the current child, maternal identifier, and the multiple birth flag.
Multiple birth flag	Derived	Using WOB, encrypted maternal identifier and the birth order, a binary variable – ‘multiple birth flag’ was derived to distinguish between singleton and non-singleton birth.
Mother weight (kg)	Derived	The maternal weight during pregnancy was obtained from MID and WLGP. The final maternal weight variable was derived following cleaning and harmonising it with the source variables which includes removing and recoding missing, erroneous, and inconsistent records.
Maternal smoking	NCCHD, Maternity Indicators Dataset (MIDS), Welsh Longitudinal General Practice Dataset (WLGP) - Welsh Primary Care	A cleaned and harmonised variable of maternal smoking during pregnancy was created based on the data obtained from three sources.
WIMD	WDS	Welsh Index of Multiple Deprivation (1: most deprived; 5: least deprived)

For peer review only

Diabetes (GP)	WLGP	Mothers' diabetes record from GP during pregnancy
Diabetes (Hospital)	Patient Episode Dataset for Wales (PEDW)	Mothers' diabetes record from hospital during pregnancy
Depression (GP)	WLGP	Mothers' depression record from GP during pregnancy
Depression (Hospital)	PEDW	Mothers' depression record from hospital during pregnancy
Serious Mental Illness	WLGP	Mothers' serious mental illness related record from GP during pregnancy
Anxiety (GP)	WLGP	Mothers' anxiety record from GP during pregnancy
Anxiety (Hospital)	PEDW	Mothers' anxiety record from hospital during pregnancy
Anti-depressant medication	WLGP	Mothers' receiving anti-depressant medication from GP during pregnancy
Vitamin D	WLGP	Mothers' receiving Vitamin D from GP during pregnancy
FOLIC Acid	WLGP	Mothers' receiving Folic acid from GP during pregnancy
Anaemia (GP)	WLGP	Mothers' Anaemia record from GP during pregnancy
Anaemia (Hospital)	PEDW	Mothers' Anaemia record from hospital during pregnancy
Alcohol (GP)	WLGP	Mothers' alcohol record from GP during pregnancy
Anaemia (Hospital)	PEDW	Mothers' alcohol record from hospital during pregnancy
Assault	PEDW	Mother admitted to hospital during pregnancy for assault
Substance misuse	Substance Misuse Dataset (SMDS)	Mother receiving substance misuse treatment during pregnancy from SMD database
Living area	WDS	Living area during child's birth
Local authority	WDS	Local authority of the living area

For peer review only

Supplementary Table 2: Characteristics of the study population

Variables	Overall training set (n = 639,163)		Overall test set (n = 54,214)		Overall (n = 693,377)	
Gender						
Girl	311,193	48.69%	26,689	49.23%	337,882	48.73%
Boy	327,920	51.30%	27,522	50.77%	355,442	51.26%
Unknown/NULL	50	0.01%	<5	-	-	-
Maternal age						
Less than 19	46,668	7.30%	5,156	9.51%	51,824	7.47%
20-24	133,792	20.93%	13,149	24.25%	146,941	21.19%
25-29	181,233	28.35%	16,454	30.35%	197,687	28.51%
30-34	170,957	26.75%	12,938	23.86%	183,895	26.52%
35 and above	105,869	16.56%	6,513	12.01%	112,382	16.21%
Unknown/NULL	644	0.10%	<5	-	-	-
Birth order						
1st child	319,093	49.92%	26,552	48.98%	345,645	49.85%
2nd child	212,155	33.19%	18,672	34.44%	230,827	33.29%
3rd child	74,724	11.69%	6,407	11.82%	81,131	11.70%
4th or above	33,191	5.19%	2,583	4.76%	35,774	5.16%
Pregnancy interval						
Only/First child	319,093	49.92%	26,552	48.98%	345,645	49.85%
less than 1 year	5,708	0.89%	526	0.97%	6,234	0.90%
1-2 years	67,986	10.64%	5,333	9.84%	73,319	10.57%
2-5 years	162,590	25.44%	13,519	24.94%	176,109	25.40%
5-7 years	41,060	6.42%	4,081	7.53%	45,141	6.51%
7-10 years	27,161	4.25%	2,707	4.99%	29,868	4.31%
Above 10 years	15,565	2.44%	1,496	2.76%	17,061	2.46%
Gestational age (week)						
1: Extremely pre-term: <28 week	2,361	0.37%	208	0.38%	2,569	0.37%
2: Very pre-term: 28-31	5,296	0.83%	565	1.04%	5,861	0.85%
3: Pre-term: 32-36	38,565	6.03%	3,664	6.76%	42,229	6.09%
4: term: 37-42	577,104	90.29%	49,540	91.38%	626,644	90.38%
5: Late term: 43-45	3,909	0.61%	91	0.17%	4,000	0.58%
Unknown/NULL	11,928	1.87%	146	0.27%	12,074	1.74%
Birth weight (gm)						
1: BW≤ 1,000	3,010	0.47%	246	0.45%	3,256	0.47%
2: BW 1,001 - 1,500	4,372	0.68%	445	0.82%	4,817	0.69%
3: BW 1,501 - 2,499	39,143	6.12%	3,923	7.24%	43,066	6.21%
4: BW 2,500 - 4,000	521,172	81.54%	44,524	82.13%	565,696	81.59%
5: BW 4,001 - 4,500	61,658	9.65%	4,413	8.14%	66,071	9.53%
6: BW 4,501 - 5000	9,808	1.53%	663	1.22%	10,471	1.51%
Low Birth Weight (LBW)						
nLBW	594,408	93.00%	49,734	91.74%	644,142	92.90%
LBW	44,755	7.00%	4,480	8.26%	49,235	7.10%

For peer review only

Multiple birth flag						
Singleton	619,458	96.92%	52,583	96.99%	672,041	96.92%
Non-singleton	19,705	3.08%	1,631	3.01%	21,336	3.08%
Maternal smoking						
No	502,914	78.68%	41,344	76.26%	544,258	78.49%
Yes	136,249	21.32%	12,870	23.74%	149,119	21.51%
Welsh Index of Multiple Deprivation						
1 (most deprived)	147,204	23.03%	17,946	33.10%	165,150	23.82%
2	118,271	18.50%	17,711	32.67%	135,982	19.61%
3	117,242	18.34%	7,089	13.08%	124,331	17.93%
4	104,056	16.28%	3,646	6.73%	107,702	15.53%
5 (least deprived)	94,190	14.74%	6,242	11.51%	100,432	14.48%
Unknown/NULL	58,200	9.11%	1,580	2.91%	59,780	8.62%
Diabetes GP (mother)						
No	638,628	99.92%	54,149	99.88%	692,777	99.91%
Yes	535	0.08%	65	0.12%	600	0.09%
Diabetes PEDW (mother)						
No	636,104	99.52%	53,978	99.56%	690,082	99.52%
Yes	3,059	0.48%	236	0.44%	3,295	0.48%
Depression GP (mother)						
No	631,230	98.76%	53,323	98.36%	684,553	98.73%
Yes	7,933	1.24%	891	1.64%	8,824	1.27%
Depression PEDW (mother)						
No	634,990	99.35%	53,950	99.51%	688,940	99.36%
Yes	4,173	0.65%	264	0.49%	4,437	0.64%
Serious Mental Illness (mother)						
No	638,887	99.96%	54,185	99.95%	693,072	99.96%
Yes	276	0.04%	29	0.05%	305	0.04%
Anxiety GP (mother)						
No	629,681	98.52%	53,131	98.00%	682,812	98.48%
Yes	9,482	1.48%	1,083	2.00%	10,565	1.52%
Anxiety PEDW (mother)						
No	635,910	99.49%	53,967	99.54%	689,877	99.50%
Yes	3,253	0.51%	247	0.46%	3,500	0.50%
Anti-depressant medication (mother)						
No	639,019	99.98%	54,194	99.96%	693,213	99.98%
Yes	144	0.02%	20	0.04%	164	0.02%
Vitamin D (mother)						
No	637,171	99.69%	54,170	99.92%	691,341	99.71%
Yes	1,992	0.31%	44	0.08%	2,036	0.29%
FOLIC Acid (mother)						
No	486,360	76.09%	37,663	69.47%	524,023	75.58%
Yes	152,803	23.91%	16,551	30.53%	169,354	24.42%

For peer review only

Anaemia GP (mother)						
No	621,276	97.20%	52,636	97.09%	673,912	97.19%
Yes	17,887	2.80%	1,578	2.91%	19,465	2.81%
Anaemia PEDW (mother)						
No	631,370	98.78%	53,883	99.39%	685,253	98.83%
Yes	7,793	1.22%	331	0.61%	8,124	1.17%
Alcohol - GP (mother)						
No	601,660	94.13%	50,836	93.77%	652,496	94.10%
Yes	37,503	5.87%	3,378	6.23%	40,881	5.90%
Alcohol - PEDW (mother)						
No	638532	99.90%	54177	99.93%	692,709	99.90%
Yes	631	0.10%	37	0.07%	668	0.10%
Assault - PEDW (mother)						
No	638,479	99.89%	54165	99.91%	692,644	99.89%
Yes	684	0.11%	49	0.09%	733	0.11%
Substance misuse – any (mother)						
No	607,433	95.04%	51,087	94.23%	658,520	94.97%
Yes	31,730	4.96%	3,127	5.77%	34,857	5.03%
Substance misuse - alcohol (mother)						
No	631,148	98.75%	53,235	98.19%	684,383	98.70%
Yes	7,767	1.22%	975	1.80%	8,742	1.26%
Unknown/NULL	248	0.04%	<5	-	-	-
Substance misuse - other (mother)						
No	632,443	98.95%	53,381	98.46%	685,824	98.91%
Yes	6,199	0.97%	794	1.46%	6,993	1.01%
Unknown/NULL	521	0.08%	39	0.07%	560	0.08%
Mother weight (kg)						
Average (before imputation)	71		72.39		71.06	
Median (before imputation)					67.58	
Average (after imputation)					70.82	
Median (after imputation)					67.00	
Living area						
Town and Fringe - Less Sparse	82,435	12.90%	12,702	23.43%	95,137	13.72%
Town and Fringe - Sparse	22,106	3.46%	71	0.13%	22,177	3.20%
Urban > 10K - Less Sparse	403,591	63.14%	37,924	69.95%	441,515	63.68%
Urban > 10K - Sparse	13,441	2.10%	37	0.07%	13,478	1.94%
Village, Hamlet & Isolated Dwellings - Less Sparse	46,166	7.22%	1,464	2.70%	47,630	6.87%
Village, Hamlet & Isolated Dwellings - Sparse	48,314	7.56%	169	0.31%	48,483	6.99%
Unknown/NULL	23,110	3.62%	1,847	3.41%	24,957	3.60%
Local authority						
Blaenau Gwent					15,008	2.16%
Bridgend					28,018	4.04%
Caerphilly					40,418	5.83%

For peer review only

Cardiff					80,247	11.57%
Carmarthenshire					34,705	5.01%
Ceredigion					11,090	1.60%
Conwy					20,389	2.94%
Denbighshire					19,697	2.84%
Flintshire					32,471	4.68%
Gwynedd					23,249	3.35%
Isle of Anglesey					13,941	2.01%
Merthyr Tydfil					13,259	1.91%
Monmouthshire					14,899	2.15%
Neath Port Talbot					28,854	4.16%
Newport					35,153	5.07%
Pembrokeshire					23,929	3.45%
Powys					20,546	2.96%
Rhondda Cynon Taff					54,214	7.82%
Swansea					49,588	7.15%
Torfaen					20,500	2.96%
Vale of Glamorgan					25,657	3.70%
Wrexham					29,346	4.23%
Unknown/NULL					58,199	8.39%

For peer review only

Supplementary Table 3: Multivariable logistic regression model to identify the risk factors of LBW among the overall study population.

Variable name in model (description)	OR	Lower CI	Upper CI
GENDER (Gender)			
Boy	1		
Girl	1.16	1.14	1.18
MOMSMOKE (Maternal smoking)			
No	1		
Yes	1.80	1.76	1.84
MOMAGE (Maternal age)			
Less than 19	0.94	0.90	0.97
20-24	1.00	0.97	1.03
25-29	1		
30-34	1.05	1.02	1.09
35 and above	1.24	1.20	1.29
BIRTHORDER (Birth order)			
1st child	1		
2nd child	0.59	0.57	0.60
3rd child	0.65	0.62	0.67
4th or above	0.84	0.80	0.88
PREGNANCY_INTERVAL (Pregnancy interval)			
Less than 1 year	2.92	2.70	3.15
1-2 years	1.13	1.09	1.18
2-5 years	1		
5-7 years	1.15	1.10	1.21
7-10 years	1.30	1.24	1.37
Above 10 years	1.60	1.51	1.70
MULTIPLE_BIRTH (Multiple birth flag)			
Singleton			
Non-singleton	21.74	21.09	22.40
WIMD (Welsh Index of Multiple Deprivation)			
1 (most deprived)	1		
2	0.91	0.88	0.94
3	0.84	0.82	0.87
4	0.78	0.75	0.81
5 (least deprived)	0.70	0.67	0.72
WIMDENV (Welsh Index of Multiple Deprivation – Environment score)			
1 (most deprived)	1		
2	0.99	0.96	1.02
3	1.01	0.98	1.04
4	1.02	0.99	1.06
5 (least deprived)	1.00	0.96	1.03
LA (Local authority)			
Blaenau Gwent	1.23	1.15	1.32

For peer review only

Bridgend	1.01	0.95	1.07
Caerphilly	1.06	1.00	1.11
Cardiff	1		
Carmarthenshire	1.02	0.96	1.08
Ceredigion	0.91	0.83	1.01
Conwy	1.05	0.98	1.12
Denbighshire	1.13	1.06	1.21
Flintshire	1.08	1.02	1.14
Gwynedd	1.04	0.97	1.12
Isle of Anglesey	1.11	1.02	1.20
Merthyr Tydfil	1.13	1.05	1.22
Monmouthshire	1.02	0.94	1.10
Neath Port Talbot	0.94	0.89	1.00
Newport	1.13	1.08	1.19
Pembrokeshire	1.04	0.97	1.11
Powys	1.01	0.93	1.09
Rhondda Cynon Taff	1.23	1.17	1.28
Swansea	0.97	0.93	1.02
Torfaen	1.09	1.02	1.16
Vale of Glamorgan	0.98	0.92	1.04
Wrexham	1.20	1.14	1.27
MOM_DIAB_GP (Diabetes GP (mother))			
No	1		
Yes	2.03	1.81	2.28
MOM_DIAB_PEDW (Diabetes PEDW (mother))			
No	1		
Yes	1.32	1.01	1.74
MOM_DEPRE_GP (Depression GP (mother))			
No	1		
Yes	1.24	1.14	1.34
MOM_DEPRE_PEDW (Depression PEDW (mother))			
No	1		
Yes	1.58	1.43	1.75
MOM_SeriousMentalillness_GP (Serious Mental Illness (mother))			
No	1		
Yes	1.46	1.04	2.05
MOM_VITD_GP (Vitamin D (mother))			
No	1		
Yes	1.15	0.96	1.38
MOM_FOLIC_GP			
No	1		
Yes	1.09	1.06	1.11
MOM_ALCO_GP (Alcohol - GP (mother))			
No	1		

For peer review only

Yes	1.02	0.98	1.06
MOM_ALCO_PEDW (Alcohol -PEDW (mother))			
No	1		
Yes	1.60	1.30	1.97
MOM_ANXIETY_GP (Anxiety GP (mother))			
No	1		
Yes	1.10	1.02	1.19
MOM_ANXIETY_PEDW (Anxiety PEDW (mother))			
No			
Yes	1.22	1.08	1.38
MOM_ANTIDEP_MED (Anti-depressant medication (mother))			
No	1		
Yes	1.92	1.20	3.07
MOM_ANAEMIA_GP (Anaemia GP (mother))			
No	1		
Yes	0.70	0.65	0.74
MOM_ANAEMIA_PEDW (Anaemia PEDW (mother))			
No	1		
Yes	1.26	1.16	1.36
MOM_ASSAULT (Assault - PEDW (mother))			
No	1		
Yes	1.16	0.91	1.47
MOM_SubstanceMisuse_Any (Substance misuse – any (mother))			
No	1		
Yes	1.35	1.29	1.41
MOM_SubstanceMisuse_Alcohol (Substance misuse - alcohol (mother))			
No	1		
Yes	1.27	1.17	1.38
MOM_SubstanceMisuse_Otherdrug (Substance misuse - other (mother))			
No	1		
Yes	1.14	1.04	1.24
MOMWEIGHT (Mother weight)			
	0.99	0.99	0.99
LIVINGAREA (Living area)			
Town and Fringe - Less Sparse	1.02	0.95	1.09
Town and Fringe - Sparse	1.03	0.96	1.12
Urban > 10K - Less Sparse	1		
Urban - Sparse	1.02	0.99	1.05
Village, Hamlet & Isolated Dwellings - Less Sparse	0.95	0.91	0.99
Village, Hamlet & Isolated Dwellings - Sparse	0.89	0.85	0.94

For peer review only

Supplementary Table 4: Distribution of LBW and nLBW children based on their multiple birth flags

	Overall training set (n = 639,163)		Overall test set (n = 54,214)		Total (n = 693,377)	
Singleton						
nLBW	585,163	94.46%	49,145	93.46%	634,308	94.39%
LBW	34,295	5.54%	3,438	6.54%	37,733	5.61%
Non-singleton						
nLBW	9,245	46.92%	589	36.11%	9,834	46.09%
LBW	10,460	53.08%	1,042	63.89%	11,502	53.91%

For peer review only

Weighting of risk factors for low birth weight

Aim

The WHO designates infants weighing 2500 g or less as 'low birth weight' (LBW). The main factors associated with LBW are due to intra-uterine growth restriction, or prematurity. This report examines:

(a) the risk factors associated with low birth weight and

(b) the relative weighting of importance of the risk factors in determining LBW. This includes

1. The strength of the association and
2. The number/prevalence of infants exposed to each risk factor in RCT and in Wales.
3. The number/prevalence of infants exposed to each risk factor at birth, in RCT flying start and non-flying start areas.

According to Welsh Government statistics 5.6% of singleton births were low birthweight in 2018.

Method

In order to examine factors associated with low birth weight (LBW), an initial scoping search was undertaken and a relevant piece of work by Johnson et al (2017) was identified, which was published in 2016 in collaboration with Public Health Wales. This piece of work aimed to understand the contribution of modifiable risk factors to the burden of LBW and identify prevalence data from the population of Wales. The study examined research from 2006-2013, but also reported on research prior to 2006 which was conducted by the Institute of Health Economics.

This current piece of work, commissioned by Public Health Wales, will build on the work by Johnson et al as a framework.

Search criteria: Firstly, any systematic reviews published since 2013 focusing on the risk factors identified in Johnson et al will be identified and the odds ratios of more recent studies conducted since their review will be noted in the table. Secondly, any systematic reviews published since 2010 will be explored for all additional risk factors not identified in Johnson et al. If systematic reviews cannot be found for these risk factors, a further search will be conducted to identify other types of study including cohort studies or case control studies.

Following this, a search will be conducted for prevalence of each risk factor. Where available, Welsh data will be reported. If welsh data is not found, then UK data will be presented, followed by evidence reviews or population cohort-based studies. Where pregnancy specific data is not able to be found, general population prevalence of each risk factor will be reported.

For peer review only - http://bmjopen.bmj.com/site/about/guidelines.xhtml

**Risk factor**	Risk range in research	Selected Risk size (OR or RR)	Evidence associated with presented OR/RR	Prevalence	Prevalence in RCT
Heroin/methadone	1.74-4.61	3.28	Hulse et al 1997 in Johnson et al: meta analyses	0.1% (CSEW 2018) General Population	
Cocaine	2.15-4.42	2.85<br><br>**2.80 (2.39-3.27)**	Moretti et al 2001 in Johnson et al: meta analyses<br>Dos Santos et al 2018: Systematic review crack cocaine use during pregnancy	2.6% powder cocaine and 0.1% powder cocaine-General population (CSEW 2018)	
Smoking in pregnancy	1.43-2.00	1.9<br><br>**2.0 (1.77-2.26)**<br><br>**1.91 (1.56-2.34)**	Walsh 1994.in Johnson et al<br><br>Pereira et al 2017: Systematic review and meta-analysis<br>Flower et al 2013: UK millennium cohort study	17.8% Public Health Wales (2017/18)<br><br>17.9% Welsh Government 2018	22.4% Welsh Government (Cwm Taf HB 2017/18)
Severe gum disease	1.5-1.8	1.8<br><br>**1.7 (1.3-2.1)**	Corbella et al 2012 in Johnson et al: systematic review and meta-analysis<br>Daalderop et al 2018: Overview of systematic reviews	40% (some degree of periodontal disease) Lieff 2004.<br><br>*Studies produce a wide variation in prevalence's (11% to 100%	
Cannabis	0.7-1.7	1.7<br><br>**1.77 (1.04-3.01)**	Hayatbakhsh et al 2012 in Johnson et al: cohort study<br>Gunn et al 2015: systematic review and meta analysis	7.2% (CSEW 2018) General population	
Low BMI	1.64-1.7	1.64	Han et al. 2011 in Johnson et al: cross sectional analyses	4.5% Underweight Public Health England (2019)	

For peer review only

Intimate partner violence	1.5-1.53<br><br>1.05-1.31<br><br>1.68-2.65	1.53<br><br>1.18<br><br>2.11	Shah et al 2011 in Johnson et al: Systematic review and meta analyses<br><br>Hill et al 2016: Systematic review and meta analysis<br><br>Donovan et al 2016: Systematic Review	5.7% CSEW 2019, adults experienced domestic abuse in the last year General Population	
Chlamydia	0.19-1.52	1.52<br><br>**1.34 (1.21-1.48)**	De Attayde Silva 2011<br><br>Olson-chen et al 2018	1.5% in women<br>3.1% in women aged 16-24 (Sonnenberg et al 2013) UK General Population<br><br>12% in pregnancy (Junghans et al 2016) UK	
Bacterial vaginosis	1.43-2.02	1.43	**Flynn et al 1999** in Johnson et al: meta analysis	7.1% (Desseauve et al 2012) in French pregnant population	
Anaemia	1.29-1.94	1.29<br><br>1.23 (1.06-1.43)	**Ref in Johnson et al** Haider et al 2013. : systematic review and meta-analysis<br><br>**Figuerido et al 2018:** Systematic review and meta-analysis	24% (Barroso et al 2011) UK Population<br><br>UK: 46% at booking or 28-week checks (Nair et al, 2017).	
Environmental tobacco smoke exposure	1.22-1.38	1.32	Bee et al 2008 in Johnson et al: systematic review and meta-analysis	Not available	
Teenage pregnancy	1.1-2.9	1.17	Haldre et al. 2007 in Johnson et al	2.9% (aged <20) ONS 2018	
Inter-pregnancy interval (1-5m)	1.06-3.54	1.61	Conde-Agudelo 2006 in Johnson et al : meta analysis	UK population cohort study	

For peer review only

Inter preg interval (6-11m)	1.06-3.54	1.14	Conde-Agudelo 2006 in Johnson et al : meta analysis	17.5% of women had interval pregnancy between 0-11 months on 1st and 2nd pregnancy and 19.7% on 2nd and 3rd pregnancy Ziauddeen et al (2019)	
Inter preg interal (12-18m)	1.06-3.54	1.06	Conde-Agudelo 2006 in Johnson et al : meta analysis		
Alcohol	0.64-1.27	1.06<br><br>2.0 (SGA)	Patra et al 2011 in Johnson et al: systematic review and meta analyses<br><br>Nykjaer et al 2014: British cohort	UK-41.3% (32.9-49) Popova et al (2017) Any alcohol use during pregnancy<br><br>Over 50% of women in a UK sample reported alcohol intakes in the first trimester above DH guidelines (<=2 units per week). Nykjaer et al 2014	
**Others:**					
Maternal anxiety during pregnancy		1.80 (1.48- 2.18)	Griogoriadis et al 2018: Systematic review and meta analysis	24.1% Welsh Government (2018): mental health condition reported at initial assessment	
Maternal Stress during/before pregnancy		1.68 (1.19-2.38)	Molina Lima et al 2018: Systematic review and meta analysis of cohort studies.		
Maternal depression during pregnancy		1.39 (1.22-1.58)	Dadi et al 2019: Umbrella review		

For peer review only

Antidepressant use in pregnancy		Limited effect (evidence issues)<br><br>1.44 (1.21-1.70)	Prady et al 2018: Systematic review<br><br>Huang 2014- Meta-analysis	4.5% SSRI prescribing during pregnancy in Wales (Charlton 2014)	
Pre-pregnancy BMI		Underweight and LBW<br>(1.47, 1.27-1.71)<br><br>Overweight and LBW: 0.79 to 1.01.<br>After publication bias accounted for: 0.95, 0.85-1.07).<br><br>Overweight and preterm birth<br>(1.24, 1.13 to 1.37)	Yu et al 2013: Systematic review and meta analysis<br><br>McDonald et al 2010: Systematic review and meta analysis.	28.0% Welsh Government (2018) : women obese (BMI 30+) at their initial assessment.	
Pregnancy weight gain		Low gestational weight gain and LBW<br>1.84 (1.71–1.99)<br><br>Low gestational weight gain and SGA<br>1.51 (1.39–1.63)	Han et al 2011<br><br>Goldstein et al 2018		

For peer review only

		High gestational weight gain and LBW<br>0.64 (0.53-0.78)	Mcdonald et al 2011		
Caffeine intake during pregnancy		Limited evidence<br><br>Low intake (50 to 149mg/day):<br>1.13 (1.06-1.21)<br><br>Moderate intake (150 to 349mg/day 1.38 (1.18-1.62)<br><br>High intake (>=350mg/day)<br>1.60 (1.24-2.08	Jahanfar et al 2015: Cochrane systematic review<br><br>Chen et al 2014: Systematic review and dose response meta-analysis.	No recent data available	
Area deprivation (neighbourhood and individual social class)		Area: 1.81 (1.71 - 1.92)<br>Social class (1.79 (1.43 to 2.24)<br><br>LBW (1.11, 1.02-1.20)<br><br>SGA: 1.31 (1.28-1.34)	Weightman et al 2012: UK specific systematic review<br><br>Metcalf et al 2011<br><br>Vos et al 2014		
Vitamin D supplementation in pregnancy	0.35-0.87<br><br>0.22-0.74	0.50<br><br>0.40 **	Palacios et al 2019: Cochrane review<br><br>Maugeri et al 2019 Systematic review of RCTs.		

For peer review only

			De Regil et al 2016: Cochrane review		
Folic acid supplementation		No conclusive evidence<br>RR 0.83, 0.66 - 1.04<br><br>No effect	Lassi et al 2013: cochrane systematic review<br><br>Lopes et al 2017: overview of systematic reviews	31% took folic acid prior to conception Bestwick et al 2014	
Air pollution		1.03–1.21	Guo et al 2019: systematic review and meta analysis		
Maternal education level		0.67 (0.51-0.88), High maternal education	Silvestrin et al 2013		
Maternal Age		Mixed findings	Goisis et al 2017: Finnish population data linkage study<br>Goisis et al 2018: UK cross cohort comparison study	Age 40+ 4% ONS 2018	
Paternal factors		Advance paternal age<br>Prolonged lead exposure and low paternal education may be associated	Shah et al 2010: Systematic review		

For peer review only

Summaries of above reported research:

Maternal depression during pregnancy

Dadi et al (2019): Global burden of antenatal depression and its association with adverse birth outcomes: an umbrella review

This umbrella review pooled estimates of three systematic reviews exploring the association between depression during pregnancy (measured using a validated screening or diagnostic tool) and LBW. Results showed that risk of LBW was 1.39 times higher among pregnant mothers with antenatal depression. Limitations of this review were that studies used different depression screening tools with different cut off values and there were different study designs among primary studies.

Antidepressant use during pregnancy:

Prady et al (2018): A systematic review of maternal antidepressant use in pregnancy and short- and long-term offspring's outcomes

This review evaluated the research which compared LBW and other outcomes for children whose mothers took antidepressants during pregnancy compared to those whose mothers had common mental disorders, or symptoms, but did not take anti-depressants during pregnancy. Four cohort studies were included with an outcome of LBW. Meta-analysis was unable to be conducted because of wide variation in study design and high risk of bias among studies. The authors concluded that there was little evidence to indicate that using antidepressants in pregnancy causes infants to have LBW (after adjusting for gestational age).

Authors stated limitations stemming from difficulty in being certain that any effects believed to be due to exposure to antidepressants did not reflect differences in social or clinical characteristics of women who continue antidepressants in pregnancy compared to those who discontinue or do not take them at all. They advocated for more consistency over how studies assess exposure variables, mental health disorders, outcomes and treatments.

An earlier review by Huang et al 2014 however found antidepressant use increased the risk of LBW and PTB but it involved a mixture of studies with different groups as controls and limited studies in the analysis controlled for severity and persistence of depression.

Maternal anxiety during pregnancy:

Grigoriadis et al (2018): Maternal Anxiety During Pregnancy and the Association With Adverse Perinatal Outcomes: Systematic Review and Meta-Analysis.

This systematic review and meta-analysis identified 11 studies using the outcome of LBW and showed the association with maternal anxiety was significant ($P < .00001$. Antenatal anxiety associated with increased odds of LBW, premature birth (1.54), and increased odds for small for gestational age (1.48). Studies which reported on clinical diagnosis of anxiety as their outcome produced a higher odds ratio (2.09) compared to studies using self-report measures (1.42) suggesting the severity of anxiety to be important in predicting low birth weight.

The limitations of this study relate to methodological issues of the primary research included in the review. The definition of anxiety by self-report varied across studies with the regards to the scales and cut off scores used. Even studies which used the State-Trait Anxiety Inventory (STAI), a commonly used measure of antenatal anxiety used different cut off scores. The review also included all types of anxiety disorders and was not specific as to particular disorders.

Maternal stress during pregnancy:

Molina Lima et al (2018): Is the risk of low birth weight or preterm labor greater when maternal stress is experienced during pregnancy? A systematic review and meta-analysis of cohort studies

This systematic review and meta-analysis included 8 cohort studies which proved eligible for inclusion in the review. Results of the review showed a significant association between antenatal stress exposure and rates of LBW. However, no statistically significant difference was found between non exposed and exposed groups relating to preterm labour. The review advocated for further studies with adequate sample size and longer follow up time.

Caffeine intake during pregnancy:

Jahanfar et al (2015) Effects of restricted caffeine intake by mother on fetal, neonatal and pregnancy outcomes: Cochrane systematic review

This review involved only one eligible study which involved 1207 pregnant women recruited before 20 weeks gestation. The first group regularly drank 3 cups of instant coffee (caffeinated). These were compared to the second group who drank the same volume of decaffeinated instant coffee. This had no effect on SGA, birth weight or preterm birth. They suggested there is currently insufficient evidence from high quality RCTs to evaluate the effect of restricted caffeine intake during pregnancy on fetal outcomes.

Chen at el (2014): Maternal caffeine intake during pregnancy is associated with risk of low birth weight: a systematic review and dose-response meta-analysis

This systematic review identified 9 prospective studies with LBW as a binary outcome variable (90,747 participants and 6,303 cases). Higher caffeine intake during pregnancy was associated with a higher risk of LBW. This increased with increasing levels of caffeine intake, suggesting a dose response. The study suggested that the risk of LBW may be elevated even for caffeine intakes below the recommended maximum limit of current guidelines for pregnant women (300mg.day by WHO and 200mg/day by Nordic and American College). Limitations lie in potential biases including that of confounding by smoking or pregnancy symptoms affecting the association seen. WHO class this review as low to moderate certainty evidence.

Pregnancy weight gain:

Low gestational weight gain and LBW:

For peer review only

Han et al (2011): Low gestational weight gain and the risk of preterm birth and low birthweight: a systematic review and meta-analyses

Low gestational weight gain and the risk of preterm birth and low birthweight: a systematic review and meta-analyses

Singleton infants born to women with low total pregnancy weight gain had higher risks of LBW and higher risks of PTB. The lower the gain, the higher the risks were. Limitations stem from few studies providing adjusted analyses or examining the combined impact of gestational weight gain and maternal weight. Authors state that the impact of low pregnancy weight gain in underweight women compared to normal weight and obese women needs more research as there may be less of a risk in heavier women

Low gestational weight gain and small for gestational age:

Goldstein et al (2018): Gestational weight gain across continents and ethnicity: systematic review and meta-analysis of maternal and infant outcomes in more than one million women.

Seven studies for USA/Europe were included in this analysis. Gestational weight gain below that of the guidelines was associated with a higher risk for small for gestational age. This study also focused on differences in ethnicity across studies but reported higher risks across all ethnicities.

High gestational weight gain and LBW:

Mcdonald et al (2011): High Gestational Weight Gain and the Risk of Preterm Birth and Low Birth Weight: A Systematic Review and Meta-Analysis

This review contained 38 studies but these mainly presented unadjusted data. Women with high total gestational weight gain had lower unadjusted risks of LBW and PTB. However, high weekly GWG was associated with increased risk. Authors said more unadjusted studies are urgently needed and more syudies with obese women and suggest the potential benefits of high gestational weight gain need to be considered against maternal risks and infant risks including high birth weight.

Pre-pregnancy BMI:

Yu et al (2013): Pre-Pregnancy Body Mass Index in Relation to Infant Birth Weight and Offspring Overweight/Obesity: A Systematic Review and Meta-Analysis

45 studies of medium to high quality were included in this review. In comparison to normal weight mothers, pre-pregnancy underweight increased the risk of low birth weight and small for gestational age. Pre-pregnancy overweight or obesity increased the risk of high birth weight (1.53,1.44-1.63) and being large for gestational age. Limitations lie in that there may be other factors not included that may mediate the association which include but are not limited to maternal age, gestational hypertension, and smoking. Authors advocate for these factors to be addressed in future studies.

Mcdonald et al (2010): Overweight and obesity in mothers and risk of preterm birth and low birth weight infants: systematic review and meta-analyses

This review found that the overall risk of LBW was decreased in women who were overweight and obese (0.8, 0.75 to 0.95). The overall risk of PTB was similar in overweight and obese women and women of normal weight but the risk of PTB before 32 weeks and induced preterm birth before 37 weeks was

For peer review only

increased in overweight and obese women. After they accounted for publication bias, the apparent protective effect of OW and obesity on LBW no longer remained, whereas risk of PTB was significantly higher in overweight and obese women (1.24,1.13 -1.37). Limitations stem from many of the included studies not adjusting for confounding variables such as gestational weight gain, socioeconomic status and smoking status. Authors argue that pre-pregnancy BMI more important than gestational weight gain.

Deprivation:

Weightman et al (2012): Social inequality and infant health in the UK: systematic review and meta-analyses

Both being in the most deprived neighbourhood and low social class increased the odds of LBW infants. Limitations include studies varying in comparison of deprivation levels and authors noted the effects of deprivation may vary between the areas where primary research studies were carried out.

Vitamin D supplementation during pregnancy

Maugeri et al (2019): Effects of Vitamin D Supplementation During Pregnancy on Birth Size: A Systematic Review and Meta-Analysis of Randomized Controlled Trials.

The meta-analysis of RCTs showed a significant positive effect of maternal vitamin D supplementation on the risk of being born small for gestational age. However, researchers suggest more RCTs are needed to better understand risks and benefits of such interventions.

**An earlier Cochrane review by De Regil et al (2016) suggested that whilst vitamin D supplementation during pregnancy may reduce the risk of having a low birth weight infant, results show that when vitamin D and Calcium are combined there is an increased risk of premature birth and data on adverse effects are not well reported.

Folic acid supplementation:

Lassi et al (2013): Folic acid supplementation during pregnancy for maternal health and pregnancy outcomes

This Cochrane review included 4 studies which looked at the association between folic acid supplementation during pregnancy and low birthweight as part of a wider group of outcomes. No impact was seen on reducing low birth weight.

A later overview of systematic reviews by Lopes et al (2017) also found folic acid supplementation did not alter the risk of premature birth or LBW.

Air pollution:

Guo et al (2019): Ambient air pollution and adverse birth outcomes: a systematic review and meta-analysis:

For peer review only

This study found that when mothers were exposed to CO, NO_2, NO_x, O_3, $PM_{2.5}$, PM_{10}, and SO_2 throughout pregnancy, there was significant association with LBW. They did suggest that future meta-analyses should take into account the extent of interactions between differing pollutants and explore issues such as economic status and disease history not accounted for in this analysis.

A study was carried out in 2014 by Hammen et al specific to the UK which found small increased risks of SGA with exposure to high concentrations of PM_{10} during pregnancy and similar effects for NO_2, $PM_{2.5}$ and CO in later pregnancy, with this association found particularly among female infants.

Maternal education level

Silvestrin et al (2013): Maternal education level and low birth weight: a meta-analysis.

High maternal education showed a 33% protective effect against low birth weight, whereas medium degree of education showed no significant protection when compared to low maternal education.

Maternal age:

Goisis et al (2017): Advanced Maternal Age and the Risk of Low Birth Weight and Preterm Delivery: a Within-Family Analysis Using Finnish Population Registers
Goisis et al (2018): Secular changes in the association between advanced maternal age and the risk of low birth weight: A cross-cohort comparison in the UK.
Findings regarding the impact of older maternal age on low birth weight have been mixed. The Finnish study by Goissis et al (2017) found that between families the risk of LBW was 1.1 (0.8-1.4) for those aged 35-39 and 2.2 (1.4-2.9) for those aged 40+. However, when they looked within families, this association disappeared. A UK cross cohort study by Goisis et al (2018) also found that in the later birth cohorts the effect of maternal age on LBW was less.

Paternal factors

Shah (2019): Paternal factors and low birthweight, preterm and small for gestational age births: a systematic review

This study identified paternal age and height to be associated with LBW. They also suggested heavy and prolonger exposure to lead aswell as low paternal education may be associated with LBW but advocated for more studies in this field.

For peer review only

Cocaine

Dos Santos et al (2018) Maternal, fetal and neonatal consequences associated with the use of crack cocaine during the gestational period: a systematic review and meta-analysis:

This study was specific to crack cocaine and included 10 studies showing crack cocaine use during pregnancy to be significantly associated with preterm birth (OR: 2.22, 1.59–3.10),, small for gestational age (4.00; 1.74–9.18) and low birth weight (2.80; 95% CI 2.39–3.27).

Smoking

Pereira at el (2017) Maternal Active Smoking During Pregnancy and Low Birth Weight in the Americas: A Systematic Review and Meta-analysis:

This review and meta analysis found similar odds ratios to that reported in the previous study by Walsh et al (1994) in the Johnson review. This review was however specific to the Americas.

Flower et al (2013) Pregnancy planning, smoking behaviour during pregnancy, and neonatal outcome: UK millennium cohort study

This is an earlier study which may be of greater relevance in terms of population. This study again found a similar odds (1.91; 1.56-2.34) for LBW for babies of mothers who were smoking just before pregnancy. Women who quit or reduced the amount they smoked during the pregnancy lowered the risk of LBW by one third compared with those whose smoking status did not change.

Gum Disease

Daalderop et el (2017): Periodontal Disease and Pregnancy Outcomes: Overview of Systematic Reviews

This review of reviews found a similar relative risk ratios to that generated by Corbella et al (2012) in the Johnson et al review. With relative risk of LBW at 1.7 (1.3-2.1), preterm birth (1.6; 1.3-2.0) and preterm low birth weight (3.4, 1.3-8.8). The review concluded that there is consistent evidence from systematic reviews indicating pregnant women with periodontal disease are at increased risk of having a LBW baby.

Cannabis

Gunn et al (2014) Prenatal exposure to cannabis and maternal and child health outcomes: a systematic review and meta-analysis

This systematic review , again found similar odds ratios of mums using cannabis during pregnancy (1.77; 1.04-1.31) to that generated by a cohort study by Hayatbakhsh et al 2012 in Johnson et al.

Intimate Partner Violence

For peer review only

Hill et al (2016) A Systematic Review and Meta-Analysis of Intimate Partner Violence During Pregnancy and Selected Birth Outcomes

Donovan et al (2016) Intimate Partner Violence During Pregnancy and the Risk for Adverse Infant Outcomes: A Systematic Review and Meta-Analysis

These two reviews carried out in 2016 found different risk ratios for the effect of intimate partner violence on low birth weight. Donovan et al (2016) found OR of 2.11 for LBW but 1.37 for SGA which was only marginally significant although meta analysis was on fewer studies. They also called to more studies examining this association as suggested a large degree of heterogeneity in LBW studies. The review by Hill et al (2016) reported much lower OR of 1.18.

Chlamydia

Olson-Chen et al (2018) Chlamydia trachomatis and Adverse Pregnancy Outcomes: Meta analysis of Patients with and without infection

The authors of this review suggest that chlamydia in pregnancy is associated with small increases in the odds of adverse pregnancy outcomes. The odds of LBW (1.34; 1.21-1.48) and small for gestational age (1.14; 1.05-1.25) were significant but authors suggest the literature is complicated by heterogeneity and associations may not hold in higher quality prospective studies.

Anemia

Figuerido et al (2018) Maternal Anemia and Low Birth Weight: A Systematic Review and Meta-Analysis

This review found a similar odds ratio (1.23) to that reported by Haider et al (2013) in Johnson review with maternal anemia a risk factor for LBW.

Alcohol

Nykjaer et al (2014) Maternal alcohol intake prior to and during pregnancy and risk of adverse birth outcomes: evidence from a British cohort

This cohort study found that over half of pregnant women in the first trimester reported alcohol intake above the Department of Health guidelines of <=2 units per week. Consuming alcohol in the first trimester was the most sensitive to developing foetus. Results showed that even women complying with government alcohol guidelines in this period were still at significantly higher risk of having LBW babies and preterm birth compared to non-drinkers.

For peer review only

Interventions for prevention of Low Birth Weight

East et al 2019 Cochrane review examined programmes offering social support during pregnancy compared with routine care for women at increased risk of low birth weight. Offering additional social support (emotional , informational and instrumental) slightly reduced the number of babies with low birth weight (0.94, 0.86-1.04) but any effect was not large.

Chamberlain et al 2017 : A Cochrane review of psychosocial interventions (counselling, health education, feedback, incentives, social support, exercise and dissemination) to stop smoking in pregnancy found counselling, feedback and incentives seem to be effective at increasing the proportion of women who stop smoking in late pregnancy. However, they suggest the context of the interventions need careful consideration. The effect of health education and social support was less clear. Woman who received psychosocial interventions had a 17% reduction in low birth weight infants.

Temel et al 2014-Evidence based preconception lifestyle interventions.

This research suggests that the list regarding interventions for which there is substantial evidence of effectiveness when applied in the preconception period is relatively short. For alcohol, evidence is lacking. Nutrition interventions show effectiveness in terms of dietary change and birth weight. Smoking interventions were shown to be effective in smoking reduction in the preconception period and individual and collective interventions to increase use of folic acid use had positive effects on behaviour change.

Thangaratinam et al 2012 Effects of interventions in pregnancy on maternal weight and obstetric outcomes: meta-analysis of randomised evidence:

This meta-analysis concluded dietary and lifestyle interventions in pregnancy are effective in reducing gestational weight gain without any adverse effect on the risk of infants born SGA. Dietary interventions were associated with the greatest reduction in pregnancy weight gain compared with physical activity and a mixed approach. Diet significantly reduced the risk of preterm birth compared with any other intervention. The rating of evidence quality in this analysis was moderate.

For peer review only

STROBE Statement—Checklist of items that should be included in reports of ***cohort studies***

	Item No	Recommendation	Page No
**Title and abstract**	1	(*a*) Indicate the study's design with a commonly used term in the title or the abstract	1
		(*b*) Provide in the abstract an informative and balanced summary of what was done and what was found	2-3
**Introduction**			
Background/rationale	2	Explain the scientific background and rationale for the investigation being reported	5
Objectives	3	State specific objectives, including any prespecified hypotheses	5
**Methods**			
Study design	4	Present key elements of study design early in the paper	6
Setting	5	Describe the setting, locations, and relevant dates, including periods of recruitment, exposure, follow-up, and data collection	6
Participants	6	(*a*) Give the eligibility criteria, and the sources and methods of selection of participants. Describe methods of follow-up	6
		(*b*) For matched studies, give matching criteria and number of exposed and unexposed	
Variables	7	Clearly define all outcomes, exposures, predictors, potential confounders, and effect modifiers. Give diagnostic criteria, if applicable	6-7
Data sources/ measurement	8*	For each variable of interest, give sources of data and details of methods of assessment (measurement). Describe comparability of assessment methods if there is more than one group	6-7
Bias	9	Describe any efforts to address potential sources of bias	6-7
Study size	10	Explain how the study size was arrived at	6
Quantitative variables	11	Explain how quantitative variables were handled in the analyses. If applicable, describe which groupings were chosen and why	
Statistical methods	12	(*a*) Describe all statistical methods, including those used to control for confounding	7-9
		(*b*) Describe any methods used to examine subgroups and interactions	
		(*c*) Explain how missing data were addressed	
		(*d*) If applicable, explain how loss to follow-up was addressed	
		(*e*) Describe any sensitivity analyses	
**Results**			
Participants	13*	(a) Report numbers of individuals at each stage of study—eg numbers potentially eligible, examined for eligibility, confirmed eligible, included in the study, completing follow-up, and analysed	9
		(b) Give reasons for non-participation at each stage	
		(c) Consider use of a flow diagram	
Descriptive data	14*	(a) Give characteristics of study participants (eg demographic, clinical, social) and information on exposures and potential confounders	9
		(b) Indicate number of participants with missing data for each variable of interest	
		(c) Summarise follow-up time (eg, average and total amount)	
Outcome data	15*	Report numbers of outcome events or summary measures over time	9-10

For peer review only

For peer review only - http://bmjopen.bmj.com/site/about/guidelines.xhtml

Main results	16	(*a*) Give unadjusted estimates and, if applicable, confounder-adjusted estimates and their precision (eg, 95% confidence interval). Make clear which confounders were adjusted for and why they were included<br>(*b*) Report category boundaries when continuous variables were categorized<br>(*c*) If relevant, consider translating estimates of relative risk into absolute risk for a meaningful time period	9,10-11
Other analyses	17	Report other analyses done—eg analyses of subgroups and interactions, and sensitivity analyses	10
**Discussion**			
Key results	18	Summarise key results with reference to study objectives	12
Limitations	19	Discuss limitations of the study, taking into account sources of potential bias or imprecision. Discuss both direction and magnitude of any potential bias	13
17Interpretation	20	Give a cautious overall interpretation of results considering objectives, limitations, multiplicity of analyses, results from similar studies, and other relevant evidence	12-13
Generalisability	21	Discuss the generalisability (external validity) of the study results	12-14
**Other information**			
Funding	22	Give the source of funding and the role of the funders for the present study and, if applicable, for the original study on which the present article is based	14-15

*Give information separately for exposed and unexposed groups.

Note: An Explanation and Elaboration article discusses each checklist item and gives methodological background and published examples of transparent reporting. The STROBE checklist is best used in conjunction with this article (freely available on the Web sites of PLoS Medicine at http://www.plosmedicine.org/, Annals of Internal Medicine at http://www.annals.org/, and Epidemiology at http://www.epidem.com/). Information on the STROBE Initiative is available at http://www.strobe-statement.org.

For peer review only
